# Microenvironment‐Driven Mast Cell Plasticity: Insights From Cytokine‐Activated Gene Signatures in Skin and Respiratory Diseases

**DOI:** 10.1111/all.70052

**Published:** 2025-09-10

**Authors:** Chiara Tontini, Rajia Bahri, Andrew Higham, Dave Singh, Angela Simpson, Silvia Bulfone‐Paus

**Affiliations:** ^1^ Department of Musculoskeletal and Dermatological Sciences, Faculty of Biology, Medicine and Health, Lydia Becker Institute of Immunology and Inflammation The University of Manchester Manchester UK; ^2^ Division of Immunology, Immunity to Infection and Respiratory Medicine, School of Biological Sciences, Faculty of Biology, Medicine and Health University of Manchester Manchester UK; ^3^ Manchester University NHS Foundation Trust Manchester UK; ^4^ Medicines Evaluation Unit, Manchester University NHS Foundation Trust University of Manchester Manchester UK

**Keywords:** artificial intelligence, cytokines, IFN, IL‐33, IL‐4, machine learning, mast cells, omics, TGFβ, transcriptomics

## Abstract

Mast cells (MCs) rapidly adapt to the microenvironment due to the plethora of cytokine receptors expressed. Understanding microenvironment‐primed immune responses is essential to elucidate the phenotypic/functional changes MCs undergo, and thus understand their contribution to diseases and predict the most effective therapeutic strategies. We exposed primary human MCs to cytokines mimicking a T1/pro‐inflammatory (IFNγ), T2/allergic (IL‐4 + IL‐13), alarmin‐rich (IL‐33) and pro‐fibrotic/pro‐tolerogenic (TGFβ) microenvironment. We investigated MC surface receptor expression, activation, cytokine, histamine, and prostaglandin D2 release, and performed transcriptomics to define shared and unique genetic features. Using machine learning, we extracted minimal cytokine‐activated signatures and performed gene set variation analysis (GSVA), single‐cell clustering, and pseudotime analyses on tissue MCs from skin and respiratory diseases. MCs exposed in vitro to IFNγ acquire an antigen‐presenting phenotype (HLA‐DR+), increase IgE‐mediated responses and histamine release, while TGFβ inhibits activation and boosts integrin αvβ3 expression. IL‐33 primarily drives cytokine (GM‐CSF, IL‐5, IL‐10, IL‐13) and chemokine production (IL‐8, MCP‐1, MIP‐1α) and facilitates mixed IgG‐IgE responses. Among uniquely expressed genes, 245 were highly informative to discriminate cytokine‐primed MCs. GSVA revealed MC IL‐4 + IL‐13 signatures enriched in atopic dermatitis and psoriasis, IFNγ in COVID‐19 infection and cystic fibrosis, IL‐33 in COVID‐19 and chronic obstructive pulmonary disease (COPD) and TGFβ in pulmonary fibrosis (PF) and chronic rhinosinusitis. Furthermore, we detected positive IL‐33/TGFβ priming in eosinophil‐high COPD. Minimal cytokine‐activated signatures identified disease‐cytokine‐specific MC clusters and pseudotime trajectories, suggesting involvement of MCs in fibrosis (COPD/PF), T1/alarmin‐driven inflammation (COVID‐19) and mixed T1/T2 inflammatory responses (AD/psoriasis). In conclusion, in cytokine‐driven settings, MCs are phenotypically and functionally diverse. Thus, unique MC signatures will help to identify cytokine‐primed MCs and predict the efficacy of anti‐cytokine treatment in MC‐driven diseases.

## Graphical abstract

Primary peripheral blood‐derived human mast cells exposed to IFNγ, IL‐33, IL‐4 + IL‐13, or TGFβ develop distinct transcriptional signatures with unique functional changes including degranulation and cytokine/chemokine release. Machine learning‐defined minimal cytokine‐activated signatures (245 genes) successfully detect cytokine‐primed mast cells in diseased tissues using gene set variation analysis. Single‐cell pseudotime analysis reveals tissue mast cells exist along activation continua rather than discrete populations, with disease‐specific trajectories. AD, atopic dermatitis; CBA, cytometric bead array; CF, cystic fibrosis; COPD, chronic obstructive pulmonary disease; ELISA, enzyme‐linked immunosorbent assay; GM‐CSF, granulocyte‐macrophage colony‐stimulating factor; IFNγ, interferon gamma; IL, interleukin; PF, pulmonary fibrosis; PGD2, prostaglandin D2; scRNAseq, single cell RNA sequencing; TGFβ, transforming growth factor beta.

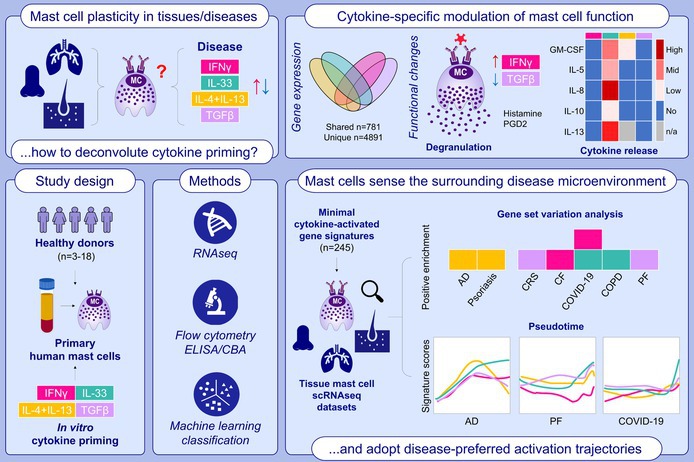

**Abbreviations**
αIgE: anti‐human IgE antibody
CD107a: lysosomal‐associated membrane protein‐1 (LAMP‐1)
CD117: stem cell factor receptor (c‐KIT)
CD63: lysosomal‐associated membrane protein 3 (LAMP‐3)
FcγRII/CD32: low‐affinity immunoglobulin G receptor II
FcεRI: high affinity immunoglobulin E receptor
GM‐CSF: granulocyte‐monocyte colony stimulating factor
HLA‐DR: major histocompatibility complex class II DR alpha
IFNγ: interferon gamma
IgE: immunoglobulin E
IL‐10: interleukin 10
IL‐13: interleukin 13
IL‐33: interleukin 33
IL‐4: interleukin 4
IL‐5: interleukin 5
IL‐8: interleukin 8/CXCL8
MCP‐1/CCL2: monocyte chemoattractant protein‐1
MCs: mast cells
MIP‐1α/CCL3: macrophage inflammatory protein‐1 alpha
MRGPRX2: Mas‐Related G Protein‐Coupled Receptor‐X2
NF‐κB: nuclear factor‐kappa B
TGFβ: transforming growth factor beta

## 1 Introduction

Mast cells (MCs) are immune effector cells known to participate in broad mucosal barrier responses [1]. Beyond allergy, MCs play a role in the promotion of inflammation, neuro‐immune interactions, and tissue remodeling observed in skin and respiratory diseases, such as atopic dermatitis (AD), psoriasis, asthma, chronic obstructive pulmonary disease (COPD) and pulmonary fibrosis (PF) [2, 3, 4, 5].

MCs constitutively express the stem cell factor receptor c‐KIT (CD117) and the high‐affinity IgE receptor FcεRI. In response to different stimuli, such as allergens engaging the IgE‐FcεRI complex or molecules engaging the Mas‐Related G Protein‐Coupled Receptor‐X2 (MRGPRX2), they release pre‐formed vasoactive mediators (e.g., histamine, tryptase, chymase, proteases) and newly‐formed cytokines and chemokines (e.g., GM‐CSF, IL‐5, IL‐13, IL‐8, IL‐10, MCP‐1/CCL2, MIP‐1α/CCL3) [6, 7]. Along with lineage‐specific markers, MCs are equipped with a plethora of cytokine receptors sensing the surrounding microenvironment and adapting MC functions accordingly [7]. Hence, MCs demonstrate a high degree of functional plasticity, as cytokine exposure influences both degranulation and their cytokine/chemokine‐producing potential [7].

MCs are present in virtually all connective tissues as a relatively stable population [8], but their representation in studies can be insufficient due to the dissociation methods used, as they are especially prone to cell death/activation due to mechanical stress and de‐differentiation when placed in culture [9, 10]. To overcome this, alternative sources to tissue MCs have been developed to study human MC function, namely cell lines and primary human MCs [10].

Recently, omics analyses have greatly enhanced our understanding of the immune landscape [11] and the role of MCs in health and diseases like allergy, autoimmunity, and cancer [12, 13].

However, despite the wealth of data, some studies fail to detect sizeable MC populations, possibly because protocols are not always optimized for MC retrieval, which could lead to zero‐read artifacts and underestimate the true biological contribution of MCs in disease [14, 15]. Common methods to analyze omics datasets include gene enrichment scores, which assess the likelihood of a specific biological pathway to be specifically up‐ or down‐regulated in the populations of interest.

However, current enrichment scores rely on knowledge‐based gene sets often lacking adequate representation of MCs [16], or derived from animals/cell lines that do not accurately replicate physiological responses in humans [10]. Recently, cell‐specific IL‐33‐activated gene signatures were described using primary human MCs and other immune cells [17], and MC signatures were found enriched in asthma subsets using gene set variation analysis (GSVA) [18].

Despite the promising progress made, we still lack the ability to fully understand and study MC plasticity. A comprehensive overview of the effect of different cytokines on MC function remains incomplete and, if available, is particularly difficult to extract the true biological significance from noise caused by biological/technical variation or random occurrence [19]. Developing unique cell‐specific cytokine signatures could help predict the impact of the local microenvironment on MCs. This approach could be especially valuable in assisting clinical therapeutic decisions, such as identifying the most efficient strategy to minimize MC involvement by selecting the optimal anti‐cytokine biologic treatment for MC‐driven diseases.

To address this knowledge gap, we generated primary human MCs from peripheral blood CD117+ progenitors [20] and exposed them to different cytokine combinations mimicking a T1/pro‐inflammatory (IFNγ), T2/pro‐allergic (IL‐4 + IL‐13), alarmin‐rich (IL‐33) and pro‐fibrotic/anti‐inflammatory (TGFβ) environment. We then identified unique genetic signatures associated with each condition and provided evidence of plasticity by analyzing the modulation of surface receptor expression and functional changes in MC responses. Minimal gene signatures were then defined using feature selection on uniquely expressed genes per cytokine and used to construct an enrichment score to measure MC cytokine priming in different diseases and assist in single‐cell analyses.

Our results demonstrated positive enrichment of MC‐cytokine‐specific signatures in different skin and respiratory diseases, highlighting the potential for targeted anti‐cytokine treatment with a focus on the modulation of MC activities.

## 2 Materials and Methods

Detailed material and methods are provided as Supporting Information. Experimental workflow is summarized in Figure S1.

### 2.1 Generation of Primary Human MCs

Primary human MCs were generated from CD117+ MC progenitors isolated from whole blood collected from anonymous donors (UREC ref. 2018‐2696‐5711) via density gradient separation (Ficoll‐Paque) and positive magnetic bead selection (CD117 microbeads, Miltenyi Biotech).

Cells were cultured for 8–10 weeks in IMDM + GlutaMAX media containing 0.5% BSA, 1% Insulin‐transferrin, 50 mM β‐Mercaptoethanol, 100 U/mL Penicillin, 100 μg/mL Streptomycin, 100 ng/mL recombinant human SCF, 50 ng/mL recombinant human IL‐6 (Genscript), and 10 ng/mL recombinant human IL‐3 (Peprotech). From Week 5, IL‐3 concentration was progressively reduced and completely removed by Week 6. MC maturity and function were assessed by flow cytometry for surface markers (CD117 and FcεRI) and degranulation markers (CD63 and CD107a; Biolegend) after sensitization with 1 μg/mL human myeloma IgE (CalBiochem) for 16 h and stimulation with 1 μg/mL anti‐human IgE for 1 h (KPL). Donors were selected if showing ≥ 90% CD117+FcεRI+ expression and/or upregulation of CD63/CD107a in anti‐IgE stimulated samples in above 70% of gated live cells (Figure S2).

### 2.2 Cytokine Priming

Mature primary human MCs were primed with 50 ng/mL IFNγ, 50 ng/mL IL‐33, 10 ng/mL IL‐4 plus 10 ng/mL IL‐13, or 5 ng/mL TGFβ (Peprotech) for 24 h or left unstimulated. Cells, supernatants, and lysed pellets were used for downstream analyses.

### 2.3 Bulk RNA Sequencing of Human MCs Primed With Cytokines

RNA from cytokine‐primed MCs (*n* = 4) was extracted using a commercially available total mRNA extraction kit (RNAeasy micro kit; Qiagen). Samples were submitted for next‐generation sequencing (HiSeq4000; Illumina) to the Genomics Core Technology Facility of the University of Manchester.

### 2.4 Bioinformatics Analysis Pipeline and Gene Set Enrichment

Raw RNAseq FASTQ files underwent quality control (FastQC) [21], trimming (BBDuk) [22], and alignment to the human hg38 reference genome using STAR v2.7.7a [23]. Differential gene expression analysis was performed using DESeq2 v1.30.1 [24], with significance set at Bonferroni‐Hochberg false discovery rate (FDR) adjusted *p*‐value < 0.05. Counts were normalized using DESeq2's median of ratios approach. Gene enrichment analyses were conducted using Metascape [25].

### 2.5 Surface Receptor Staining

Following cytokine priming, MCs (*n* = 3–7) were incubated with Fc receptor blocking solution (1:100) and live/dead marker (1:1000) for 15 min, then stained with antibodies (1:200 final concentration) for CD117, FcεRI, MRGPRX2, FcγRII/CD32, FcγRIIb‐c/CD32b‐c, HLA‐DR, and Integrin αvβ3 (Biolegend) and measured in flow cytometry (BD Biosciences).

### 2.6 Mast Cell Activation Test (MAT)

MCs (*n* = 4) were sensitized overnight with 100 ng/mL human myeloma IgE and then stimulated with 50 ng/mL anti‐human IgE for 1 h. For IgG‐FcεRI immunocomplex activation, IL‐33‐primed or unstimulated MCs (*n* = 4) were pre‐treated with human Fc blocking solution (1:100), then stimulated with 1 μg/mL mouse anti‐FcεRI antibody, with or without 1 μg/mL IgG2a kappa mouse antibodies, and cross‐linked using 5 μg/mL anti‐mouse lambda chain antibody. Degranulation was assessed by flow cytometry using CD63 and CD107a expression on live MCs.

### 2.7 Histamine, Prostaglandin D2, Cytokine/Chemokine Release in Cell Culture Supernatants

Histamine and prostaglandin D2 release from 24 h cytokine‐primed MCs (*n* = 5), sensitized overnight with 100 ng/mL IgE and stimulated with 50 ng/mL anti‐IgE for 1 h, was measured in cell culture supernatants using commercial ELISA kits (Abnova, Caiman Chemicals). The release of cytokines (GM‐CSF, IL‐5, IL‐10, IL‐13) and chemokines (IL‐8, CCL2/MCP‐1, CCL3/MIP‐1α) from harvested cell culture supernatants (*n* = 14–18) was quantified using cytometric bead array (BD Biosciences).

### 2.8 Immunofluorescence

Peripheral lung tissue from *n* = 1 COPD patient undergoing surgical resection for lung cancer was obtained under written informed consent with protocols approved by the South Manchester Ethics Committee (03/SM/396) and the Northwest Ethics Committee (20/NW/0302). Immunofluorescence staining was performed using 1:800 anti‐human Tryptase (clone AA1; Abcam), 1:50 anti‐human IL‐13 (clone OTI6D3; Thermo Fisher Scientific), and 1:100 IL‐33 (clone Nessy‐1; Enzo Life Sciences), following heat‐induced epitope retrieval at sequentially increasing pH (Fisher Scientific). Fluorescent staining was performed using Alexa Fluor‐conjugated anti‐mouse or anti‐rabbit antibody kits (Thermo Fisher Scientific), and nuclear counterstaining with Hoechst 33258.

### 2.9 Feature Selection

To identify minimal cytokine‐activated gene signatures, the machine learning algorithm Elastic Net was applied to 3873 significant protein‐coding genes not shared across different cytokines (R packages glmnet and caret) [26, 27]. The dataset was partitioned (70/30) into training and testing sets, and the model was optimized (*α* = 0.5, *λ* = 0.007976564). The identified 281 genes (unstimulated = 36, IFNγ = 69, IL‐33 = 78, IL‐4 + IL‐13 = 26, TGFβ = 72 genes) were further validated by training a Neural Network model (package neuralnet) [28] on a 50/50 partitioned dataset, assessing accuracy, class‐specific sensitivity, specificity, and generating multiclass ROC curves.

### 2.10 Re‐Analysis of Single‐Cell RNAseq Public Datasets

Publicly available single‐cell RNAseq (scRNAseq) (GSE201153, GSE179633, GSE179633; Zenodo: 5228495; CellXGene: https://cellxgene.cziscience.com/collections/6f6d381a‐7701‐4781‐935c‐db10d30de293) and bulk RNAseq (GSE140900, GSE217060) datasets were used for validating and testing the identified minimal cytokine‐activated MC signatures in tissue MCs and various diseases. Among the testing datasets, we re‐analyzed the integrated Human Lung Cell Atlas (*n* = 95 lung/airway, *n* = 9 nasal mucosa samples) [29], the Liu et al. [30] skin scRNAseq dataset (*n* = 7 healthy, *n* = 7 atopic dermatitis, *n* = 8 psoriasis), and the West et al. [31] bulk RNAseq dataset of isolated skin MCs (GSE217060, *n* = 7 healthy, *n* = 6 psoriasis). Single‐cell RNAseq data of the identified tissue MC populations were processed using Seurat v5 [32] and differential gene expression analysis was performed using the Wilcoxon rank sum test adjusted using the FDR method.

### 2.11 Pseudotime Cluster Analysis of Single Cell Data

Pseudotime trajectory analysis was conducted using Slingshot v2.0 [33] on UMAP embeddings to infer progression paths between MC clusters. Natural binning was implemented, and trajectory phases were subdivided into tertiles (early, mid, late). Disease‐specific trajectory enrichment was assessed using FDR‐adjusted Fisher's exact tests. Single‐cell cytokine pathway signature scores were calculated as the mean log‐normalized expression of pathway‐specific gene sets, and differences between disease and healthy cells were compared using FDR‐corrected t‐tests.

### 2.12 Extraction of Pseudobulk Tissue Mast Cell Counts and Gene Set Variation Analysis (GSVA)

Pseudobulk raw counts were generated from single‐cell counts using summarizeAssayByGroup (scater package) [34] and normalized/log_2_ transformed using the EdgeR package [35]. For bulk RNAseq datasets, normalized log_2_ counts were extracted from DESeq2. Gene Set Variation Analysis (GSVA v1.38) [18] was performed using the minimal cytokine‐activated gene signatures to retrieve enrichment scores. A linear model was fit, and moderated t‐statistics were computed using the eBayes function of the limma package [36] to calculate differences between healthy and disease/conditions for GSVA scores, adjusted for multiple comparisons using the FDR method.

### 2.13 Manual Gene Expression Scores Using Minimal Cytokine‐Activated Signatures

The minimal cytokine‐activated signatures were further validated by calculating manual gene expression scores in a previously published bulk RNAseq dataset of bronchial brushings from *n* = 20 blood eosinophil‐high and *n* = 17 eosinophil‐low COPD patients [37]. Normalized counts for the 245 protein‐coding genes identified using Elastic Net were extracted, and average expression per gene was calculated. Standardized counts per cytokine‐activated gene were then averaged per subject, and unpaired *t*‐tests, Spearman correlation, and linear regression analyses were performed to compare signatures between COPD subjects with high and low blood eosinophil counts using Prism 10 (Graphpad).

### 2.14 Statistical Analyses

For surface receptor, cytokine measurement, and MAT assays, repeated measures one‐way ANOVA with Dunnett's correction was used, while for histamine and prostaglandin D2 concentrations, the Friedman test with Dunn's correction was chosen. Only ANOVA/Friedman test results with *p* < 0.05 were used for post hoc multiple comparison analyses. For bulk RNAseq and GSVA analyses, testing on paired samples and/or multiple comparisons was analyzed using the statistical methods previously detailed, and corrected using the Benjamini‐Hochberg FDR method. For all post hoc statistical analyses, significance was set at an adjusted *p* value < 0.05.

## 3 Results

### 3.1 Cytokine Stimulations Exert Unique and Shared Transcriptional Changes in Primary Human MCs

To better understand the impact of the cytokine microenvironment on MCs at a transcriptomic level, we incubated primary human MCs with different cytokine stimulation conditions (IFNγ, IL‐33, IL‐4 + IL‐13, TGFβ) for 24 h and extracted mRNA for bulk sequencing.

**FIGURE 1:**
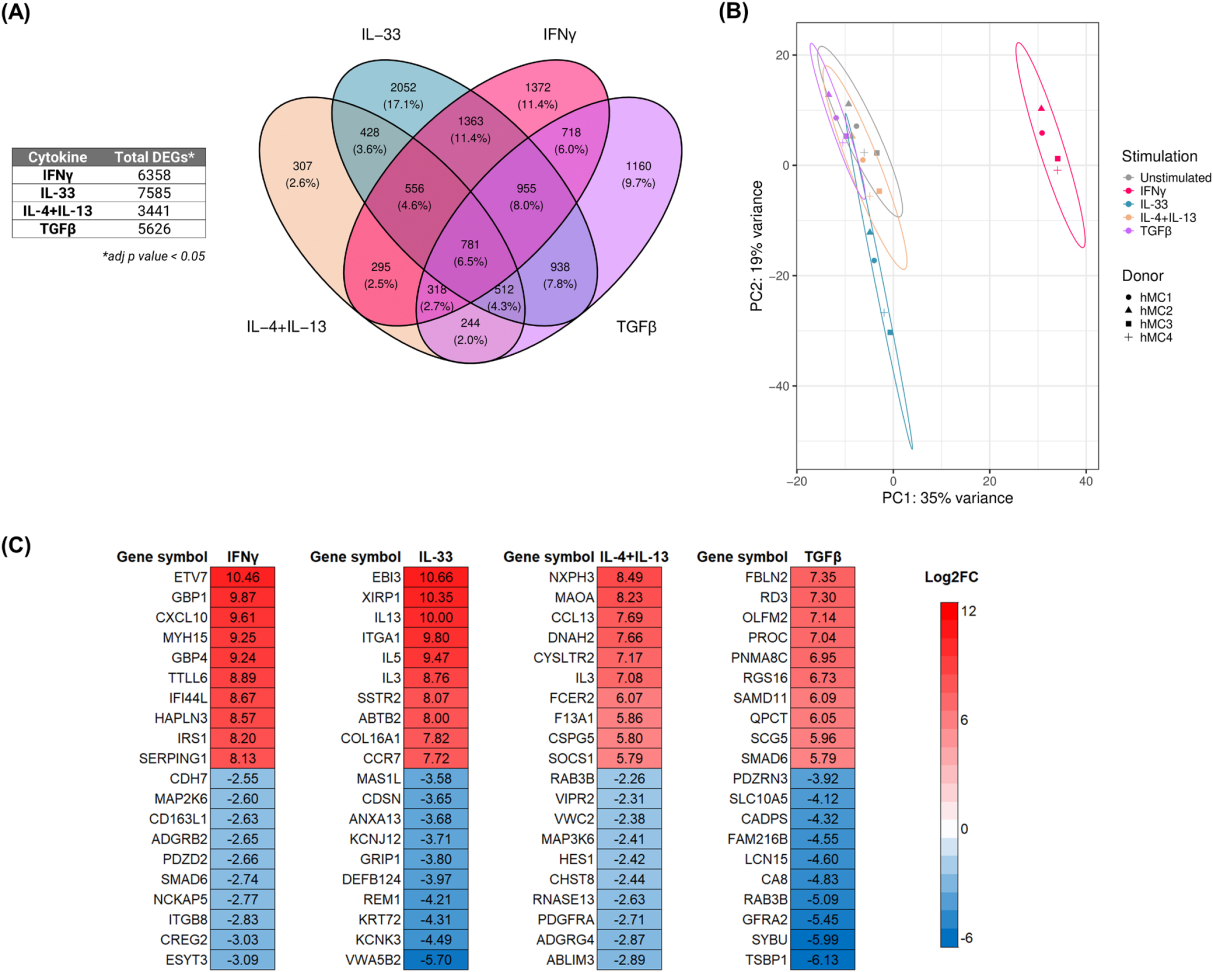
Transcriptomic changes upon MC stimulation with IFNγ, IL‐33, IL‐4 + IL‐13 and TGFβ. Primary human MCs (*n* = 4) were incubated for 24 h with IFNγ 50 ng/mL, IL‐33 50 ng/mL, IL‐4 + IL‐13 10 ng/mL and TGFβ 5 ng/mL. Total mRNA was extracted for bulk sequencing and analyzed using DESeq2 [24]. (A) Venn diagram of differentially expressed genes (false discovery rate adjusted *p* < 0.05, DEGs) per stimulation was created using the R package ggvenn [38]; (B) principal component analysis of the analyzed dataset was created using R prcomp function and plotted using ggplot2 [38]; (C) log_2_ fold change of top 10 upregulated and downregulated genes per each cytokine stimulation.

A total of 11,999 unique genes were identified as differentially expressed across all cytokine stimulations, with IL‐33 displaying the highest number of differentially expressed genes (DEGs, Figure 1A). Principal component analysis revealed that MCs exposed to IFNγ and IL‐33 diverged the most from unstimulated MCs (Figure 1B). Top expressed genes per cytokine stimulation included ETV7, GBP1, and CXCL10 for IFNγ, EBI3, XIRP1, and IL13 for IL‐33, NXPH3, MAOA, CCL13 for IL‐4 + IL‐13, and FBLN2, RD3, and OLFM2 for TGFβ, respectively (Figure 1C, Table S1). Of note, 59.2% of differentially expressed genes were shared by at least two cytokines, with a core of 781 genes commonly regulated across all stimulations (Figure 1A). We observed enriched representation of 15 anaphylaxis‐associated pathways and identified 15 genes involved in IgE/FcεRI signaling that were convergently modulated by IFNγ, IL‐33, IL‐4 + IL‐13, and TGFβ (see Figures S3 and S4).

**FIGURE 2:**
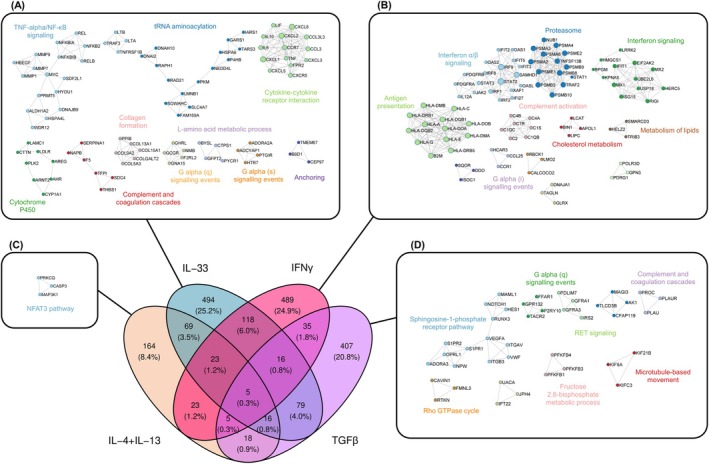
Protein–protein interaction networks reveal unique upregulated cytokine‐specific MC functions. Primary human MCs (*n* = 4) were incubated for 24 h with IFNγ 50 ng/mL, IL‐33 50 ng/mL, IL‐4 + IL‐13 10 ng/mL and TGFβ 5 ng/mL. Total mRNA was extracted for bulk sequencing and analyzed using DESeq2. Protein–protein interaction networks of uniquely upregulated protein‐coding genes for IL‐33 (A), IFNγ (B), IL‐4 + IL‐13 (C) and TGFβ (D) were computed using Metascape and visualized using Cytoscape. NFAT3, nuclear factor of activated T‐cells 3; NF‐kB, nuclear factor kappa‐light‐chain‐enhancer of activated B cells.

To specifically address the unique features associated with each cytokine stimulation, we conducted gene enrichment analysis of the genes uniquely upregulated by IFNγ, IL‐33, IL‐4 + IL‐13, and TGFβ (Figure S5A). Several shared pathways were found enriched, particularly between IFNγ and IL‐33 (Figure S5B,C). Key transcriptional regulators identified in response to IFNγ included the MHC class II transactivator CIITA, the RFX complex (RFXANK, RFXAP, RFX5), CREB5, and STAT2; to IL‐33 JUN, FOS, CEBPB; to IL‐4 + IL‐13 GATA‐1; and to TGFβ HOXD3, FOSL1, FOXM1, and RUNX2 (Figure S5D). Protein–protein interaction (PPI) network analysis [39] for each cytokine revealed main hub genes involved in broad cytokine responses (Figure 2). Networks unique to IFNγ showed significant representation of interferon signaling pathways, along with antigen presentation/proteasome activity and complement activation. In contrast, IL‐33 highlighted cytokine‐cytokine receptor interactions, nuclear factor‐kappa B (NF‐κB) signaling, tRNA aminoacylation, and cytochrome P450 networks. The NFAT3 pathway was the only enriched network observed upon IL‐4 + IL‐13 stimulation, while the sphingosine‐1‐phosphate receptor pathway, including integrin αvβ3 (ITGAV/ITGB2) was the most represented network upregulated by TGFβ.

Despite the considerable overlap observed in knowledge‐based enrichment scores across stimulations, the identified PPI networks and enrichment scores suggest distinct cytokine‐specific functional differences in MCs. Specifically, IFNγ promotes antigen presentation/processing, IL‐33 acts as a cytokine/chemokine inducer, and TGFβ boosts sphingosine‐1‐phosphate response elements.

### 3.2 Cytokines Modulate Transcription and Surface Expression Levels of Mast Cell Activating Receptors

**FIGURE 3:**
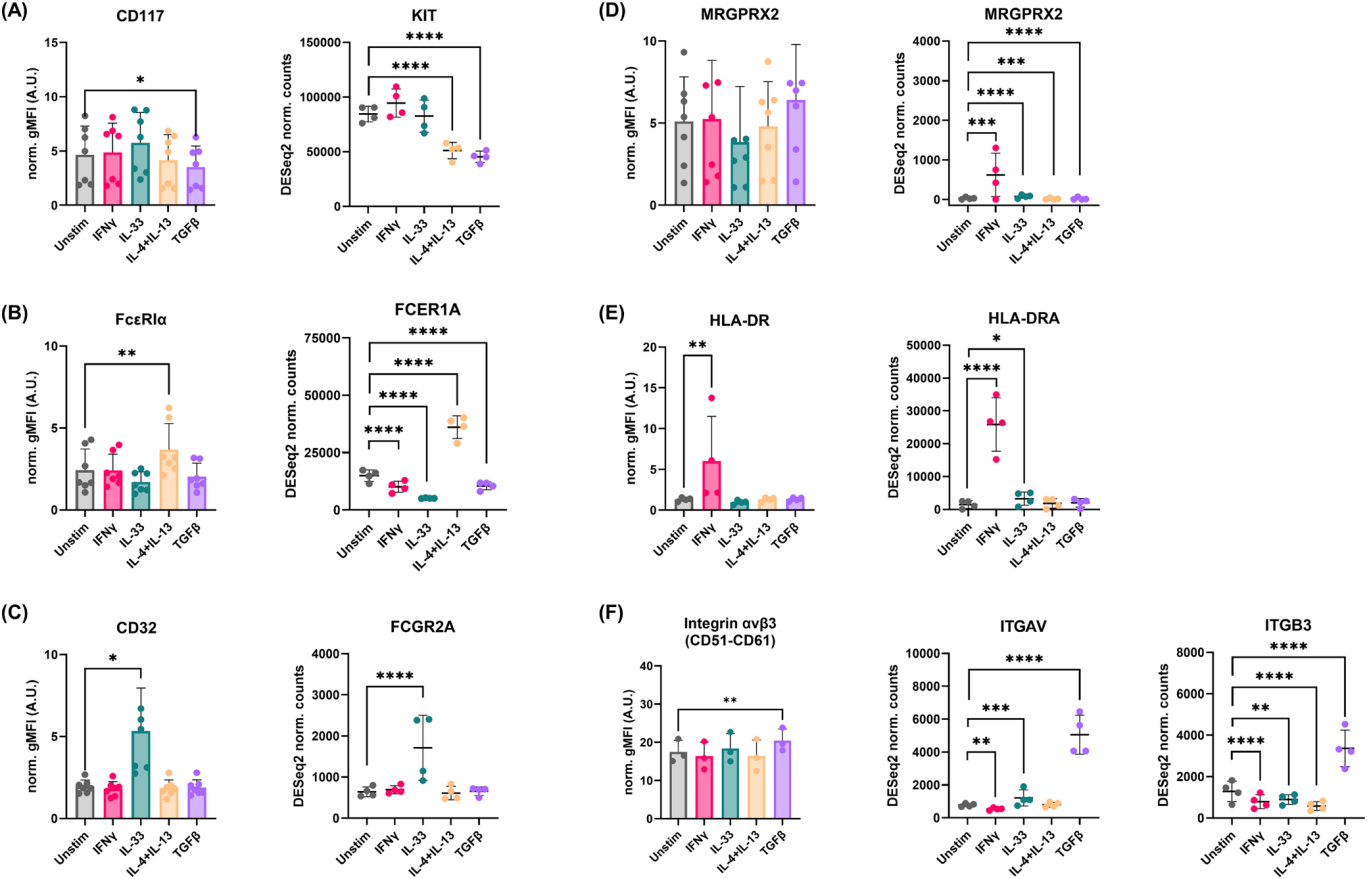
MC surface receptor expression changes in response to cytokine priming parallel the transcriptomics findings. Primary human MCs (*n* = 3–7) were incubated for 24 h with IFNγ 50 ng/mL, IL‐33 50 ng/mL, IL‐4 + IL‐13 10 ng/mL and TGFβ 5 ng/mL. Total mRNA was extracted for bulk sequencing (*n* = 4). Differential gene expression compared to unstimulated (adjusted by the false discovery rate method) and normalized counts were calculated using DESeq2's median of ratios approach. Surface receptor expression was measured via flow cytometry, and geometric mean expression normalized by the unstained control was calculated per each donor (A.U., arbitrary units). Repeated measures one‐way ANOVA with Dunnett's correction was used for statistical comparisons between different cytokines and the unstimulated control. For ANOVA, significance was set at *p* < 0.05, while for multiple comparisons adjusted *p* values < 0.05 were considered significant. Surface expression (left) and normalized transcriptomic counts (right) are displayed for CD117/KIT (A), FcεRI/FCER1A (B), FcγRII/FCGR2A (C), MRGPRX2 (D), HLA‐DR (E), Integrin αvβ3/ITGAV and ITGB3 (F). Data presented as mean ± standard deviation. Adjusted *p < 0.05, **p < 0.01, ***p < 0.001, ****p < 0.0001.

To define the phenotypic changes of cytokine‐primed MCs and validate the in vitro transcriptomics signatures, we measured the differences in expression of MC activating receptors, specifically CD117, FcεRIα, MRGPRX2, and the low‐affinity IgG receptor II (FcγRII/CD32) following 24 h incubation with IFNγ, IL‐33, IL‐4 + IL‐13, or TGFβ in flow cytometry (gating strategy in Figure S6) compared to single gene expression results. Surface staining experiments (left) confirmed some of the transcriptomic findings for CD117, FcεRIα and FcγRII/CD32 (right), indicating direct modulation mediated by TGFβ, IL‐4 + IL‐13, and IL‐33, respectively (Figure 3A–C). Since MCs were negative for FcγRIIb‐c staining (data not shown), the majority of CD32 expression was given by the activating FcγRIIa receptor. Conversely, we did not observe significant changes in MRGPRX2 expression due to high donor‐dependent variability, which was not influenced by cytokine exposure (Figure 3D).

Additionally, unique surface proteins identified via PPI network analysis were also investigated, namely the major histocompatibility molecule HLA‐DR and integrin αvβ3 (CD51/CD61), found highly expressed in response to IFNγ and TGFβ stimulations, respectively (Figure 3E,F).

Despite the fact that cytokine exposure modulates the intensity of expression of each investigated receptor, all but HLA‐DR are expressed in different proportions by unstimulated MCs (Table S2).

These results further confirm the phenotypic differences in receptor expression induced by cytokines. In particular, exposure to IL‐33 in MCs drives the expression of low‐affinity IgG receptor, IL‐4 + IL‐13 increase FcεRIα, IFNγ promotes antigen presenting HLA class II molecules, and TGFβ enhances integrin αvβ3 expression.

### 3.3 IFNγ and TGFβ Modulate IgE‐Mediated Activation, While IL‐33 Enhances Mixed FcεRI‐IgG Responses and Cytokine/Chemokine Release

**FIGURE 4:**
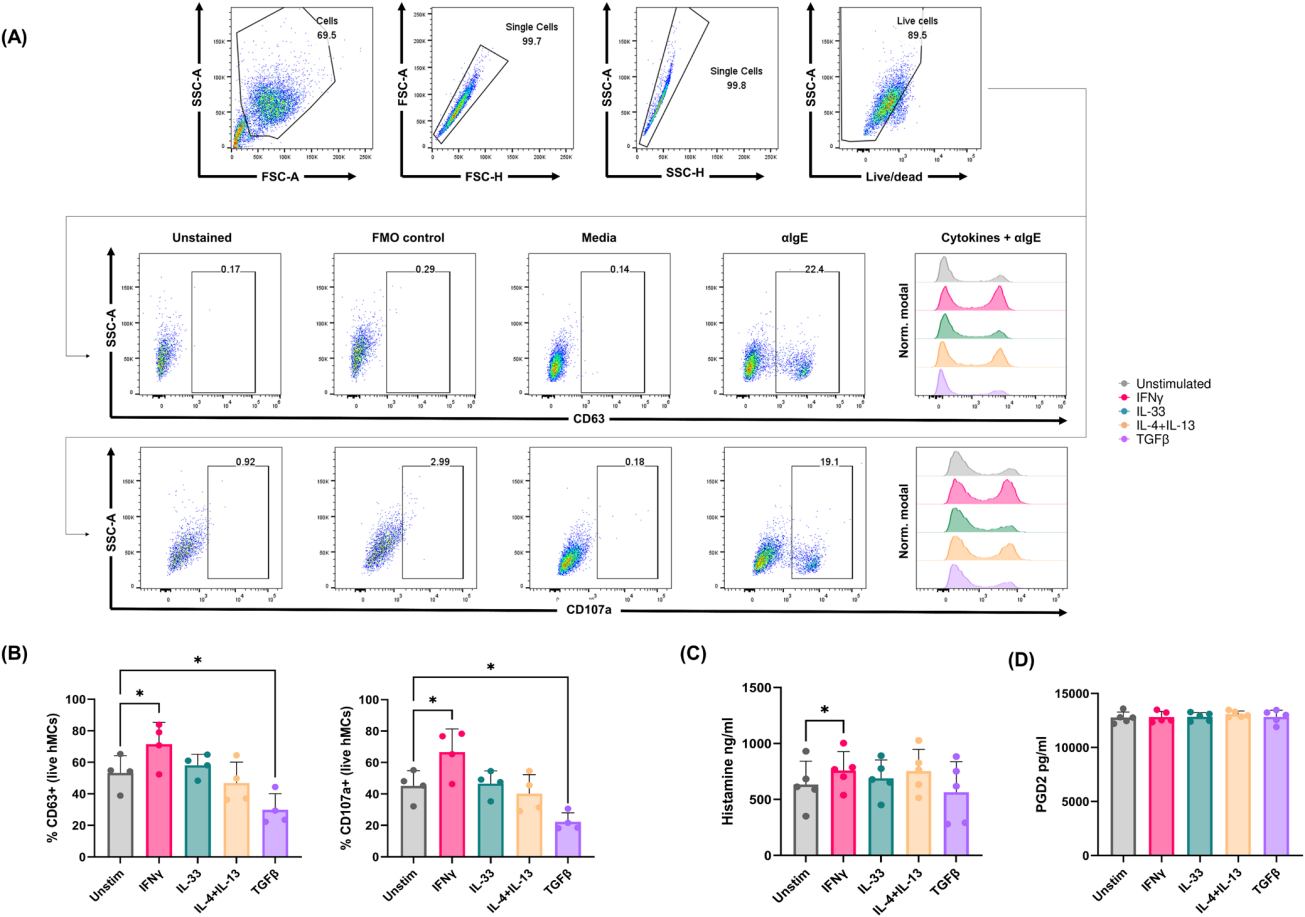
MC activation and histamine release are influenced by IFNγ and TGFβ priming. Primary human MCs (*n* = 4–5) were incubated for 24 h with IFNγ 50 ng/mL, IL‐33 50 ng/mL, IL‐4 + IL‐13 10 ng/mL and TGFβ 5 ng/mL, while sensitized overnight with human myeloma IgE 100 ng/mL. Cells were washed and stimulated with anti‐IgE (αIgE) 50 ng/mL for 1 h. (A) Representative plots from *n* = 1 IgE/anti‐IgE experiment were generated using FlowJo. MC samples were stained with fluorochrome‐conjugated antibodies against CD63 and/or CD107a, alongside unstained and fluorescence minus one (FMO) controls. (B) Upregulation of activation markers CD63 and CD107a was measured in flow cytometry. Repeated measures one‐way ANOVA with Dunnett's correction was used for the statistical comparisons between different cytokines and the unstimulated control. For ANOVA, significance was set at *p* < 0.05, while for multiple comparisons adjusted *p* values < 0.05 were considered significant. (C) Histamine and (D) prostaglandin D2 release were measured on culture supernatants using ELISA. Friedman test with Dunn's correction was used for comparisons between different cytokines and the unstimulated control. For Friedman, significance was set at *p* < 0.05, while for multiple comparisons adjusted *p* values < 0.05 were considered significant. Data presented as mean ± standard deviation. Adjusted *p < 0.05.

To define the functionality of cytokine‐primed MCs, we incubated MCs for 24 h with IFNγ, IL‐33, IL‐4 + IL‐13, and TGFβ and engaged the IgE‐FcεRI axis by sensitizing the cells with human IgE and exposing them to anti‐IgE (αIgE) for 1 h (Figure 4A, Figure S7A).

After 24 h we could observe a significant increase in MC activation measured by CD63 and CD107a upregulation compared to unstimulated controls with IFNγ priming (+18.2% CD63+ MCs, *p* = 0.0124; +21.5 CD107a+ MCs, *p* = 0.0147), while TGFβ exerted the opposite effect (−23.4% CD63+ MCs, *p* = 0.0116; −22.9 CD107a+ MCs, *p* = 0.0169, Figure 4B, Figure S7B). Conversely, histamine release significantly increased only in the presence of IFNγ stimulation (+126.7 ng/mL, *p* = 0.0373, Figure 4C), while prostaglandin D2 release was unaffected by 24 h cytokine priming following 1 h anti‐IgE stimulation (Figure 4D).

Given that IL‐33 induces CD32a expression in vitro, which is involved in the response to IgG immunocomplexes, we assessed the changes in MC activation in the presence/absence of IL‐33 and IgG. MCs respond weakly to direct activation with IgG2a‐containing immunocomplexes (< 10% CD63+ MCs, data not shown), we observed a significant boosting effect in FcεRIα‐mediated MC activation in the presence of IL‐33 and mixed IgG/IgG2a immunocomplexes (+28.8% CD63+ MCs, *p* = 0.0048, Figure S7C). This suggests that IL‐33 pre‐incubation primes MCs for mixed IgE/IgG responses.

**FIGURE 5:**
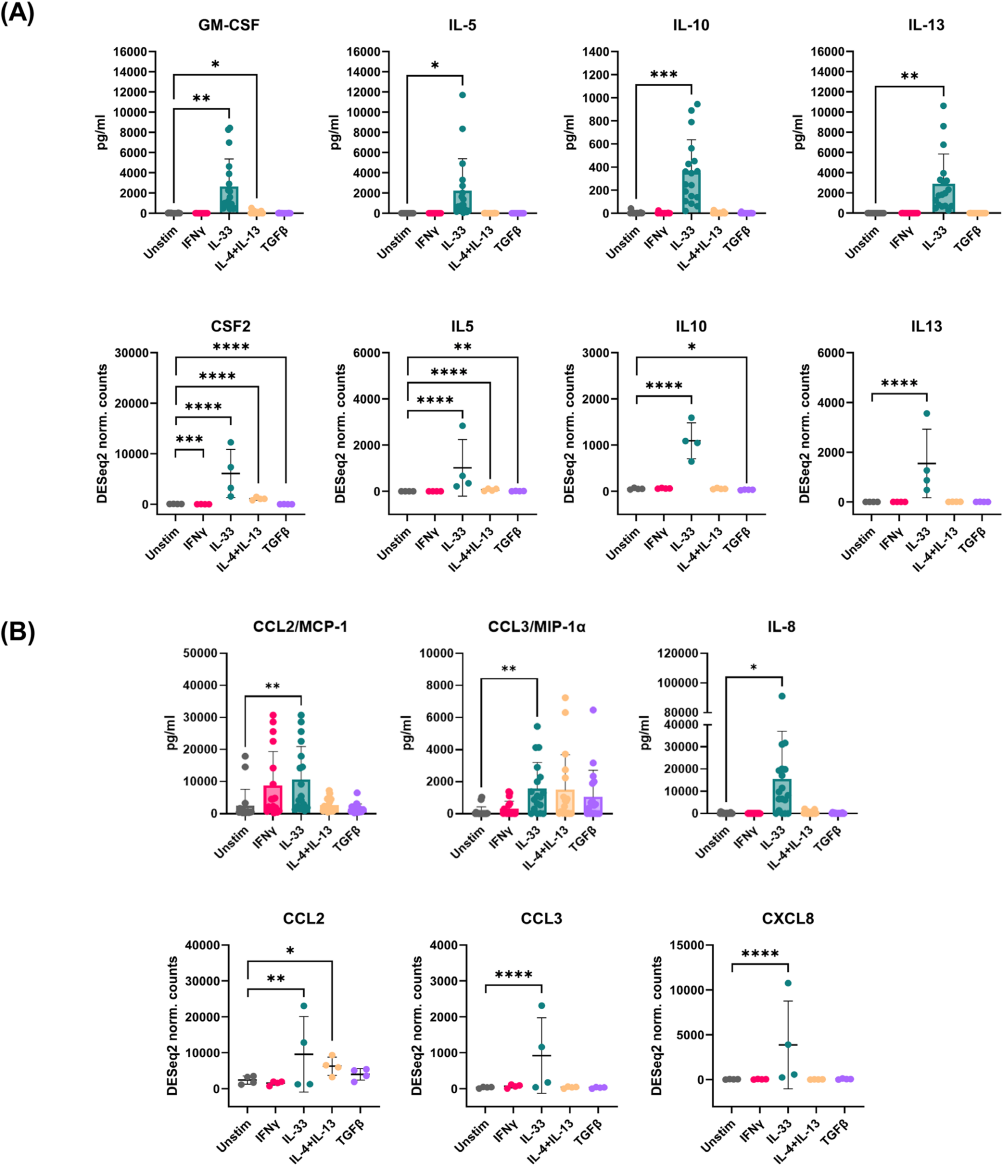
MC functional changes in response to cytokine treatment highlight the profound difference between IL‐33 and other stimulations. Primary human MCs were incubated with different cytokines for 24 h. Total mRNA was extracted for bulk sequencing (*n* = 4). Differential gene expression compared to unstimulated (adjusted by the false discovery rate method) and normalized counts were calculated using DESeq2's median of ratios approach. Cytokine (A) and chemokine (B) release on culture supernatants harvested at the end of 24‐h incubation with IFNγ, IL‐33, IL‐4 + IL‐13 and TGFβ was measured using cytometric bead array (CBA) (*n* = 14–18, top row) and compared to the transcriptomics findings (bottom row). Repeated measures one‐way ANOVA with Dunnett's correction was used for the statistical comparisons between different cytokines and the unstimulated control for the CBA assay. For ANOVA, significance was set at *p* < 0.05, while for multiple comparisons adjusted *p* values < 0.05 were considered significant. Data presented as mean ± standard deviation. Adjusted *p < 0.05, **p < 0.01, ***p < 0.001, ****p < 0.0001.

To further characterize the functional changes in response to different cytokines, we primed MCs with IFNγ, IL‐33, IL‐4 + IL‐13, and TGFβ for 24 h and measured cytokine/chemokine release in culture supernatants. IL‐33 was the only cytokine consistently driving the release of GM‐CSF, IL‐5, IL‐10, and IL‐13. Conversely, IL‐4 + IL‐13 only significantly increased GM‐CSF production, while the remaining cytokines did not influence the release of any of the investigated cytokines within the 24 h time frame, which aligns with the transcriptomics findings (Figure 5A).

Chemokine production was also boosted significantly by IL‐33 exposure, with increased spontaneous release of CCL2/MCP‐1, CCL3/MIP‐1α and IL‐8 (Figure 5B). Similarly, only IL‐4 + IL‐13 exposure increased CCL2/MCP‐1 release compared to the other cytokines, although at a lower level compared to IL‐33.

We conclude that IL‐33 and IL‐4 + IL‐13 skew MCs towards a cytokine/chemokine‐producing phenotype compared to IFNγ and TGFβ, which prominently act as modulators of MC IgE‐mediated activation.

### 3.4 Minimal Cytokine Gene Signatures Help Deconvolute Mast Cell Plasticity in Skin and Respiratory Diseases

To investigate whether MCs acquire cytokine‐activated signatures as a result of disease‐specific changes to the microenvironment, we used feature selection to identify key genes that retain the same information as the complete cytokine‐activated signatures. We reduced the full signatures to 281 highly informative genes (unstimulated = 36, IFNγ = 69, IL‐33 = 78, IL‐4 + IL‐13 = 26, TGFβ = 72 genes; Figure S8A–E, Table S3) with an accuracy (number of correct classifications divided by number of cases, Acc) and kappa (agreement between classification and true values) of 1 (95% CI: 0.4782–1, *p* value Acc >No Information Rate [NIR] = 0.00032).

A second validation by training a neural network model using the minimal signatures yielded an accuracy of 0.9, kappa 0.875 (*p* value Acc >NIR = 4.198e‐06), a multiclass area under the curve of 0.975, with the lowest performing signature being the unstimulated one, which was removed from subsequent analyses (AUC 1‐versus‐all = 0.75, Figure S8F).

The 245 identified genes were utilized to conduct GSVA on bulk and single‐cell transcriptomics datasets; 3 were used to validate individual cytokine signatures (Figure S9) [40, 41, 42, 43] and 3 for testing signatures in skin and respiratory diseases [29, 30, 31]. Using GSVA, in the Liu et al. skin dataset, we detected a positive enrichment of IL‐4 + IL‐13 signatures in AD and psoriasis compared to healthy controls.

A second dataset from West et al. [31] was used to analyze isolated MCs from active psoriatic lesions, and, contrary to what was observed in the Liu et al. dataset, we detected positive enrichment of IFNγ (*p* = 0.0359, FDR adjusted *p* = 0.072) and reduced IL‐4 + IL‐13 signatures (*p* = 0.0358, FDR adjusted *p* = 0.072, Figure S10) compared to healthy subjects, albeit only in unadjusted analyses.

**FIGURE 6:**
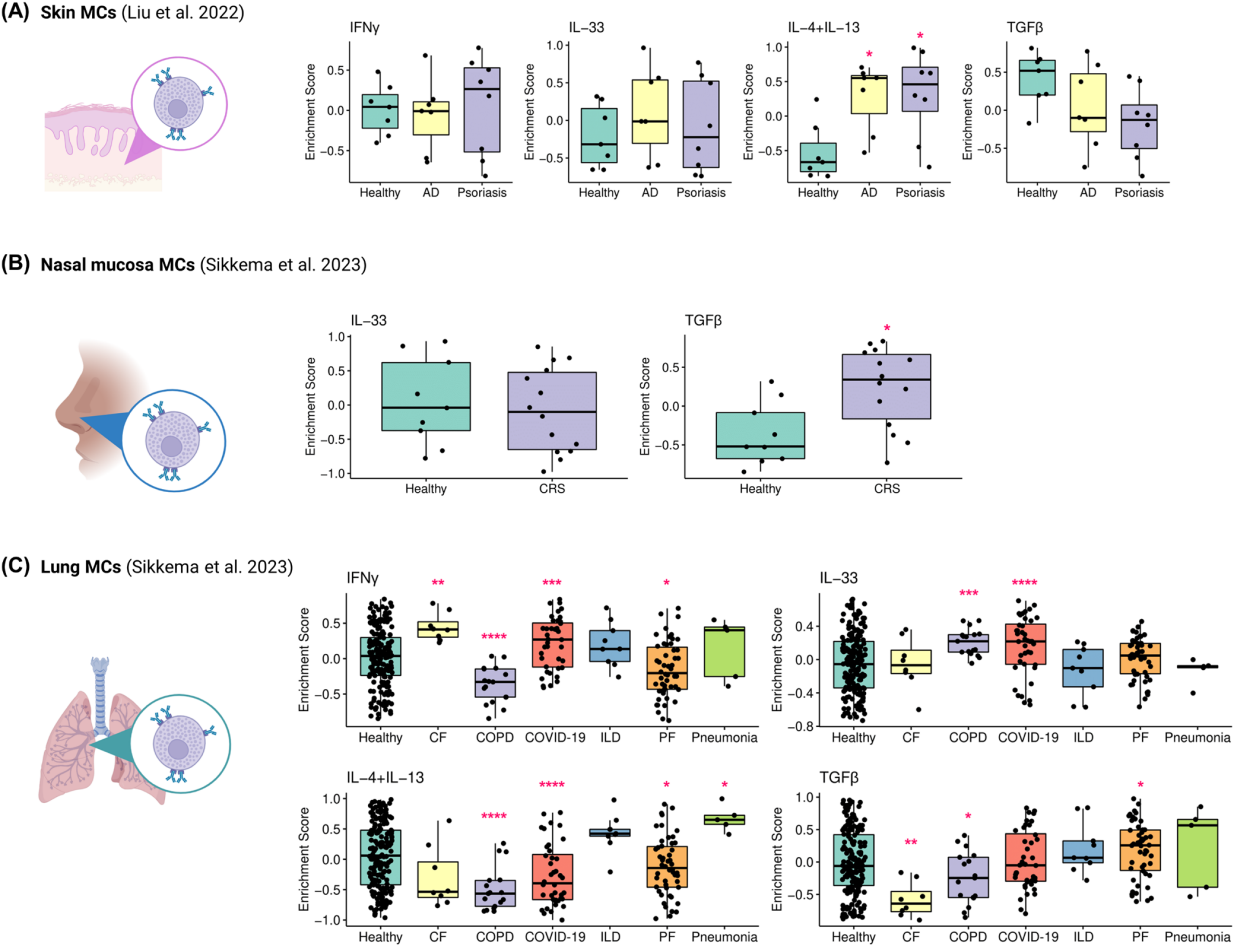
Cytokine‐activated MC signatures are enriched in different skin and respiratory diseases. Minimal cytokine‐activated signatures from uniquely expressed genes by single stimulations were selected using Elastic Net. Gene set variation analysis was calculated for each cytokine signature using tissue MCs pseudobulk counts obtained from the reanalysis of publicly available single‐cell transcriptomics datasets of skin diseases (atopic dermatitis, psoriasis; A) [30], upper respiratory (chronic rhinosinusitis; B) and lower respiratory diseases (cystic fibrosis, COVID‐19 infection, chronic obstructive pulmonary disease, interstitial lung disease, pulmonary fibrosis, pneumonia; C) [29] and plotted using ggplot2. Icons created in Biorender.com. Data presented as mean ± standard deviation. False discovery rate adjusted *p < 0.05, **p < 0.01, ***p < 0.001, ****p < 0.0001. AD, atopic dermatitis; CF, cystic fibrosis; CRS, chronic rhinosinusitis; ILD, interstitial lung disease; PF, pulmonary fibrosis.

In the Sikkema et al. respiratory disease dataset, we observed significant enrichment of IFNγ signatures in cystic fibrosis (CF) and COVID‐19 infection, IL‐33 in COPD and COVID‐19, IL‐4 + IL‐13 in pneumonia, and TGFβ signatures in pulmonary fibrosis (PF) and chronic rhinosinusitis (CRS) (Figure 6).

These findings suggest that MCs, under the influence of the microenvironment, acquire specific disease‐driven cytokine‐activated gene signatures, thus indicating different MC contributions in disease pathogenesis due to their plasticity, with potential therapeutic implications.

### 3.5 Tissue Mast Cell Clusters Exist in a Pseudotemporal Continuum and Adopt Disease‐Specific Cytokine Activation Trajectories

To further explore tissue MC plasticity and elucidate the influence of the surrounding microenvironment on MC clusters in disease, we used the minimal cytokine‐activated signatures to dissect cytokine priming at the single‐cell level.

**FIGURE 7:**
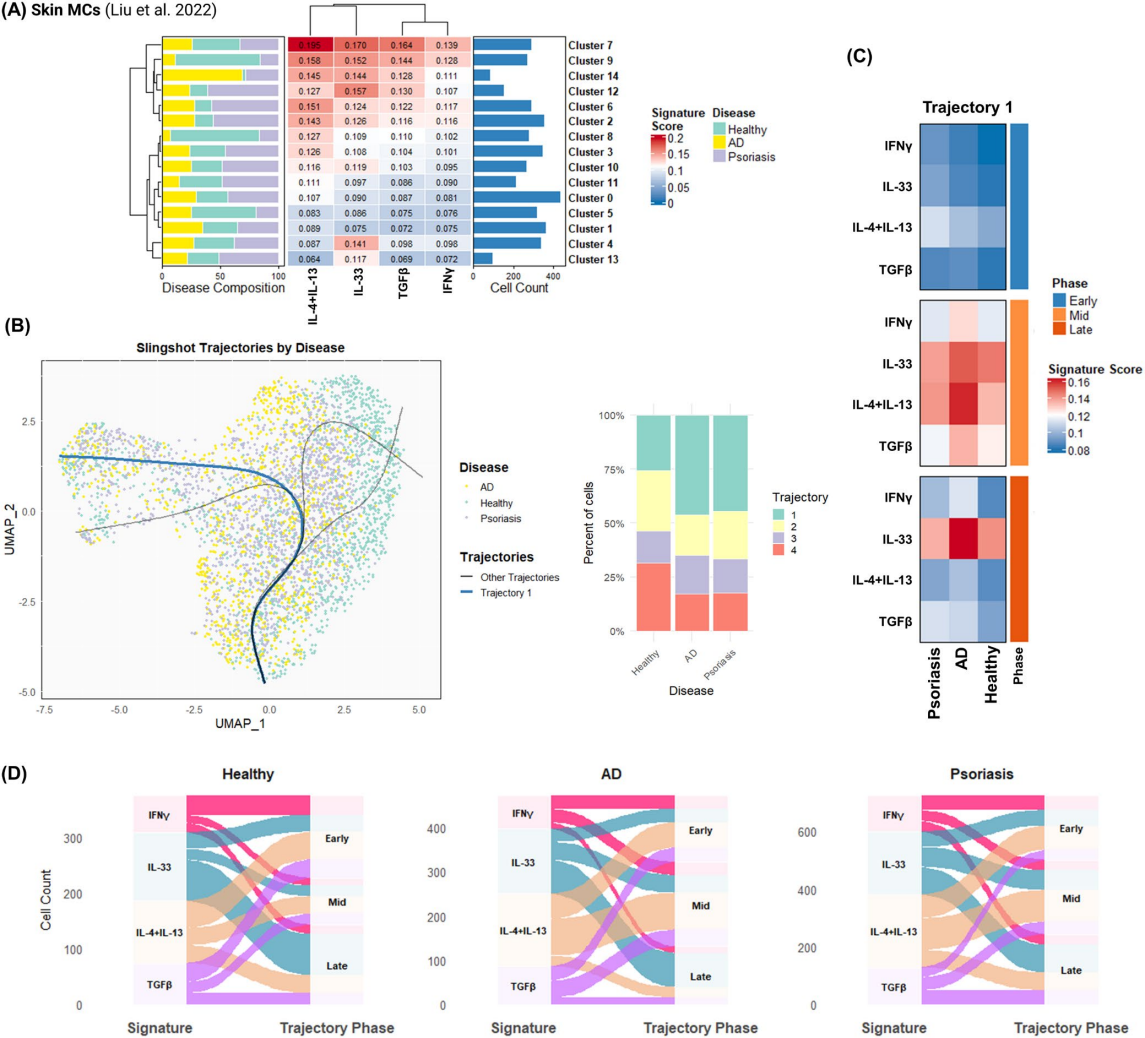
Skin MCs in atopic dermatitis and psoriasis are exposed to a mixed T1/T2 pro‐inflammatory microenvironment. MC clusters from the Liu et al. single‐cell dataset were extracted and re‐analyzed using the R package Seurat. (A) Cluster signature enrichment analysis was conducted using the minimal cytokine‐activated signatures and plotted using the R package ComplexHeatmap [44]. (B) Pseudotime trajectories were calculated using Slingshot, and the relative utilization per disease was compared to healthy MCs using Fisher's exact test. (C) Heatmap of signature scores per trajectory following natural binning and tertile phase grouping (early‐mid‐late) was plotted using the R package ComplexHeatmap. (D) Alluvial plots showing the flow of cells along pseudotime‐defined tertile phases were plotted using ggalluvial [45].

**FIGURE 8:**
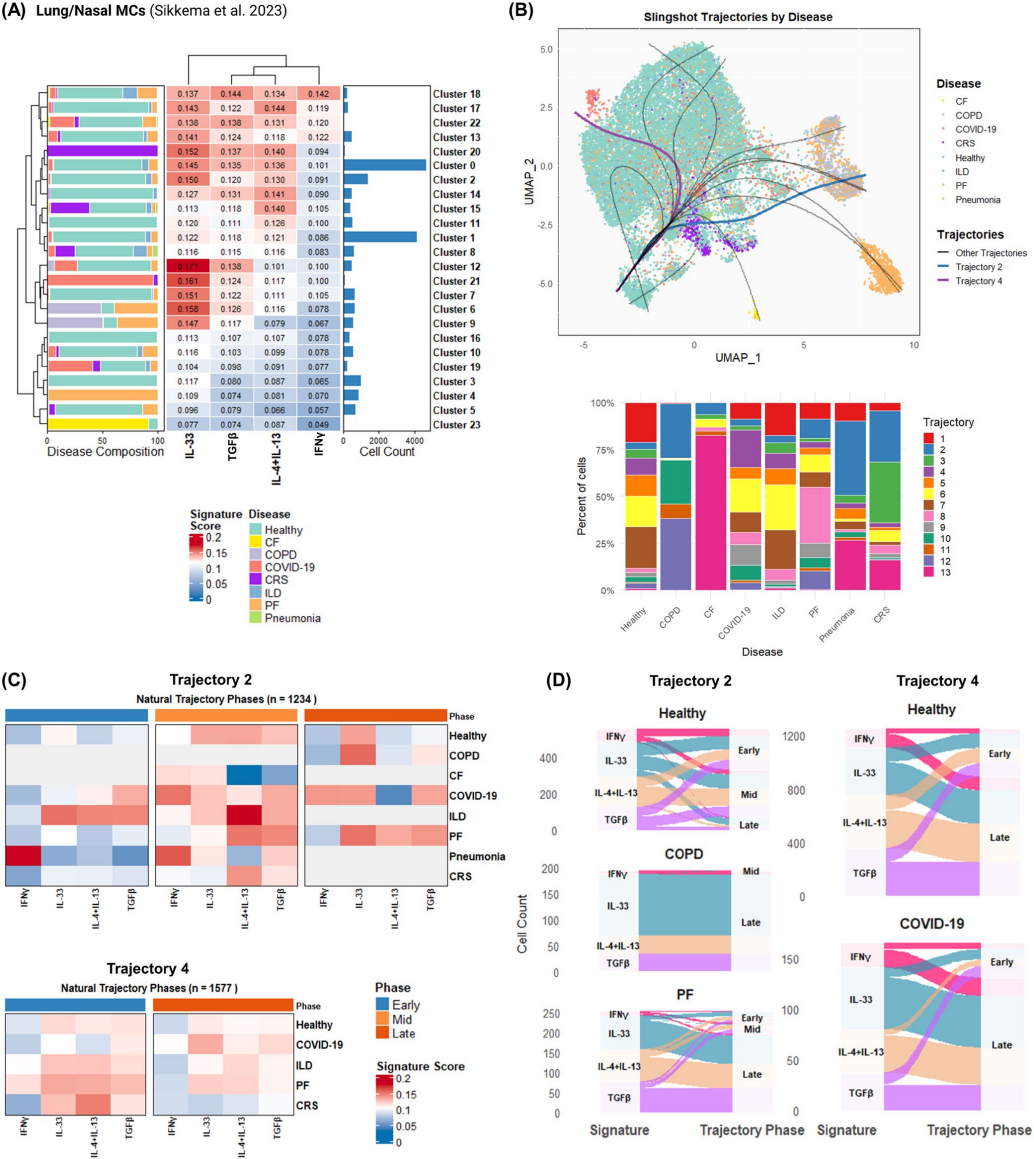
Nasal/lung MCs in COPD and pulmonary fibrosis adopt preferential pro‐fibrotic trajectories, while in COVID‐19 late mixed T1/alarmin‐driven responses are observed. MC clusters from the Sikkema et al. single‐cell dataset were extracted and re‐analyzed using the R package Seurat. (A) Cluster signature enrichment analysis was conducted using the minimal cytokine‐activated signatures and plotted using the R package ComplexHeatmap. (B) Pseudotime trajectories were calculated using Slingshot, and the relative utilization per disease was compared to healthy MCs using Fisher's exact test. (C) Heatmap of signature scores per significant trajectories following natural binning and tertile phase grouping (early‐mid‐late) was plotted using the R package ComplexHeatmap. Gray boxes indicate diseases with less than 5 cells per phase. (D) Alluvial plots showing the flow of cells along pseudotime‐defined tertile phases per each disease‐relevant trajectory were plotted using ggalluvial [45].

Rather than discrete cytokine‐specific populations, single‐cell analysis of MCs revealed distinct functional states organized along pseudotime trajectories and phases in both skin and lung tissue contexts (Figures 7 and 8 and Figures S11–S13).

In the Liu et al. skin dataset comprising 4097 MCs across 15 clusters, four main MC pseudotime trajectories were found (Figure 7A,B). In particular, Trajectory 1, utilized predominantly by AD and psoriasis, displayed significant overall upregulation of IFNγ (AD log_2_FC 0.286, FDR adjusted *p* = 2.7e‐06; psoriasis log_2_FC 0.248, FDR adjusted *p* = 2.7e‐06) and IL‐4 + IL‐13 signatures (AD log_2_FC 0.288, FDR adjusted *p* = 4.0e‐04; psoriasis log_2_FC 0.281, FDR adjusted *p* = 2.5e‐04), alongside TGFβ (AD log_2_FC 0.23, FDR adjusted *p* = 3.7e‐05; psoriasis log_2_FC 0.152, FDR adjusted *p* = 2.1e‐03) compared to healthy subjects, suggesting mixed T1–T2 and anti‐inflammatory responses in the dermis of affected patients (Figure S11). AD MCs in Trajectory 1 also showed significant IL‐33 upregulation, more prominent in late pseudotime (log_2_FC 0.17, FDR adjusted *p* = 3.4e‐03, Figure 7C, Figure S11D).

In the Sikkema et al. nasal/lung dataset, encompassing 19,388 MCs across 24 clusters organized into 13 trajectories (Figure 8A,B), two pathways (Trajectory 2 and 4) displayed overall significant cytokine signature enrichment with diverging biological programs compared to healthy MCs (Figure 8C,D, Figures S12 and S13). Trajectory 2 showed significant IL‐33 alarmin upregulation in COPD (log_2_FC 0.331, FDR adjusted *p* = 5.6e‐10) and elevation of multiple fibrotic signatures in pulmonary fibrosis, including TGFβ (log_2_FC 0.295, FDR adjusted *p* = 1.4e‐07), IL‐33 (log_2_FC 0.195, FDR adjusted *p* = 1.9e‐04), and IL‐4 + IL‐13 (log_2_FC 0.266, FDR adjusted *p* = 2.9e‐03) (Figure S13A). The terminal cluster of Trajectory 2, Cluster 6 (31% of trajectory cells, Figure S12C), displayed significant upregulation of PPARD (log_2_FC 3.57, FDR adjusted *p* = 2.83E‐81), a marker of metabolic reprogramming, ITGAX (log_2_FC = 1.26, FDR adjusted *p* = 5.48E‐11), and increased REL/NF‐κB signaling (log_2_FC 1.31, FDR adjusted *p* = 4.69E‐30, Figure S14). Conversely, Trajectory 4 captured late T1/alarmin‐driven inflammatory processes utilized by COVID‐19, demonstrating significantly increased IFNγ (log_2_FC 0.287, FDR adjusted *p* = 2.6e‐03) and IL‐33 responses (log_2_FC 0.218, FDR adjusted *p* = 4.6e‐03) compared to healthy MCs in late pseudotime phases (Figure S13B).

In conclusion, the single‐cell analyses offer new insights into MC plasticity, demonstrating that tissue MCs exist along activation continua rather than discrete cytokine‐specific populations. Furthermore, minimal cytokine‐activated signatures revealed distinct MC adaptation strategies associated with chronic inflammation in AD, psoriasis, COPD, COVID‐19, and PF.

### 3.6 IL‐33‐ and TGFβ‐Driven Signatures in Bronchial Brushings Hint at a Possible Mast Cell Role in Driving Eosinophil‐High COPD

To elucidate the role of cytokine‐activated MC signatures in identifying potential disease subsets in MC‐driven diseases, we investigated the influence of cytokines on different COPD endotypes. We measured gene expression scores of found signatures in previously published bulk transcriptomics of bronchial brushings from COPD subjects, with or without associated blood eosinophilia [37, 46, 47].

We detected increased and highly correlated expression of IL‐33 and TGFβ signatures in eosinophil‐high COPD (Figure S15A,B), while previously published signatures failed to pick up a positive signal [48, 49]. Interestingly, complete concordance with currently available IL‐33‐primed MC signatures [17, 48, 49] was limited to only 4 genes (CD70, IL13, KLF5, TNIP1) (Figure S16, Table S4). Using immunofluorescence, areas of localized IL‐33 production associated with active epithelial remodeling in COPD correlated with increased IL‐13+ MCs and extracellular IL‐13 release, which is not equally observed in IL‐33‐negative areas, suggesting IL‐13 as a marker for focal IL‐33 MC priming (Figure S15C).

Overall, these results hint at a possible role of MCs, and specifically IL‐33/TGFβ priming, in the pathophysiology of eosinophilic/T2‐high COPD.

## 4 Discussion

To our knowledge, this is the first study to identify unique and shared genes involved in cytokine response by human MCs, offering insights into cytokine‐activated MC plasticity in different tissues/disease microenvironments.

We provided shared genetic targets of cytokine modulation, with a peculiar focus on the FcεRI pathway, and means to identify MC priming by IFNγ, IL‐33, IL‐4 + IL‐13, and TGFβ in tissues and at the single‐cell level using machine learning‐defined minimal cytokine‐activated signatures.

Our transcriptomics, surface analyses, and functional results largely overlap with published evidence [50, 51, 52, 53, 54, 55, 56, 57], suggesting that primary MCs, although not identical to tissue MCs [9], are a reliable study tool to elucidate key pathways involved in cytokine response in humans.

In our shared gene analysis, 781 genes participated in multiple distinct signaling pathways, among which 15 were actively involved in FcεRI‐mediated responses. As genes such as FCER1A, early signaling molecules, and kinases were found highly modulated across different cytokines, this suggests a key positioning of these proteins in FcεRI‐mediated responses [58]. However, members of the LAT complex and PI3Ks are also involved in broader cell functions in immune (i.e., T cells, NK cells, granulocytes) and non‐immune cell types [59, 60]. Thus, therapeutically targeting these highly shared proteins without producing off‐target effects presents a significant challenge. For this, newer approaches aimed at selectively eradicating MCs are currently under investigation for skin and respiratory diseases [61, 62, 63].

Our surface receptor analysis confirmed at large the transcriptomics findings, except for MRGPRX2 due to its high expression variability. Such heterogeneity is donor‐dependent, as it is equally observed across multiple primary human MC generation protocols [64, 65]. However, evidence from skin MCs confirms that chronic exposure to IL‐33 has an inhibitory effect on MRGPRX2 expression [66], and recent evidence suggests that CXCL17, a chemokine released by multiple pro‐inflammatory signals, including IFNγ [67], can activate MCs through the MRGPRX2 receptor [68]. Among all investigated markers, HLA‐DR is the only molecule not expressed at steady state by mature MCs in vitro [69, 70] and upregulated by IFNγ priming [71, 72]. However, there is evidence of regional differences in HLA‐DR expression, particularly in lung MCs [73], and other stimuli induce MHC class II molecules expression [66], therefore their use as phenotypic IFNγ‐driven markers is limited.

As for the other investigated markers, since cytokine exposure only boosts their baseline expression, these cannot be used to predict cytokine priming from a purely phenotypic perspective. However, these findings open up avenues for further exploration of their role in MCs, particularly the role of integrin αvβ3 modulation by TGFβ in MCs, other than anchoring to vitronectin [74].

In our functional assay we observed opposing effects of IFNγ and TGFβ exposure on IgE‐mediated MC activation compared to unstimulated, consistent with existing literature [50, 75]. Conversely, we found no significant changes in CD63/CD107a expression following IL‐33 or IL‐4 + IL‐13 incubation. However, IL‐33 produces biphasic responses based on the duration of stimulation [52, 55], while IL‐4 + IL‐13 requires more than 24 h to produce a meaningful increase in MC activation [51]. Furthermore, new evidence of modulation of degranulation by IL‐33 has recently emerged, suggesting avenues for MC activation by IL‐33 beyond conventional IgE‐mediated pathways [76, 77]. Histamine release paralleled the MAT findings in IFNγ, while TGFβ priming displayed a trend towards histamine reduction, consistent with the literature [78]. Conversely, prostaglandin D2 release following IgE‐mediated activation was not significantly affected by cytokine priming, as prolonged stimulation (> 1 h) might be required to observe significant modulation [52]. As for IL‐33, the boosting of cytokine production by MCs and IgG‐mediated responses is consistent with what has already been observed in experimental models and in disease, such as in rheumatoid arthritis [52, 54, 57].

Hence, while transcriptomics analysis of cytokine‐activated MCs offered a broad scope of the gene expression changes induced by different cytokine microenvironments, we acknowledge that a perfect correlation with protein‐level changes, specifically the modulation of surface receptor expression and cytokine/chemokine release, was not always consistently observed. This discrepancy can be attributed to several known biological mechanisms and technical limitations. For instance, mRNA levels can be uncoupled from protein levels due to post‐transcriptional/translational regulation mechanisms [79]. Furthermore, the 24 h cut‐off was arbitrary and could be insufficient to observe significant protein‐level changes, as particularly evident with IL‐4 + IL‐13 stimulation [51]. In addition, there is known inherent biological heterogeneity among blood‐derived circulating MC progenitors [12], which could also contribute to the observed inconsistencies. These limitations are further compounded by the known MC differences in vitro and in vivo. Tissue MCs from the lung and gastrointestinal mucosa exhibit distinct constitutive gene and protein expression, such as IL‐5, IL‐13, or HLA‐DR expression compared to primary MCs [73, 80, 81, 82]. Therefore, our findings underscore that the local microenvironment plays a critical role in dictating MC function, and these specific microenvironmental cues are intentionally absent in our simplified in vitro system.

To deconvolute cytokine‐driven MC plasticity, we used machine learning and GSVA on tissue MCs from different skin/respiratory diseases. While our analyses confirmed at large the key role of cytokines in the pathogenesis of specific skin and lung diseases [83, 84, 85, 86], two exceptions were found.

In psoriasis patients, we expected to see primarily IFNγ signatures rather than IL‐4 + IL‐13. While IFNγ and the IL‐17/IL‐23 axis are well established in psoriasis [87], IL‐4 has been shown to downregulate IL‐1β and IL‐6 production by psoriatic cells and may aid in psoriasis resolution, as observed during treatment with vitamin D3 analogues [88]. Furthermore, new‐onset psoriasis can occur during anti‐IL‐4 receptor alpha treatment (dupilumab), suggesting a suppressive role mediated by the IL‐4/IL‐13 axis [89]. The Liu et al. dataset included subjects with psoriasis off topical and systemic steroids, but no information was provided about vitamin D3 analogue treatments.

To explore this discrepancy, we re‐analyzed a transcriptomics dataset in our possession [31]. Despite being underpowered, as 4 out of 6 psoriasis subjects displayed positive enrichment scores for IFNγ in unadjusted analyses, we believe IFNγ production could be highly donor specific and/or disease activity‐dependent.

As for the GSVA analysis of CRS, we could not compute IFNγ and IL‐4 + IL‐13 signatures due to the low number of expressed genes, despite analyzing a sufficient number of single cell MCs in both healthy and CRS sets (*n* = 193 and *n* = 508) and the similar frequency of zero‐read counts (74.8% vs. 76.8%). This contradicts the findings of Ordovas‐Montanes et al. [90], which reported increased IL‐13 and AREG expression in CRS compared to healthy MCs (hinting at IL‐33 priming), and increased representation of IFNγ and IL‐4 + IL‐13 signatures in secretory cells. However, in the integrated dataset by Sikkema et al. [29], only one healthy subject from the original publication was retained, which may have affected reproducibility. Nevertheless, TGFβ signatures were found enriched in CRS, consistent with other evidence from CRS with nasal polyps, where TGFβ contributes to epithelial‐mesenchymal transition, polyp growth, and tissue remodeling [91, 92].

To our knowledge, this study represents the first systematic attempt to deconvolute cytokine‐activated MC plasticity in human tissue at the single‐cell level. Our approach enabled the identification of distinct MC functional states across different diseases and the definition of MC clusters and trajectories associated with cytokine exposure. In particular, it provided evidence of preferential MC trajectories in diseases such as COPD/PF, dominated by late alarmin/pro‐fibrotic responses, and IFNγ/IL‐33 inflammation in COVID‐19, which further corroborate and expand our knowledge of the pathogenic mechanisms involving MCs in disease [47, 93].

The detection of coherent activation patterns, despite the inherent challenges including tissue complexity, signal‐to‐noise limitations, and spatially/temporally restricted cytokine activation, demonstrates the utility of cytokine‐activated signatures as tools for reducing biological complexity in vivo.

While pseudotime trajectory analyses broadly corroborated GSVA findings, the observed trajectories may also reflect spatial organization or technical variation rather than true temporal progression. Moreover, the known capacity for extended cytokine exposure to drive the acquisition of unique MC phenotypes, as recently demonstrated with prolonged TGFβ exposure in nasal polyps [94], likely contributes to the tissue‐specific transcriptomic and proteomic differences observed in MCs in vivo [9, 95]. For this, inducible pluripotent stem cell MC models could become useful assets to study specific cytokine‐driven developmental trajectories [96]. Therefore, while these findings establish a foundation for understanding MC functional heterogeneity, validation through larger ad hoc studies remains essential.

By excluding redundant genes across multiple stimulations, we aimed to improve the detection of cytokine‐activated MC signatures by minimizing background noise. Unlike in Badi et al. [17], where nearly all upregulated genes were used for GSVA (*n* = 418), our approach allowed us to detect IL‐33 priming using only 78 informative genes. Supported by the re‐analysis of the COPD dataset by Higham et al. [37], our IL‐33 MC signature outperformed the ones by Tiotiu et al. [48] and Nagarkar et al. [49], and showed complete concordance with other available signatures [17] in only 4 genes, which included IL13. Furthermore, we found that IL‐13 is preferentially released in areas of focal epithelial remodeling associated with IL‐33 production. This evidence underscores the critical role of IL‐33‐activated MC in the cross‐talk with damaged epithelia, significantly contributing to local epithelial dysfunction [97, 98]. As IL‐33 stimulation also results in the production of GM‐CSF, IL‐5, and IL‐8 by MCs, which are crucial for eosinophil maturation and migration [99, 100], said evidence supports the use of dupilumab and/or anti‐IL‐33 antibodies for treating eosinophil‐high COPD by possibly blocking MC‐driven contribution to local eosinophil recruitment. Notably, phase II‐III results of itepekimab, an anti‐IL‐33 monoclonal antibody, and dupilumab showed improvement in lung function in this particular COPD subset [101, 102]. Interestingly, the correlation between eosinophil‐mediated diseases and IL‐33/TGFβ co‐priming was also observed in one validation set involving eosinophilic esophagitis MCs (Figure S9B) [40, 41]. Although not fully demonstrated on MCs, this could be the effect of IL‐33 exposure mediated by other effector cells, as both eosinophils and macrophages exposed to IL‐33 release TGFβ via NF‐κB signaling [103, 104].

While our approach offers several advantages, both single‐cell analyses and GSVA are sensitive to zero‐read artifacts that could fail to detect significant cytokine signatures, which are dependent on the quality of MC isolation from tissues.

Furthermore, our signatures do not account for the influence of MC activation, as cytokines released by MCs can still produce autologous stimulation. In our hands, MC IgE‐mediated activation using the major cat allergen Fel d 1 (as described by Le Floc'h et al.) [42] especially affected IL‐4 + IL‐13 and TGFβ signatures (Figure S9A). Therefore, the results of cytokine enrichment alone should be interpreted with caution, especially in conditions where MC activation is highly likely (e.g., allergic diseases, chronic spontaneous urticaria). Ideally, these results should be complemented with signatures designed to detect IgE‐mediated MC activation [48, 105, 106].

## 5 Conclusions

In conclusion, we have demonstrated a feasible approach to understand cytokine‐driven MC plasticity by combining transcriptomics, functional assays, and machine learning, and using primary human MCs as an investigative platform. Our findings reveal shared and unique signatures that could lead to more targeted microenvironment‐oriented strategies to modulate MC activation, providing both potential druggable targets and a tool to dissect MC cytokine priming in disease. Furthermore, minimal signatures may serve as biomarkers to guide treatment decisions with anti‐cytokine biologics and for monitoring treatment efficacy in MC‐mediated diseases.

## Author Contributions

C.T., A.S., R.B., and S.B.P. designed the study. C.T., A.H., and R.B. performed the experiments. C.T., R.B., and A.H. analyzed the data and prepared the figures/tables. C.T., R.B., A.H., D.S., A.S., and S.B.P. wrote the manuscript.

## Conflicts of Interest

Dave Singh has received sponsorship to attend and speak at international meetings, honoraria for lecturing or attending advisory boards from the following companies: Adovate, Aerogen, Almirall, Apogee, Arrowhead, AstraZeneca, Bial, Boehringer Ingelheim, Chiesi, Cipla, CONNECT Biopharm, Covis, CSL Behring, DevPro Biopharma LCC, Elpen, Empirico, EpiEndo, Genentech, Generate Biomedicines, GlaxoSmithKline, Glenmark, Kamada, Kinaset Therapeutics, Kymera, Menarini, MicroA, OM Pharma, Orion, Pieris Pharmaceuticals, Pulmatrix, Revolo, Roivant Sciences, Sanofi, Synairgen, Tetherex, Teva, Theravance Biopharma, Upstream, and Verona Pharma. All other authors have no conflicts of interest to disclose for the present manuscript.

## Supporting information


**Appendix S1:** all70052‐sup‐0001‐AppendixS1.docx.

**Appendix S2:** all70052‐sup‐0002‐AppendixS2.pdf.

**Data S1:** all70052‐sup‐0003‐DataS1.xlsx.

## Data Availability

The data that support the findings of this study are available from the corresponding author upon reasonable request.
