# Supplementary material for: Microenvironment‐Driven Mast Cell Plasticity: Insights From Cytokine‐Activated Gene Signatures in Skin and Respiratory Diseases
Source: Allergy. 2025 Sep 10;80(11):3077–94. doi: 10.1111/all.70052 (PMC12590347; doi:10.1111/all.70052)
Supplement: Supplementary file 1 — Appendix S1: all70052‐sup‐0001‐AppendixS1.docx. [file ALL-80-3077-s003.docx]

**Supplemental information Tontini et al.**

1. **Materials and methods**
   1. ***Generation of primary human MCs***

Isolation of circulating CD117+ MC progenitors was performed on whole blood collected from anonymous donors by the NHS Blood and Transplant Unit after signing an informed consent (UREC ref. 2018-2696-5711) as previously described by Bahri & Bulfone-Paus(1). Human peripheral blood mononuclear cells (PBMCs) were isolated from other blood components using density gradient separation (Ficoll-Paque, GE healthcare). To isolate CD117+ MC precursors, CD117 microbeads and Fc receptor blocker (CD117 microbead kit, Miltenyi Biotec) were added to PBMCs at the concentration suggested by the manufacturer, and incubated for 30 minutes in the dark at 4°C. Cells were then centrifuged and loaded into magnetic LS columns (Miltenyi Biotec). Cells were then resuspended in culture media at a concentration of 10^6^ cells/ml and incubated at 37°C and 5% CO_2_ for cell culture.

During the first four weeks in culture, MC progenitors were cultured in media containing IMDM+GlutaMAX (Gibco), bovine serum albumin (BSA) Fraction V (Gibco) 0.5%, Insulin-transferrin (Gibco) 1%, β-Mercaptoethanol 50mM, Penicillin 100 U/ml (Sigma-Aldrich), Streptomycin 100 μg/ml (Sigma-Aldrich), recombinant human SCF 100 ng/ml (GenScript), recombinant human IL-6 (GenScript) 50 ng/ml and recombinant human IL-3 (Peprotech) 10 ng/ml. From week 5, the concentration of IL-3 was progressively reduced by supplementing fresh culture media without recombinant human IL-3, and by the end of week 6 culture media was completely replaced with media without IL-3. To assess functional maturation after week 8-10 of culture, MCs were aliquoted in triplicates of 50x10^4^ cells/well and sensitized for 16 hours with human myeloma IgE (CalBiochem) at 1 μg/ml concentration. Subsequently, MCs were either stimulated with anti-human IgE (KPL) at 1 μg/ml for 1 hour, or left unstimulated (negative control). Maturity was assessed using the expression of surface markers CD117 and FcεRI (clone 104D2 and AER-37, Biolegend) and/or via functional assessment of degranulation using CD63 (clone H5C6, Biolegend) and CD107a (clone H4A3, Biolegend) as markers by flow cytometry. Donors were selected if showing ≥90% CD117+FcεRI+ expression (**Supplemental Figure S2**) and/or showing upregulation of CD63/CD107a in anti-IgE stimulated samples in above 70% of gated live cells.

- 1. ***Cytokine priming***

Mature MCs were resuspended at a concentration of 10^6^ cells/ml and primed using culture media (unstimulated), IFNγ 50 ng/ml (Peprotech), IL-33 50 ng/ml (Peprotech), IL-4 plus IL-13 10 ng/ml (Peprotech) or TGFβ 5 ng/ml (Peprotech) for 24 hours. Cytokine concentrations were chosen based on either available published literature(2–5) or dose ranging experiments previously performed in our laboratory. After incubation, cells were harvested and culture supernatant stored at -20° C for future processing.

- 1. ***Bulk RNA sequencing of human MCs primed with cytokines***

For RNAseq experiments, MCs generated from 4 healthy donors were primed with cytokines for 24 hours in culture media devoid of IL-6. Following 24-hour incubation, cells were harvested, washed with sterile PBS 1 ml and centrifuged at 300 x g for 3 minutes. RNA from MCs was extracted using a commercially available RNA extraction kit (RNAeasy micro kit, Qiagen). Extraction steps were performed following the protocol supplied by the manufacturer. Quantification of extracted RNA and purity were measured via spectrophotometry (Nanodrop 2000, Thermo Fisher). Isolated RNA was refrigerated at -80° C and submitted within 24 hours to the University of Manchester Genomic Technologies Core Facility for next generation sequencing using HiSeq®4000 sequencer (Illumina).

- 1. ***Bioinformatics analysis pipeline and gene set enrichment***

Generated RNAseq FASTQ files were processed by the Bioinformatics Core Facility. Briefly, FASTQ files underwent quality control analysis using FastQC(6), and trimmed with BBDuk(7) when necessary. Trimmed FASTQ files were aligned to the human genome using the human hg38 reference and Gencode v36 annotation(8).

Raw counts for each sample in different experiments were produced using STAR v2.7.7a with 2-pass mapping using --quant mode GeneCounts function(9). Differential gene expression analysis was performed using the R package DESeq2 v1.30.1(10).

Differential gene expression analyses were carried out using the Wald test to compare unstimulated versus cytokine-treated samples with the design formula ~Donor+Condition for each dataset individually. Significance after Bonferroni-Hochberg false discovery rate (FDR) adjustment was set at <0.05. Raw counts were normalized using DESeq2’s median of ratios method. Principal component analysis was performed using variance stabilized transformed (VST) counts and plotted using the ggplot2 package(11). Heatmaps were generated using the package pheatmap(12), while Venn diagrams were generated using ggvenn(13).

For gene enrichment analyses, we selected either shared differentially expressed genes of interest, prefiltered using the FDR p adjusted value threshold of less than 0.05 (n=781) (**Figure 1A, Supplemental Table 1**), or unique upregulated (log2 fold change>1, FDR adjusted p<0.05) protein-coding genes per each cytokine (**Supplemental Figure S5A**). Enrichment analyses were carried out using Metascape(14), an open-source platform that performs multiple enrichment analyses using different ontology sources at once. We performed a custom analysis which included the Kyoto Encyclopedia of Genes and Genomes (KEGG)(15), Canonical Pathways, Gene Ontology (GO) biological processes(16), Reactome Gene Sets(17) and/or WikiPathways(18) (**Supplemental Figures S3B, S4B, S5C**). For the analysis of possible transcriptional regulators the Transcriptional Regulatory Relationships Unraveled by Sentence-based Text mining (TRRUST)(19) enrichment was conducted on upregulated unique genes only (**Supplemental Figure S5D**). All genes present in the human genome were used as enrichment background, as per Metascape default setting. Returned enriched terms were considered of interest if the log10 q value was ≤ -1.3 and were plotted using the ggplot2 package.

Protein-protein interaction analysis was also performed using Metascape on unique upregulated genes, selecting subsets of proteins forming known physical interactions with at least one other member in the provided gene list using STRING(20) (physical score>0.132). The Molecular Complex Detection (MCODE) algorithm(21) characterised densely connected network components, and one among the three top scored enrichment terms sorted by p value was used to describe each cluster of nodes. Networks were displayed using Cytoscape v3.9.1(22).

- 1. ***Surface receptor staining***

Following cytokine priming, MCs were aliquoted in duplicates per each condition (50x10^4^ cells/well) and washed using phosphate buffer saline (PBS). Cells from 3-7 donors were then incubated with Fc receptor blocking solution (1:100, Biolegend) and a live/dead marker (1:1000, Live/Dead Fixable Blue, Thermo Fisher Scientific) for 15 minutes on ice. Subsequently, cells were topped up in a 1:2 ratio with CD117 (clone 104D2, Biolegend), FcεRI (clone AER-37 or QA20A04, Biolegend), MRGPRX2 (clone K125H4, Biolegend), FcγRII/CD32 (clone FUN-2, Biolegend), FcγRIIb-c/CD32b-c (clone S18005H, Biolegend), HLA-DR (clone L243, Biolegend) and Integrin αvβ3 (CD51-CD61, clone 23C6, Biolegend) in MACS buffer (PBS, 0.5% BSA, 2 mM ethylenediaminetetraacetic acid, Invitrogen) at an end concentration of 1:200 and incubated for 20 minutes on ice in the dark. Compensation beads for mouse antibodies (BD Biosciences) and FMO controls (pooled 10 μl aliquots from each donor taken from both unstimulated and cytokine-primed MCs) were also stained for 20 minutes. Samples and FMO controls were subsequently fixed with paraformaldehyde 2% (Sigma Aldrich) on ice for 15 minutes, washed with MACS buffer and analysed in flow cytometry (BD Biosciences).

Flow cytometry data was analysed using FlowJo (BD Biosciences). Surface markers expression was measured on pre-gated live MCs using normalized geometric mean fluorescence intensity (intensity of the marker in measured samples divided by the intensity of the marker in an unstained control). FMO controls were used to gate marker positive MCs (gating strategy in **Supplemental Figure S6**).

- 1. ***Mast cell Activation Test (MAT)***

After 6 hours from the start of MC priming with cytokines, MCs (n=4) used for the MAT assay were sensitized using human myeloma IgE added directly to the 24-well plates at 100 ng/ml concentration and left overnight. At the end of 24-hour priming, MCs were harvested and plated in duplicates (50x10^4^ cells per well) in v bottom 96-well plates. Cells were then washed and either unstimulated (control) or stimulated with goat anti-human IgE (KPL) at 50 ng/ml concentration for 1 hour.

For the experiments involving IgG-FcεRI immunocomplex activation, washed IL-33-primed MCs or unstimulated controls (n=4) were pre-treated with human Fc blocking solution 1:100 for 10 minutes on ice. Direct activation of MCs was then produced by adding mouse anti-FcεRI antibody 1 μg/ml (clone AER-37, Biolegend), with or without concomitant addition of IgG2a kappa mouse antibodies 1 μg/ml (clone eBM2a, Biolegend). Cross-linking of anti-FcεRI and IgG antibodies to produce immunocomplexes was obtained by using an anti-mouse lambda chain antibody at 5 μg/ml concentration given shortly after the addition of anti-FcεRI/IgG2a, and MCs were stimulated for 1 hour at 37°C.

At the end of the stimulation, cells were washed in PBS and incubated with Fc receptor blocking solution 1:100 and live/dead marker 1:1000 for 15 minutes on ice. Subsequently, cells were topped up in a 1:2 ratio with CD63 and CD107a in MACS buffer (end concentration 1:200) and incubated for 20 minutes on ice in the dark. Compensation beads for mouse antibodies and FMO controls (pooled 10 μl aliquots from each donor taken from all experimental conditions) were also stained for 20 minutes. Samples and FMO controls were subsequently fixed with formaldehyde 2% on ice for 15 minutes, washed with MACS buffer and analysed in flow cytometry.

Flow cytometry data was analysed using FlowJo, and activation was measured as percent of live MCs expressing CD63 and CD107a using FMO controls (gating strategy in **Figure 4A**).

- 1. ***Histamine, prostaglandin D2, cytokine/chemokine release in cell culture supernatants***

To measure histamine and prostaglandin D2 release, 24-hour cytokine-primed MCs (n=5) were sensitized overnight with IgE 100 ng/ml washed and incubated with fresh media with or without anti-IgE 50 ng/ml stimulation for 1 hour. Cell culture supernatants per each experimental condition were harvested, and histamine and prostaglandin D2 release were measured in triplicates using commercially available ELISA kits following manufacturer’s instructions (Abnova, Caiman Chemicals).

To measure the release of the cytokines GM-CSF, IL-5, IL-10, IL-13 and chemokines IL-8, CCL2/MCP-1 and CCL3/MIP-1α in harvested cell culture supernatants (n=14-18), cytometric bead array was used following manufacturer’s instructions (BD Biosciences). Samples were then acquired on a flow cytometer and measured in FlowJo.

- 1. ***Immunofluorescence***

Peripheral lung tissue was obtained from a COPD patient undergoing surgical resection for suspected lung cancer. Lung tissue was sampled distal to the tumour and determined to be nodule free by a clinical pathologist. Written informed consent was obtained using protocols approved by local research ethics committees (03/SM/396 South Manchester Ethics Committee and 20/NW/0302 Northwest Ethics Committee).

Explanted lung tissue was inflated and fixed in 4% neutral buffered formalin, before being cut into tissue blocks, processed, and embedded in paraffin wax. The lung tissue block most distal to the tumour site was selected, cut into 4 μm sections, de-waxed in xylene and rehydrated in graded alcohols prior to antigen retrieval for immunofluorescence protocol. Antibody validation was performed to determine optimal antigen retrieval and antibody dilution conditions prior to staining the samples and specificity confirmed using isotype controls and the omission of the primary antibody. Heat induced epitope retrieval (HIER) was conducted for 20 minutes at 97°C using Epredia Dewax and HIER Buffers and Epredia Lab Vision PT Module (Fisher Scientific). Staining was performed sequentially using i) 1:800 anti-human Tryptase (clone AA1, Abcam), ii) 1:50 anti-human IL-13 (clone OTI6D3, Thermo Fisher) and iii) 1:100 IL-33 (clone Nessy-1, Enzo Life Sciences), following no antigen retrieval, antigen retrieval at pH 6 and pH 9, respectively. Following antigen retrieval, slides were washed using 1x Tris-Buffered Saline, 0.05% Tween 20 before incubating in protein block (Abcam) for 20 minutes. Slides were then incubated with the first primary antibody for 30 minutes at room temperature then overnight at 4°C. Slides were then brought to room temperature for 30 minutes prior to washing. Staining was then detected using the anti-mouse or anti-rabbit poly-HRP Alexa Fluor Tyramide SuperBoost kits (488nm Tryptase, 647nm IL-13, 546nm IL-33) as per manufacturer’s guidelines (Thermo Fisher). Following detection, slides were checked under the microscope to confirm presence of staining, before moving onto the next antibody protocol. Following the final antibody application, Hoescht 33258 was applied for nuclear counterstaining and slides were cover-slipped using Vectashield Antifade Mounting Medium (Vector laboratories, UK). Digital micrographs of complete membranous bronchioles (<2mm diameter) were acquired using the EVOS M7000 imaging system (Thermo Fisher).

- 1. ***Feature selection***

To narrow down the number of identified unique protein-coding genes to create minimal cytokine-activated gene signatures, we used variance stabilized counts to perform feature selection using the machine learning algorithm Elastic Net (R packages glmnet and caret)(23,24). Briefly, we selected 3873 significant protein-coding genes not shared across different cytokines from our bulk RNAseq dataset (IFNγ=1053, IL-33=1616, IL-4+IL-13=248, TGFβ=956 genes) and partitioned the dataset into training and testing sets (70/30). Optimal lambda values (lambda.min) were obtained using the cross-validation function cv.glmnet, with the final chosen model using an alpha of 0.5, lambda 0.007976564 and family “multinomial” as parameters. The model was then used to predict the likelihood of belonging to a specific cytokine group of the testing set, accuracy and class-specific performance indicators of the model were then calculated using the function confusionMatrix.

To further validate the minimal cytokine-activated unique signatures found and measure their retained accuracy in correctly identifying cells treated with the cytokines of interest, the identified 281 genes using Elastic Net (Unstimulated=36, IFNγ=69, IL-33=78, IL-4+IL-13=26, TGFβ=72 genes, **Supplemental Figure S8A-E**) were used to train a Neural Network model (package neuralnet)(25). The reduced dataset was partitioned into testing and training (50/50), the model trained using the function neuralnet and used to predict the cytokine class using the predict function. Accuracy, class-specific sensitivity and specificity of the model was then calculated using the function confusionMatrix on the testing set. Area under the curve of the multiclass analysis was obtained using the multiclass.roc function and one-versus-all AUC curves were plotted per each cytokine class using the package pROC(26) (**Supplementary Figure S8F**).

- 1. ***Re-analysis of single-cell RNAseq public datasets***

To validate and test our reduced signature and obtain information on MC priming in different diseases/conditions, publicly available single-cell RNAseq (scRNAseq) (Gene Expression Omnibus (GEO) GSE201153, GSE179633, GSE179633; Zenodo: <https://zenodo.org/deposit/5228495>; CellXGene: <https://cellxgene.cziscience.com/collections/6f6d381a-7701-4781-935c-db10d30de293>) or bulk RNAseq (GSE140900, GSE217060) datasets were re-analyzed by the Bioinformatics Core Facility of the University of Manchester.

scRNAseq datasets for which there was evidence of production of the cytokine of interest by neighbouring cells and/or MCs were used to validate the cytokine-activated signatures by tissue MCs.

The GSE201153 dataset(27,28) included esophageal biopsies of healthy subjects (n=2) and patients diagnosed with active Eosinophilic Esophagitis (EoE, n=5) and was selected to validate the IL-33 and TGFβ signatures.

The GSE179633 dataset(29) included skin of healthy subjects (n=2), and patients with discoid lupus erythematosus (DLE, n=5) or systemic lupus erythematosus (SLE, n=7) and was used to validate the IFNγ signature.

As none of the available scRNAseq datasets presented a clearly defined IL-4 and/or IL-13 signature, we opted for the re-analysis of a bulk RNAseq dataset, the GSE140900 dataset, which included CD133-derived human MCs from 4 different donors, differentiated *in vitro* using the protocol stated in *Le Floc’h et al.*(30). Primary MCs (n=4) were exposed to IL-4 or IL-13 at 100 nM concentration for 24 hours, with/without stimulation with the major cat allergen Fel d 1 at 1 nM concentration for 4 hours (following sensitization overnight with Fel d 1-specific IgE 10 nM), or left unstimulated. Total RNA was then extracted and sequenced using an HiSeq®2000 sequencer (Illumina). Raw FASTQ files were downloaded from GEO, aligned and re-analysed with the same bioinformatics pipeline described in section 1.4.

Once validated, chosen scRNAseq datasets for testing included the integrated Human Lung Cell Atlas(31), which consists of a collection of scRNAseq sets of respiratory airway/lung (n=95) and nasal mucosa (n=9) isolated from healthy subjects and patients with different nasal and lung diseases, including chronic rhinosinusitis (n=14), cystic fibrosis (n=8), chronic obstructive pulmonary disease (n=17), COVID-19 infection (n=40), interstitial lung disease (n=9), pulmonary fibrosis (n=50) and pneumonia (n=5) selected for the present analysis.

For skin diseases, the *Liu et al.* study(32) was used to analyse skin MCs in healthy (n=7), atopic dermatitis (n=7) and psoriasis (n=8) subjects.

For additional testing of the MC cytokine signature in psoriasis, we re-analysed the GSE217060 bulk RNAseq dataset in our possession using the pipeline described in section 1.4. The dataset included MCs isolated from skin of healthy subjects (n=7) and active lesions of psoriasis patients (n=6, 2 subjects pooled)(33).

All selected scRNAseq sets were analysed on 10x Genomics platforms (10x Genomics, Inc.). For the re-analysis, scRNAseq datasets were imported to R and MC clusters were retrieved based on the annotation provided by the original authors.

MC clusters were analysed using Seurat v5(34). Raw count data were normalized using the NormalizeData function with donor effects regressed out. Principal component analysis was performed on variance-stabilized counts of top 3000 variable features and clusters were identified using shared nearest neighbour graphs and Leiden algorithm via the function FindClusters. Clusters were visualized using Uniform Manifold Approximation and Projection (UMAP) for dimensionality reduction. Differential gene expression analysis was performed using the FindAllMarkers Seurat function to identify cluster-specific genes and only positive markers (log2 fold change > 0) with a minimum percentage threshold of 10%. Statistical testing was then performed using Wilcoxon rank sum test and adjusted using the FDR method.

- 1. ***Pseudotime cluster analysis of single cell data***

Pseudotime trajectory analysis was conducted using Slingshot v2.0(35) on UMAP embeddings, with trajectories inferred to identify progression paths between MC activation states. A natural binning approach was implemented using density peak detection in pseudotime distributions through histogram-based estimation (30 bins), local maxima detection with moving average smoothing, and bin boundaries established at midpoints between consecutive peaks to preserve biologically meaningful trajectory phases. Natural bins were then ordered along pseudotime and grouped into tertile-defined phases (Early, Mid, Late). Cells were assigned to trajectories using a winner-takes-all approach based on slingshot weights (>0.1 threshold), retaining only cells with valid pseudotime values. Disease-specific trajectory enrichment was assessed using Fisher's exact tests comparing the proportion of cells from each disease in each trajectory versus all other trajectories, with p-values adjusted for multiple testing using the FDR method. Single-cell cytokine pathway signature scores were calculated as the mean log-normalised expression of pathway-specific gene sets. For each trajectory and disease combination, cytokine pathway signatures were compared between disease and healthy cells using t-tests and significance assessed after FDR correction (p<0.05). Visualizations including trajectory progression analysis, alluvial diagrams, and cluster trajectory network plots were generated using ggplot2 and ggalluvial(36), while integrated heatmaps were created using ComplexHeatmap(37).

- 1. ***Extraction of pseudobulk tissue mast cell counts and gene set variation analysis (GSVA)***

To compute set-defined gene enrichment scores based on the minimal cytokine-activated signatures, we used the package GSVA to perform gene set variation analysis(38). Pseudobulk raw counts were generated from single-cell counts using the summarizeAssayByGroup function of the package scater(39), and normalized/log2 transformed using the calcNormFactors and cpm functions of the EdgeR package(40).

For all analysed bulk RNAseq datasets, normalized counts were extracted using the DESeq2 counts function and then log2 transformed. All normalized log2 counts were then filtered from lowly expressed genes using the edgeR filterByExpr function, except in GSE201153 where this step was skipped as the number of MCs analysed from healthy subjects was small (total 19 single MC events) and showed an average of 86.52% zero reads for the genome analysed.

To calculate differential gene expression between healthy and specific diseases of interest, a linear model was fit using limma’s lmFit function and moderated t-statistics were computed using the eBayes function(41). The gsva function of the GSVA v1.38 package was then used to retrieve enrichment scores based on the minimal cytokine-activated gene signatures defined using feature selection, and the same linear model/moderated t-statistics used to calculate differences between healthy and disease/conditions was applied to GSVA scores and adjusted for multiple comparisons using the Bonferroni-Hochberg FDR method. The final results were then plotted using the ggplot2 package.

- 1. ***Manual gene expression scores using minimal cytokine-activated signatures***

To further validate the found minimal cytokine-activated signatures by using methods other than GSVA, we calculated manual gene expression scores using a previously published bulk RNAseq dataset of bronchial brushings from n=20 eosinophil^high^ (blood eosinophil count (BEC) > 250 cells/μL) and n=17 eosinophil^low^ (BEC <150 cells/μL) COPD patients, in which MC involvement was highly likely(42). We subset the dataset to include the 245 protein-coding genes identified using Elastic Net and extracted normalized counts. We then calculated the average expression per each gene using the following formula, as used by *Bhakta et al.*, *Southworth et al.* and *Higham et al.*(42–44):

$$Gene count (standardized)=\frac{Gene count-Average gene expression}{Standard deviation of gene expression}$$

Standardized counts per each cytokine-activated gene composing the signatures were then averaged by subject, and unpaired t-tests, Spearman correlation and linear regression analyses were performed to compare the signatures between COPD subjects with high and low BEC using Prism 10 (Graphpad). Venn diagram comparing new and previously published IL-33-activated MC signatures(45–47), previously used in the *Higham et al.* paper, was generated using Venny v.2.1.0.(48).

- 1. ***Statistical analyses***

For surface receptor, cytokine measurement and MAT assays, repeated measures one-way ANOVA with Dunnett’s correction was used to measure statistical significance when compared to the unstimulated control. For histamine and prostaglandin D2 concentrations, Friedman test with Dunn’s correction was used. Only ANOVA/Friedman test results with p<0.05 were used for post-hoc multiple comparison analyses.

For bulk RNAseq and GSVA analyses, testing on paired samples and/or multiple comparisons were analysed using the statistical methods previously detailed, and corrected using the Benjamini-Hochberg FDR method.

For all post-hoc statistical analyses significance was set at adjusted p value <0.05.

1. **Supplemental results**
   1. ***Shared genes across cytokine stimulations include key protein kinases and early signalling transducers involved in early IgE-mediated MC activation events***

In our transcriptomics analysis, we observed 781 genes shared across all conditions (**Supplemental Table 1**). Among DEGs, 114 protein-coding genes displayed the highest fold change difference (-2<log2 fold change>2) in at least one cytokine stimulation compared to the unstimulated control (**Supplemental Figure S3A**). Enrichment analysis using Metascape(14) revealed that frequently identified shared genes are involved in a wide-range of cellular functions and signalling pathways (**Supplemental Figure S3B**). These include phosphoinositide 3-kinases (PIK3R1, PIK3R3; PI3Ks) and other protein kinases (IRAK1, PRKACB, CAM2KG, MAPK12), calmodulin (CALM2), actin/integrins involved in cell adhesion and movement (ITGB3, ACTG1, ACTB, VCL), genes involved in apoptosis (BCL2) and signal transduction (VAV3, MYD88, CSK) (**Supplemental Figure S3C**). Among significantly enriched pathways, 15 are associated with anaphylaxis, including FcεRI, MAPK, calcium signalling pathways and vesicle trafficking (**Supplemental Figure S4A**). Within FcεRI pathways, 15 gene hits were identified, including the alpha chain of the high-affinity IgE receptor (FCER1A) and genes participating in early IgE-mediated signalling events such as Linker for activation of T cells (LAT) complex formation (LAT, LCP2, GRAP2, VAV3), kinases (PIK3R1), Ras signalling (RASGRP2, RASA1), calmodulin 2 (CALM2) and nuclear factor of activated T-cells 3 (NFATC3) (**Supplemental Figure S4B-C**).

Each cytokine specifically modulated the expression of aforementioned genes. For instance, IL-4+IL-13 strongly upregulated FCER1A and NFATC3 expression, while IFNγ preferentially upregulated CALM2, and TGFβ significantly increased LAT expression. Notably, IL-33 exerted a strong inhibition of FCER1A transcription and, similarly to IL-4+IL-13, strongly upregulated phospholipid phosphatase 1 (PLPP1) expression, which is involved in sphingolipid metabolism.

We therefore identified multiple highly conserved genes involved in various signalling pathways shared across different cytokine stimulations, each uniquely modulated by the exposure to a specific cytokine. We also demonstrated that the cytokines examined play a role in the modulation of anaphylaxis, specifically early FcεRI signalling events, by influencing FcεRI receptor expression and other key intracellular signalling molecules.

**References**

1. Bahri R, Bulfone-Paus S. Mast Cell Activation Test (MAT). In: *Methods in molecular biology (Clifton, N.J.)*. 2020: 227–238.

2. Sellge G, Barkowsky M, Kramer S, Gebhardt T, Sander LE, Lorentz A et al. Interferon-γ regulates growth and controls Fcγ receptor expression and activation in human intestinal mast cells. *BMC Immunol* 2014;**15**:1–10.

3. Ra, Hano Toru C, Nonoyama S, Suzuki K, Yata J, Nakahata T, Arai K. Induction of the high-affinity IgE receptor (FcɛRI) on human mast cells by IL-4. *Int Immunol* 1996;**8**:1367–1373.

4. Rönnberg E, Ghaib A, Ceriol C, Enoksson M, Arock M, Säfholm J et al. Divergent effects of acute and prolonged interleukin 33 exposure on mast cell IgE-mediated functions. *Front Immunol* 2019;**10**:1–12.

5. Bahri R, Kiss O, Prise I, Garcia-Rodriguez KM, Atmoko H, Martínez-Gómez JM et al. Human Melanoma-Associated Mast Cells Display a Distinct Transcriptional Signature Characterized by an Upregulation of the Complement Component 3 That Correlates With Poor Prognosis. *Front Immunol* 2022;**13**. doi:10.3389/fimmu.2022.861545

6. Wingett SW, Andrews S. FastQ Screen: A tool for multi-genome mapping and quality control. *F1000Research 2018 71338* 2018;**7**:1338.

7. Bushnell B, Rood J, Singer E. BBMerge – Accurate paired shotgun read merging via overlap. *PLoS One* 2017;**12**:e0185056.

8. Frankish A, Diekhans M, Ferreira A-M, Johnson R, Jungreis I, Loveland J et al. GENCODE reference annotation for the human and mouse genomes. *Nucleic Acids Res* 2019;**47**:D766–D773.

9. Dobin A, Davis CA, Schlesinger F, Drenkow J, Zaleski C, Jha S et al. STAR: ultrafast universal RNA-seq aligner. *Bioinformatics* 2013;**29**:15–21.

10. Love MI, Huber W, Anders S. Moderated estimation of fold change and dispersion for RNA-seq data with DESeq2. *Genome Biol 2014 1512* 2014;**15**:1–21.

11. Wickham H. *ggplot2*. Cham: Springer International Publishing 2016 doi:10.1007/978-3-319-24277-4

12. Kolde R. CRAN - Package pheatmap. https://cran.r-project.org/web/packages/pheatmap/. 2019.https://cran.r-project.org/web/packages/pheatmap/ (accessed 24 Jul2021).

13. Yan L. Draw Venn Diagram by ‘ggplot2’ [R package ggvenn version 0.1.9]. Published Online First: 29 June 2021.https://cran.r-project.org/package=ggvenn (accessed 24 Jul2021).

14. Zhou Y, Zhou B, Pache L, Chang M, Khodabakhshi AH, Tanaseichuk O et al. Metascape provides a biologist-oriented resource for the analysis of systems-level datasets. *Nat Commun 2019 101* 2019;**10**:1–10.

15. Kanehisa M, Furumichi M, Tanabe M, Sato Y, Morishima K. KEGG: new perspectives on genomes, pathways, diseases and drugs. *Nucleic Acids Res* 2017;**45**:D353–D361.

16. The Gene Ontology Consortium. The Gene Ontology Resource: 20 years and still GOing strong. *Nucleic Acids Res* 2019;**47**:D330–D338.

17. Fabregat A, Sidiropoulos K, Viteri G, Forner O, Marin-Garcia P, Arnau V et al. Reactome pathway analysis: a high-performance in-memory approach. *BMC Bioinforma 2017 181* 2017;**18**:1–9.

18. Slenter DN, Kutmon M, Hanspers K, Riutta A, Windsor J, Nunes N et al. WikiPathways: a multifaceted pathway database bridging metabolomics to other omics research. *Nucleic Acids Res* 2018;**46**:D661–D667.

19. Han H, Cho J-W, Lee S, Yun A, Kim H, Bae D et al. TRRUST v2: an expanded reference database of human and mouse transcriptional regulatory interactions. *Nucleic Acids Res* 2018;**46**:D380–D386.

20. Szklarczyk D, Gable AL, Lyon D, Junge A, Wyder S, Huerta-Cepas J et al. STRING v11: protein–protein association networks with increased coverage, supporting functional discovery in genome-wide experimental datasets. *Nucleic Acids Res* 2019;**47**:D607–D613.

21. Bader GD, Hogue CW. An automated method for finding molecular complexes in large protein interaction networks. *BMC Bioinforma 2003 41* 2003;**4**:1–27.

22. Shannon P, Markiel A, Ozier O, Baliga NS, Wang JT, Ramage D et al. Cytoscape: A Software Environment for Integrated Models of Biomolecular Interaction Networks. *Genome Res* 2003;**13**:2498.

23. Tay JK, Narasimhan B, Hastie T. Elastic Net Regularization Paths for All Generalized Linear Models. *J Stat Softw* 2023;**106**. doi:10.18637/JSS.V106.I01

24. Kuhn M. Building Predictive Models in R Using the caret Package. *J Stat Softw* 2008;**28**:1–26.

25. CRAN: Package neuralnet. https://cran.r-project.org/web/packages/neuralnet/index.html (accessed 22 Oct2024).

26. Robin X, Turck N, Hainard A, Tiberti N, Lisacek F, Sanchez JC et al. pROC: An open-source package for R and S+ to analyze and compare ROC curves. *BMC Bioinformatics* 2011;**12**:1–8.

27. Rochman M, Wen T, Kotliar M, Dexheimer PJ, Ben-Baruch Morgenstern N, Caldwell JM et al. Single-cell RNA-Seq of human esophageal epithelium in homeostasis and allergic inflammation. *JCI Insight* 2022;**7**. doi:10.1172/jci.insight.159093

28. Ben-Baruch Morgenstern N, Ballaban AY, Wen T, Shoda T, Caldwell JM, Kliewer K et al. Single-cell RNA sequencing of mast cells in eosinophilic esophagitis reveals heterogeneity, local proliferation, and activation that persists in remission. *J Allergy Clin Immunol* 2022;**149**:2062–2077.

29. Zheng M, Hu Z, Mei X, Ouyang L, Song Y, Zhou W et al. Single-cell sequencing shows cellular heterogeneity of cutaneous lesions in lupus erythematosus. *Nat Commun 2022 131* 2022;**13**:1–17.

30. Le Floc’h A, Allinne J, Nagashima K, Scott G, Birchard D, Asrat S et al. Dual blockade of IL‐4 and IL‐13 with dupilumab, an IL‐4Rα antibody, is required to broadly inhibit type 2 inflammation. *Allergy* 2020;**75**:1188–1204.

31. Sikkema L, Ramírez-Suástegui C, Strobl DC, Gillett TE, Zappia L, Madissoon E et al. An integrated cell atlas of the lung in health and disease. *Nat Med* 2023;**29**:1563–1577.

32. Liu Y, Wang H, Taylor M, Cook C, Martínez-Berdeja A, North JP et al. Classification of human chronic inflammatory skin disease based on single-cell immune profiling. *Sci Immunol* 2022;**7**. doi:10.1126/sciimmunol.abl9165

33. West PW, Tontini C, Atmoko H, Kiss O, Garner T, Bahri R et al. Human Mast Cells Upregulate Cathepsin B, a Novel Marker of Itch in Psoriasis. *Cells* 2023;**12**. doi:10.3390/CELLS12172177

34. Hao Y, Stuart T, Kowalski MH, Choudhary S, Hoffman P, Hartman A et al. Dictionary learning for integrative, multimodal and scalable single-cell analysis. *Nat Biotechnol* 2024;**42**:293–304.

35. Street K, Risso D, Fletcher RB, Das D, Ngai J, Yosef N et al. Slingshot: cell lineage and pseudotime inference for single-cell transcriptomics. *BMC Genomics* 2018;**19**:477.

36. Brunson J. ggalluvial: Layered Grammar for Alluvial Plots. *J Open Source Softw* 2020;**5**:2017.

37. Gu Z. Complex heatmap visualization. *iMeta* 2022;**1**. doi:10.1002/imt2.43

38. Hänzelmann S, Castelo R, Guinney J. GSVA: Gene set variation analysis for microarray and RNA-Seq data. *BMC Bioinformatics* 2013;**14**:1–15.

39. McCarthy DJ, Campbell KR, Lun ATL, Wills QF. Scater: pre-processing, quality control, normalization and visualization of single-cell RNA-seq data in R. *Bioinformatics* 2017;**33**:1179–1186.

40. Robinson MD, McCarthy DJ, Smyth GK. edgeR: a Bioconductor package for differential expression analysis of digital gene expression data. *Bioinformatics* 2010;**26**:139–140.

41. Ritchie ME, Phipson B, Wu D, Hu Y, Law CW, Shi W et al. limma powers differential expression analyses for RNA-sequencing and microarray studies. *Nucleic Acids Res* 2015;**43**:e47–e47.

42. Higham A, Dungwa J, Pham T, McCrae C, Singh D. Increased mast cell activation in eosinophilic chronic obstructive pulmonary disease. *Clin Transl Immunol* 2022;**11**. doi:10.1002/cti2.1417

43. Bhakta NR, Solberg OD, Nguyen CP, Nguyen CN, Arron JR, Fahy J V. et al. A qPCR-based metric of Th2 airway inflammation in asthma. *Clin Transl Allergy* 2013;**3**:24.

44. Southworth T, Van Geest M, Singh D. Type‐2 airway inflammation in mild asthma patients with high blood eosinophils and high fractional exhaled nitric oxide. *Clin Transl Sci* 2021;**14**:1259–1264.

45. Badi YE, Salcman B, Taylor A, Rana B, Kermani NZ, Riley JH et al. IL1RAP expression and the enrichment of IL-33 activation signatures in severe neutrophilic asthma. *Allergy* 2023;**78**:156–167.

46. Tiotiu A, Badi Y, Kermani NZ, Sanak M, Kolmert J, Wheelock CE et al. Association of Differential Mast Cell Activation with Granulocytic Inflammation in Severe Asthma. *Am J Respir Crit Care Med* 2022;**205**:397–411.

47. Nagarkar DR, Ramirez-Carrozzi V, Choy DF, Lee K, Soriano R, Jia G et al. IL-13 mediates IL-33-dependent mast cell and type 2 innate lymphoid cell effects on bronchial epithelial cells. *J Allergy Clin Immunol* 2015;**136**:202–205.

48. Oliveros JC. Venny. An interactive tool for comparing lists with Venn’s diagrams. https://bioinfogp.cnb.csic.es/tools/venny/index.html (accessed 29 Oct2024).

1. **Supplemental Tables and Figures**

**Supplemental Table 1. Shared and unique differentially expressed genes per cytokine stimulation measured using DESeq2.** Shared differentially expressed genes across cytokines (n=781) and highly up-/down-regulated genes per cytokine stimulation (log2FC >5 or <-2) are given as log2 fold change compared to unstimulated and significance adjusted by the false discovery rate method.

(see **Supporting Information Data S1**)

**Supplemental Table 2. Frequency of marker-positive mast cells following cytokine stimulation measured in flow cytometry.**

| **Surface receptor** | **n.** | **Unstimulated** | | **IFNγ** | | **IL-33** | | **IL-4+IL-13** | | **TGFβ** | |
| --- | --- | --- | --- | --- | --- | --- | --- | --- | --- | --- | --- |
|  |  | mean ± SD | Adj. p value | mean ± SD | Adj. p value | mean ± SD | Adj. p value | mean ± SD | Adj. p value | mean ± SD | Adj. p value |
| CD117 (c-KIT) | 6 | 44.9 ± 41.4 | - | 46.0 ± 42.0 | ns | 52.6 ± 34.3 | ns | **42.1 ± 40.3** | **0.008** | 36.3 ± 35.1 | ns |
| FcεRIα | 3 | 50.3 ± 12.9 | - | 43.8 ± 14.9 | ns | 20.7 ± 2.9 | ns | **70.0 ± 15.5** | **0.048** | **29.6 ± 10.5** | **0.028** |
| MRGPRX2 | 6 | 23.5 ± 17.0 | - | 22.9 ± 20.8 | ns | 15.3 ± 10.8 | ns | 23.6 ± 17.1 | ns | 29.8 ± 16.7 | ns |
| FcγRII/CD32 | 6 | 15.0 ± 11.5 | - | 14.0 ± 10.6 | ns | **59.1 ± 27.9** | **0.013** | 16.7 ± 11.9 | ns | 15.1 ± 11.7 | ns |
| HLA-DR | 4 | 1.9 ± 0.7 | - | 40.8 ± 32.9 | ns | 1.5 ± 0.6 | ns | 1.9 ± 0.7 | ns | 1.9 ± 0.8 | ns |
| Integrin αvβ3 (CD51-CD61) | 3 | 52.9 ± 15.8 | - | 45.6 ± 20.5 | ns | 57.8 ± 19.3 | ns | 46.0 ± 22.3 | ns | 69.8 ± 11.35 | ns |

One-way ANOVA with Dunnett’s multiple comparisons adjustment.

Abbreviations: FcεRI: High affinity Immunoglobulin E receptor, FcγRII: Low affinity Immunoglobulin G receptor II, HLA-DR: Major histocompatibility complex class II DR alpha, MRGPRX2: Mas-Related G Protein-Coupled Receptor-X2.

**Supplemental Table 3. Minimal cytokine-activated signatures identified using Elastic Net.**

| **IFNγ**  **(n=69)** | **IL-33**  **(n=78)** | **IL-4+IL-13**  **(n=26)** | **TGFβ**  **(n=72)** |
| --- | --- | --- | --- |
| AC040162.1 | ADAP1 | ABCB4 | AK1 |
| AMER1 | ALG14 | ABI2 | ANKMY1 |
| ANKIB1 | AMMECR1 | ACAP1 | ANXA1 |
| APOBEC3F | ANAPC5 | C12orf29 | APC |
| APOL3 | ANXA7 | CERS5 | APEX2 |
| APOL4 | APEX1 | CNKSR2 | APOC1 |
| ATF3 | BCAR3 | CNR2 | ATP1A1 |
| ATP6V1B2 | BEX4 | CSPG5 | BCL9L |
| BAK1 | C16orf87 | DHRS11 | BEND3 |
| BAZ2A | CACNA1B | DNAH2 | BUB3 |
| BBX | CCDC124 | GNA11 | C11orf1 |
| BTN3A3 | CCR7 | GOLGB1 | C1QTNF6 |
| C4A | CD37 | NXPH3 | CADM4 |
| C4B | CD70 | ODF2 | CALM3 |
| CASP8 | CDH13 | PKIG | CAPN10 |
| CBR1 | CDK15 | RNF182 | CCPG1 |
| CDKL1 | CEP135 | SDF2 | CENPO |
| CR1L | CEPT1 | SLC25A6 | CKAP5 |
| CTSO | CREB3 | SOAT2 | CMTM1 |
| CXorf38 | CRTAC1 | SPINT2 | COMP |
| CYREN | CSTF2 | SPOP | CRTC3 |
| DOCK9 | CTPS1 | TBC1D13 | DHRS4 |
| EFR3A | DAG1 | TMEM14A | EMILIN2 |
| FBXO7 | DHRS1 | TMEM50A | FCAMR |
| FRMD3 | DNAJB9 | WNT5B | FHL3 |
| GTPBP1 | EEF2KMT | ZBED5 | FSTL3 |
| GUK1 | FEZ1 |  | GATAD2B |
| HLA-DOB | GATA2 |  | GINS3 |
| IFI27 | GCGR |  | GNB5 |
| IFI44L | GNPTAB |  | GPANK1 |
| IFI6 | GPR50 |  | GPR35 |
| IL15 | HDLBP |  | HOXB6 |
| IRF7 | HSPA6 |  | ILKAP |
| ISG15 | IER5 |  | LASP1 |
| ISG20 | IL13 |  | LBX2 |
| LARP1 | IL7R |  | MALT1 |
| LGALS3BP | ILF2 |  | MAP3K2 |
| MICB | ITGAX |  | MBD4 |
| MMAA | KLF5 |  | NR2C2 |
| MX2 | LRMDA |  | NSMCE2 |
| NEXN | MAMLD1 |  | NSUN5 |
| NMI | MRPS33 |  | OR1N1 |
| NMT1 | MTFP1 |  | PAIP2B |
| OASL | NABP2 |  | PFKFB4 |
| ODF3B | NIPBL |  | PNMA8C |
| OGFR | NLE1 |  | POLD4 |
| PARP10 | NLGN3 |  | PROC |
| PEX12 | NMNAT3 |  | RASGEF1C |
| PSMB10 | NPAT |  | RD3 |
| RBBP6 | P2RX5 |  | RFWD3 |
| RBCK1 | PDCD11 |  | SAMD11 |
| RMDN3 | PFAS |  | SCG5 |
| SCO2 | PIGV |  | SDCCAG8 |
| SECTM1 | POLDIP2 |  | SEPTIN11 |
| SERPING1 | POMT2 |  | SH3BP1 |
| SHFL | POU2F2 |  | SLC10A5 |
| SLC25A28 | PPARD |  | SLC20A2 |
| STAT2 | PRKRA |  | SMIM24 |
| STX4 | PSMD4 |  | SSH1 |
| SUMO2 | PVR |  | STING1 |
| TBX21 | RAB4A |  | TAF12 |
| TMEM140 | RAPH1 |  | TAF1D |
| TNFSF13B | REL |  | TCEAL9 |
| TTLL6 | SAMD15 |  | TCEANC2 |
| UBR2 | SEC14L2 |  | TCFL5 |
| USF1 | SNUPN |  | TMEM130 |
| XRN1 | SRA1 |  | TMEM248 |
| ZKSCAN5 | SRPRB |  | UQCRFS1 |
| ZNF267 | TNIP1 |  | WBP1L |
|  | TP53 |  | ZCCHC7 |
|  | TP53RK |  | ZNF34 |
|  | TRAF1 |  | ZNF41 |
|  | TRPM6 |  |  |
|  | TSPAN18 |  |  |
|  | TTC39A |  |  |
|  | UNC119 |  |  |
|  | WDR12 |  |  |
|  | ZFHX2 |  |  |

**Supplemental Table 4. Full list of gene overlaps between currently available human IL-33-primed MC signatures.**

| **Study names** | **Overlaps** | **Genes** |
| --- | --- | --- |
| *Badi et al.* (upregulated) ∪  *Tiotiu et al.*/*Nagarkar et al.* ∪  *Tontini et al.* | 4 | CD70 IL13 KLF5 TNIP1 |
| *Badi et al.* (upregulated) ∪  *Tontini et al.* | 18 | BCAR3 CACNA1B CCR7 CDH13 CRTAC1 FEZ1 GCGR GPR50 IL7R MAMLD1 NMNAT3 POU2F2 SAMD15 TP53RK TRAF1 TRPM6 TSPAN18 TTC39A |
| *Badi et al.* (upregulated) ∪  *Tiotiu et al.*/*Nagarkar et al.* | 36 | ABTB2 ADAM8 C15orf48 CCL3 CXCL8 EBI3 GDF11 HAPLN3 HIVEP2 IL1B IL2RG IL5 ITGA1 KCNH2 KISS1R LIMS2 MMP9 MT2A NFKBIA NFKBIZ PEAK1 PIM2 RGS9 RIPOR3 SSTR2 TLR2 TMEM64 TNF TNFRSF18 TNFRSF1B TNFRSF8 TNFSF14 TNFSF4 TTC39C VWA8 ZC3H12A |
| *Tiotiu et al.*/ *Nagarkar et al.* ∪  *Tontini et al.* | 3 | AMMECR1 CEP135 P2RX5 |
| *Badi et al.* (downregulated) ∪ *Tontini et al.* | 1 | CDK15 |
| *Badi et al.* (upregulated) | 360 | AC007278.1 AC007278.2 AC007384.1 AC010247.1 AC011611.3 AC026310.1 AC034102.1 AC034199.1 AC078785.1 AC083837.1 AC093503.2 AC099552.1 AC107959.3 AC110611.2 AC114977.1 AC120498.6 AC124798.1 AC245100.7 ACE ACHE ADGRA3 AGMAT AGRN AHRR AKR1C3 AL031283.1 AL133520.1 AL137060.3 AL160408.3 AL445524.1 AL645608.8 ALDH8A1 ALOX15 ANK2 APOC1 AQP3 ARHGAP23 ARNT2 ASB2 ASIC1 ATOX1 B4GALNT1 BAALC BAMBI BATF BCL9 BIRC3 BX255923.2 C1orf53 C1QTNF1 C3 CA12 CACNA1H CARD11 CBS CCDC102A CCDC6 CCL1 CCL24 CCL4 CCL5 CD200 CD36 CD40 CD74 CD81 CDHR1 CFAP46 CGN CHGA CHPF CHST1 CHST7 CLCN4 CNR2 CNTNAP1 COL13A1 COL15A1 COL16A1 COL5A3 COL6A5 COL7A1 COL9A2 COLGALT2 CPNE3 CSF2 CTSH CTTN CTXN1 CXCR5 CYP1A1 CYP27B1 CYP4F22 CYP7B1 CYTIP DAB2IP DIXDC1 DLEU1 DLEU2 DNPH1 DPP4 DSE DYRK3 EDARADD EDIL3 EFCAB12 EGR2 ENPP1 EPOP FAM186B FBXL19-AS1 FCER2 FCRLA FERMT1 FHL1 FIRRE FKBP5 FLVCR2 FN1 FRMD4A GADL1 GAS6 GBP4 GCH1 GCKR GJA4 GLIS2 GOLGA2P10 GP1BA GPC6 GPR68 GPRC5C GSTP1 H1-2 H2AC6 H2BC11 H2BC12 H2BC4 H2BC5 HLA-B HLA-DPB1 HLA-DRA HLF IGFBP4 IGFL2 IGLV2-18 IGLV3-17 IGLV3-19 IGLV3-21 IL10 IL15RA IL17RB IL18RAP IL19 IL1A IL27RA IL2RA IL2RB IL32 IL34 IL4I1 INA INHBA IRAK2 IRF4 ITGB4 KCNK13 KCNK5 KIAA1958 KIF26B KIRREL3 LAMP3 LHFPL6 LINC00475 LINC00892 LINC01134 LINC01215 LINC01285 LINC02068 LINC02605 LINC02690 LINC02694 LOXL4 LRFN5 LRG1 LRIG1 LST1 LTA LTB MAOA MAP3K8 MAP7D2 MARCKS MARCKSL1 MEX3A MFSD12 MFSD2A MGAT5B MGP MIAT MIR155HG MIR3142HG MMP11 MMP19 MMP2 MMP2-AS1 MMP7 MRAP2 MRC1 MREG MT1DP MT1E MT1G MT1L MT2P1 MTHFD1L MUC20 MUC4 MYDGF MYL9 MYO1E N4BP3 NACAD NAMPTP1 NAV3 NEDD4L NEK10 NETO2 NFKB2 NMB NME1 NOS3 NR4A3 NT5DC2 NTN1 NUAK1 OAS3 OCIAD2 OLFM2 OLR1 P2RX5-TAX1BP3 P4HA2 P4HB PCSK5 PCSK9 PDE4C PDIA5 PDLIM4 PGAP4 PHGDH PIEZO2 PIFO PIGR PKM PKMP1 PLA2G4A PLA2G4C PPP1R3C PRCP PRG2 PRSS23 PSAT1 PSD PTGES PTGES3L PTGIR PTGS2 PTPN13 PXYLP1 PYCR1 QPCT RAI2 RASGRP1 RASGRP3 RASL11A RCN3 RDH10 REEP2 RELB RGL1 RGS3 ROBO1 ROR1 ROR1-AS1 RRAD RYR1 S100A9 SBK1 SCG2 SCN9A SDF2L1 SEC11C SEMA4B SEPTIN4 SERPIND1 SERPINE1 SERPINF1 SFRP2 SGPP2 SHC4 SHISA2 SLAMF7 SLC16A1 SLC16A9 SLC22A31 SLC27A2 SLC7A11 SLCO4A1 SLCO5A1 SMAD3 SORCS2 SORD2P SPAG1 SPARC SPATC1 SPTA1 ST6GAL1 STARD10 STAT4 SYNGR3 SYPL2 SYT12 TCERG1L TENT5A TGFBR3 TGM2 THBS1 TIGIT TINCR TJP1 TLL2 TLR7 TMEM120A TMEM176A TMEM176B TMEM198 TMEM217 TMEM25 TNFAIP2 TNFAIP3 TNFRSF9 TNFSF9 TNS3 TPSG1 TRIM9 TYMSOS UBE2E2 VEGFC VTN VWCE WFS1 WLS WNT9A WT1 XIRP1 ZBTB7C ZCCHC12 ZFPM1 ZFPM2 ZNF804A |
| *Badi et al.* (downregulated) | 352 | ABHD4 ABLIM3 AC004233.2 AC006230.1 AC009145.1 AC009652.1 AC010175.1 AC010210.1 AC011899.2 AC018450.1 AC018865.2 AC019197.1 AC020907.5 AC020914.3 AC060766.6 AC073869.3 AC080013.3 AC087369.2 AC091563.1 AC092650.1 AC104248.1 AC104964.2 AC108053.1 AC112178.1 AC113414.1 AC116424.1 ADAM11 ADAMTSL3 ADGRE4P ADGRG4 ADGRG7 ADRA1D ADRA2A AIF1 AK5 AL021392.1 AL021917.1 AL049552.2 AL078590.2 AL133313.1 AL136018.1 AL136456.1 AL139142.1 AL355365.1 AL356489.1 AL356489.2 AL356489.3 AL356804.1 AL590617.2 AL670729.3 ANK3 ANLN ANXA8 ANXA8L1 AP001122.1 AP003119.1 APBB1IP ARAP3 ARHGEF37 ARHGEF40 ARL15 ARRDC4 ASPM ATP1B1 AURKB B3GNT5 BIRC7 BTBD19 BTG1P1 BX571818.1 C11orf45 C12orf42 C1orf127 C3AR1 CA8 CACNG8 CALB1 CALCRL CCDC188 CCDC30 CCNA1 CD2 CD226 CDH23 CDK1 CDSN CFAP58-DT CHST15 CHST8 CIDEB CLDN9 CNIH3 COBLL1 COL25A1 CPED1 CPNE5 CR1 CREB3L1 CREG2 CRYBB1 CSF2RA CSF3R CTSG CXCL16 CYP4F30P CYRIA DEPTOR DHRS9 DLGAP5 DNAH8 DNM1P35 DPY19L2P1 E2F8 EEF1A1P14 EFHC2 EGFEM1P EGR3 ELFN1 EMP1 ERVFRD-1 ESR2 EVPL F2R F8 FAM111B FAM20A FAM216B FAR2P1 FAR2P4 FBP1 FCER1A FMN1 FOXF1 FOXN2 FREM1 GATA3 GCSAM GFOD1 GJA3 GPR162 GPR34 HACD4 HBD HGD HK3 HNF4A-AS1 HPN HS3ST1 ICAM4 ICAM5 IFI30 IL12A-AS1 IL16 IL18BP INKA1 IQGAP3 IRAG1 ITGA3 ITPR3 JPH4 KCNJ5 KCNK3 KIAA0040 KIAA1614 KIF18B KLF2P4 KREMEN1 KRT17P8 KRT72 KRT73 KY LBH LILRB2 LILRB3 LINC00323 LINC00607 LINC00987 LINC01366 LINC01529 LINC01583 LINC01918 LINC02008 LINC02082 LINC02115 LINC02147 LINC02395 LINC02656 LINC02773 LINC02829 LINCR-0001 LIPN LOXL3 LRRC7 LTB4R LTB4R2 LURAP1L M1AP MAF MAGEB2 MAS1L MAS1LP1 MATK MIR223HG MIR3681HG MKI67 MLPH MOB3B MPIG6B MRGPRX2 MRGPRX6P MRGPRX7P MROH7 MS4A3 MS4A6A MYLK MYO16 NAALADL2 NABP1 NDRG2 NEURL1B NFE2 NHSL1 NLRC4 NPTX1 NRGN NTM OR10V3P OR10Y1P OR8A1 OR8G1 OR8G3P OR8G5 OTOA P2RY10 PADI2 PANX3 PDE2A PDE7B PDK4 PDZPH1P PEPD PICART1 PIK3IP1 PITPNM3 PLEKHA5 PLXDC1 PLXNA2 PLXNA4 PM20D1 PPIAP41 PPP2R2B PRKG2 PRR5L PTCH2 PTENP1-AS PTPRN2-AS1 PVALB RAB3B RALGPS2 RASGEF1B RASGEF1C RASL10A RBM20 REM1 REXO5 RGS13 RGS4 RGS5 RHBDF1 RHOQP3 RNASE6 RPS17P1 RTN4R RYR3 S100Z S1PR1 SCIN SCN1B SCN2A SCN7A SCPEP1 SDK2 SEMA3D SEMA6B SEPTIN7P9 SERPINB1 SERPINB9P1 SESN3 SFTPD SGIP1 SGK1 SGSM1 SHANK1 SHC3 SIGLEC12 SIGLEC15 SIPA1L2 SLA SLC15A2 SLC22A10 SLC22A18 SLC22A18AS SLC27A3 SLC37A2 SLC38A11 SLC38A6 SLFN14 SMAD6 SNCG SNX21 SNX29 SORBS3 SPINK4 SSPOP ST14 STAB1 SVOPL SYN3 SYNE2 TBC1D2 TCTEX1D1 TGM5 TIMP2 TMCC3 TNFRSF14-AS1 TNNI2 TNS1 TNXB TOX2 TP53I11 TRBV26OR9-2 TREML2 TSPAN11 TSPYL2 TTN TXLNB TXNIP UBE2C UBE2D3-AS1 UNC79 UNC80 UTS2 VWA5B2 VWC2 Z85996.3 ZBTB16 ZBTB20 ZMAT4 ZMYND15 ZNF367 ZNF467 ZNF483 |
| *Tontini et al.* | 53 | ADAP1 ALG14 ANAPC5 ANXA7 APEX1 BEX4 C16orf87 CCDC124 CD37 CEPT1 CREB3 CSTF2 CTPS1 DAG1 DHRS1 DNAJB9 EEF2KMT GATA2 GNPTAB HDLBP HSPA6 IER5 ILF2 ITGAX LRMDA MRPS33 MTFP1 NABP2 NIPBL NLE1 NLGN3 NPAT PDCD11 PFAS PIGV POLDIP2 POMT2 PPARD PRKRA PSMD4 PVR RAB4A RAPH1 REL SEC14L2 SNUPN SRA1 SRPRB TP53 UNC119 WDR12 ZFHX2 |
| *Tiotiu et al.*/*Nagarkar et al.* | 43 | ADIPOR2 AREG ASNS CCL3L3 CDC42EP2 CFLAR CXCL2 CXCL6 DES EGF EGLN1 EREG FGD6 FLVCR1 GADD45B GDF3 IER3 IL17D IL3 IL36G IL6 KIAA1324 KITLG LARGE2 METAP2 MIIP MPZL1 NAPB NDP NFKB1 PMAIP1 PTX3 RANBP9 RMI1 SLC25A45 SLC4A7 TAP2 TEX30 TNFRSF17 TNFSF11 UBD WNT11 WNT2B |

**Supplemental Figure captions**

**Supplemental Figure S1. Schematic experimental workflow.** Primary human mast cells (MCs) were exposed to different cytokine concentrations for 24 hours and used for transcriptomics, surface expression and functional analyses. Publicly available bulk and single-cell transcriptomics datasets were downloaded from Gene Expression Omnibus, CellXGene and Zenodo and used for gene set variation and single-cell pseudotime analyses. Icon created using Biorender.com.

Abbreviations: CD32/CD32b-c: Low affinity Immunoglobulin G receptor II/b-c, CD51-CD61: Integrin αvβ3, CD117: Stem cell factor receptor c-KIT, COPD: Chronic obstructive pulmonary disease, FcεRI: High affinity Immunoglobulin E receptor, GM-CSF: Granulocyte-Monocyte Colony Stimulating Factor, HLA-DR: Major histocompatibility complex class II DR, IgE: Immunoglobulin E, IFNγ: Interferon gamma, IgG: Immunoglobulin G, IL-4: Interleukin 4, IL-5: Interleukin 5, IL-8/CXCL8: Interleukin 8, IL-10: Interleukin 10, IL-13: Interleukin 13, MCP-1: Monocyte Chemoattractant Protein-1 (CCL2), MIP-1α: Macrophage Inflammatory Protein-1 Alpha (CCL3), MRGPRX2: Mas-Related G Protein-Coupled Receptor-X2, scRNAseq: single-cell RNA sequencing, TGFβ: Transforming growth factor beta.

**Supplemental Figure S2. Primary human mast cell maturation.** Representative gating strategy (n=1) for mature primary human mast cells definition following differentiation from peripheral blood progenitors *in vitro*. Unstained (blue) and stained samples (red) were overlaid in FlowJo.

**Supplemental Figure S3. Differentially expressed genes among cytokine stimulations and gene set enrichment of shared signatures.**

Primary human MCs (n=4) were incubated for 24 hours with IFNγ 50 ng/ml, IL-33 50 ng/ml, IL-4+IL-13 10 ng/ml and TGFβ 5 ng/ml. Total mRNA was extracted for bulk sequencing and analysed using DESeq2. A) Heatmap showing highly up- and down-regulated protein-coding genes among all DEGs (-2< log2 fold change >2) was created using pheatmap(12); B) Gene enrichment analysis of shared genes across stimulations (n=781) was performed using Metascape(14) and plotted using the ggplot2 package; C) Top gene hits represented in 20 or more pathways identified using Metascape enrichment was plotted using WordClouds.co.uk.

**Supplemental Figure S4. Fifteen FcεRI signalling pathway-associated genes are shared across different cytokine stimulations.** Primary human MCs (n=4) were incubated for 24 hours with IFNγ 50 ng/ml, IL-33 50 ng/ml, IL-4+IL-13 10 ng/ml and TGFβ 5 ng/ml. Total mRNA was extracted for bulk sequencing and analysed using DESeq2. A) Pathways associated with MC degranulation and/or anaphylaxis showing positive enrichment in Metascape enrichment analysis of shared genes (n=781). Dot plot of adjusted p values was created using ggplot2; B) Heatmap of the 15 gene hits of the enriched FcεRI pathways was created using the R package pheatmap; C) Schematic representation of the FcεRI signalling pathway was created using Biorender.com. Gene hits are highlighted in purple.

**Supplemental Figure S5. Enrichment analysis of unique upregulated cytokine-activated gene signatures.** Primary human MCs (n=4) were incubated for 24 hours with IFNγ 50 ng/ml, IL-33 50 ng/ml, IL-4+IL-13 10 ng/ml and TGFβ 5 ng/ml. Total mRNA was extracted for bulk sequencing and analysed using DESeq2. Unique upregulated (log2 fold change >1) differentially expressed genes were analysed using Metascape. A) Venn diagram of shared upregulated protein-coding genes was created using ggvenn. B) Circos plot of significantly shared enriched pathways across stimulations was generated using Metascape. C) Heatmaps of significantly enriched pathways and ontology databases and D) Transcriptional Regulatory Relationships Unraveled by Sentence-based Text mining (TRRUST) analysis identified across different stimulations were generated using the R package pheatmap. Displayed log 10 p adjusted values measured in Metascape.

Abbreviations: KEGG: Kyoto Encyclopedia of Genes and Genomes.

**Supplemental Figure S6. Flow cytometry gating strategy for surface receptor expression measurement.** Primary human MCs were incubated with different cytokines for 24 hours. Surface receptors of interest were stained using fluorochrome-conjugated antibodies and samples, alongside unstained and fluorescence minus one (FMO) controls, were acquired in flow cytometry. Representative plots combining n=3 experiments were generated using FlowJo.

**Supplemental Figure S7. IL-33 primes MCs to mixed IgE-IgG responses.**

(A) Primary human MCs (n=4-5) were incubated for 24 hours with IFNγ 50 ng/ml, IL-33 50 ng/ml, IL-4+IL-13 10 ng/ml and TGFβ 5 ng/ml, while sensitized overnight with human myeloma IgE 100 ng/ml. Cells were washed and stimulated with anti-IgE (αIgE) 50 ng/ml or media for 1 hour. Representative plots from n=1 IgE/anti-IgE experiment were generated using FlowJo. MCs were stained with fluorochrome-conjugated antibodies against CD63 and/or CD107a. (B) Upregulation of activation markers CD63 and CD107a was measured in flow cytometry. Repeated measures two-way ANOVA with Dunnett’s correction was used for the statistical comparisons between different cytokines and the unstimulated control, media and αIgE stimulation. For ANOVA, significance was set at p<0.05, while for multiple comparisons adjusted p values <0.05 were considered significant. (C) Primary human MCs (n=4) were stimulated for 24 hours with IL-33 or left unstimulated. Cells were then washed and incubated with anti-human Fcγ receptor IgG solution for 10 minutes and then stimulated with mouse anti-human FcεRI, with or without mouse IgG2a, and cross-linked with anti-mouse IgG lambda chain to induce immunocomplexes. Activation was then measured via CD63 upregulation in flow cytometry. Repeated measures one-way ANOVA with Dunnett’s correction was used for the statistical comparisons between IL-33 and unstimulated control. Data presented as mean ± standard deviation. *Adjusted p<0.05, ** p<0.01 *** p<0.001.

**Supplemental Figure S8. Feature selection of different cytokine-activated signatures using machine learning approaches.** Signatures extracted from unique differentially expressed genes upon cytokine stimulation of primary human MCs were reduced using the Elastic Net algorithm. (A-E) Feature importance coefficients of selected genes were computed using the R glmnet function and plotted per each cytokine class. (F) One-versus-all receiver operator curves of a second validation model computed using the package neuralnet per each cytokine class and plotted using the package pROC.

**Supplemental Figure S9. Validation of cytokine-activated MC signatures using publicly available transcriptomics MC datasets.** Minimal cytokine-activated signatures from uniquely expressed genes by single stimulations were selected using Elastic Net. Gene set variation analysis was calculated per each cytokine signature and validated using tissue MCs bulk/pseudobulk counts obtained from the reanalysis of publicly available datasets (A-C) informed by the results of the original authors and plotted using ggplot2. D) Venn diagram of differentially expressed genes across datasets compared to the minimal cytokine-activated signatures was created using Venny. E) List of differentially expressed genes represented in the validation datasets per cytokine signature.

Data presented as mean ± standard deviation. *False discovery rate adjusted p<0.05, *** p<0.001, **** p<0.0001

Abbreviations: DLE: discoid lupus erythematosus, Fel d 1: major cat allergen 1, EoE: eosinophilic esophagitis, SLE: systemic lupus erythematosus.

**Supplemental Figure S10. IFNγ-primed MC signatures are represented in most psoriasis skin lesions.** Minimal cytokine-activated signatures from uniquely expressed genes by single stimulations were selected using Elastic Net. Gene set variation analysis was calculated for each cytokine signature on bulk transcriptomics dataset of healthy (n=7) and psoriasis patients (n=6, 2 pooled) and plotted using ggplot2.

**Supplemental Figure S11. Single-cell and pseudotime trajectory analyses of skin MCs from healthy, atopic dermatitis and psoriasis subjects.**

MC clusters from the *Liu et al.* single-cell dataset were extracted and re-analysed using the R package Seurat. A) UMAP of the found skin MC clusters, B) relative z-score expression of the minimal cytokine-activated signatures, C) cluster trajectory network analysis of MC clusters and D) progression of the individual cytokine-activated signatures along Trajectory 1 pseudotime were plotted using ggplot2. Vertical grey dashed lines represent tertile-defined phase thresholds (Early-Mid-Late).

**Supplemental Figure S12. Single-cell and pseudotime cluster trajectory network of nasal/lung MCs in health and disease.**

MC clusters from the *Sikkema et al.* single-cell dataset were extracted and re-analysed using the R package Seurat. A) UMAP of the found lung/nasal MC clusters, B) z-score expression of minimal cytokine-activated signatures, C) Trajectory 2 and 4 cluster network analyses were plotted using ggplot2.

**Supplemental Figure S13. Pseudotime temporal progression of the minimal cytokine-activated signatures in significant disease trajectories from nasal/lung MCs.**

MC clusters from the *Sikkema et al.* single-cell dataset were extracted and re-analysed using the R package Seurat. A) Trajectory 2 and B) Trajectory 4 progression analyses of the individual cytokine-activated signatures along the pseudotime were plotted using ggplot2. Vertical grey dashed lines represent tertile-defined phase thresholds (Early-Mid-Late Trajectory 2, Early-Late Trajectory 4).

**Supplemental Figure S14. Differential expression of minimal IL-33 signature genes in nasal/lung single-cell MC clusters.** MC clusters from the *Sikkema et al.* single-cell dataset were extracted and re-analysed using the R package Seurat. Signature enrichment analysis was conducted using the minimal cytokine-activated signatures for IL-33 relative to each of the 24 identified MC clusters. Heatmap of all differentially enriched genes per cluster in the IL-33 signature (FDR p adjusted<0.05) was plotted using ComplexHeatmap.

**Supplemental Figure S15. IL-33- and TGFβ-induced MC signatures are increased in eosinophil-high COPD patients.** Minimal cytokine-activated signatures from uniquely expressed genes by single stimulations were selected using Elastic Net. (A) Gene expression was manually calculated using normalized counts of bronchial brushings of patients with chronic obstructive pulmonary disease without (n=17) and with (n=20) associated blood eosinophilia. (B) Correlation and linear regression analysis of IL-33 and TGFβ-induced signatures in eosinophil-high COPD.

(C) Representative immunofluorescent lung slide of n=1 COPD patient with normal (box left) and focal areas of epithelial remodelling with associated IL-33 production (box right). Arrows indicate IL-13-producing MCs (IL-13+Tryptase+).

Abbreviations: p^corr^: p value of correlation analysis, p^reg^: p value of linear regression, r: Spearman rho, r^2^: R-squared coefficient of determination.

**Supplemental Figure S16. IL-13 is a common indicator of IL-33 MC priming across available signatures.** Venn diagram of currently available signatures from *Tiotiu et al.*(46) and *Nagarkar et al.*(47), compared to *Badi et al.*(45) and the present study was computed using Venny.
