# Supplementary material for: Microenvironment‐Driven Mast Cell Plasticity: Insights From Cytokine‐Activated Gene Signatures in Skin and Respiratory Diseases
Source: Allergy. 2025 Sep 10;80(11):3077–94. doi: 10.1111/all.70052 (PMC12590347; doi:10.1111/all.70052)
Supplement: Supplementary file 2 — Appendix S2: all70052‐sup‐0002‐AppendixS2.pdf. [file ALL-80-3077-s002.pdf]

Figure S1
Experimental flow chart

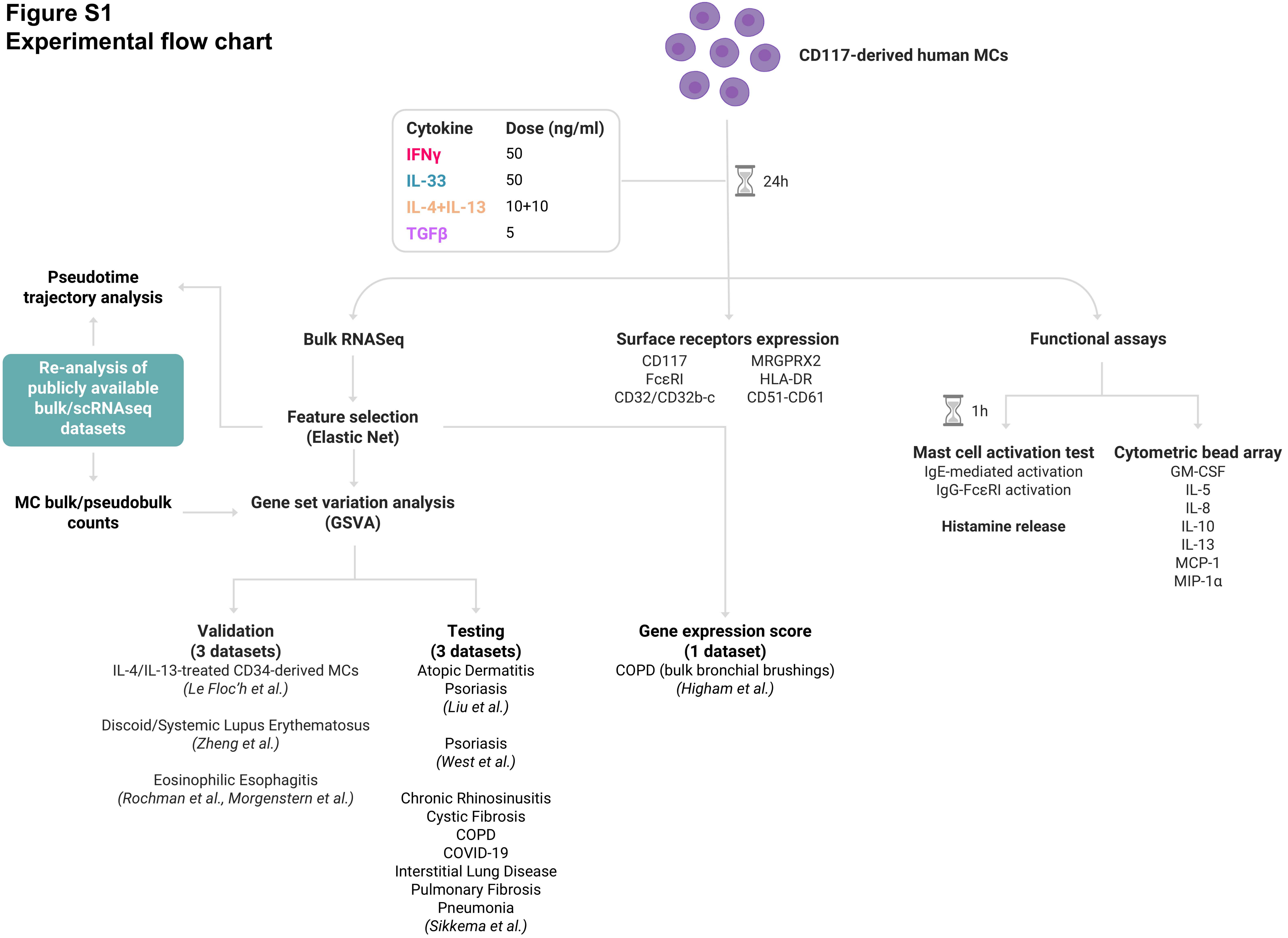

Figure S2

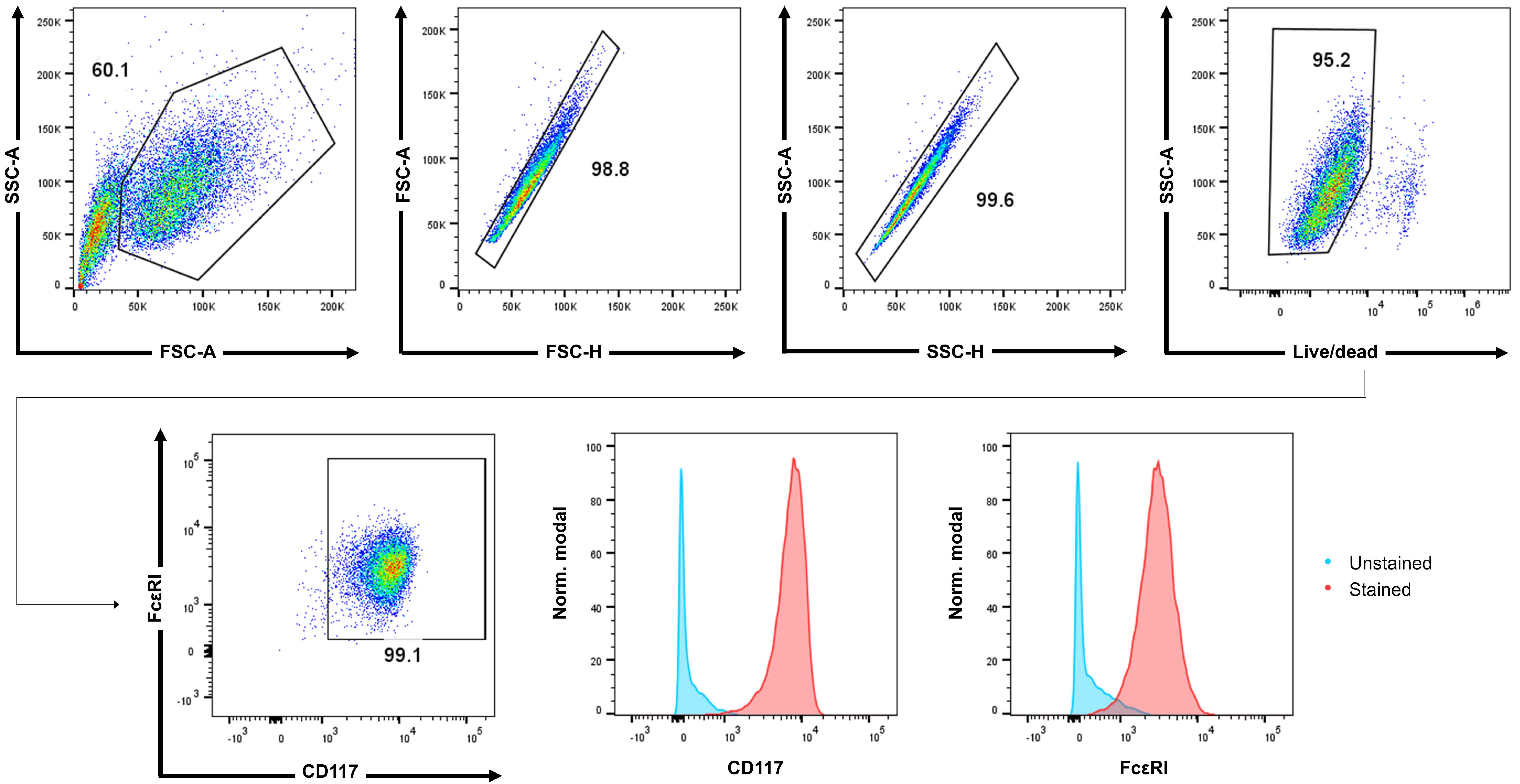

60.1
SSC-A
FSC-A
98.8
FSC-A
FSC-H
99.6
SSC-A
SSC-H
95.2
SSC-A
Live/dead
99.1
FcεRI
CD117
Norm. modal
CD117
Norm. modal
FcεRI
Unstained
Stained

Figure S3

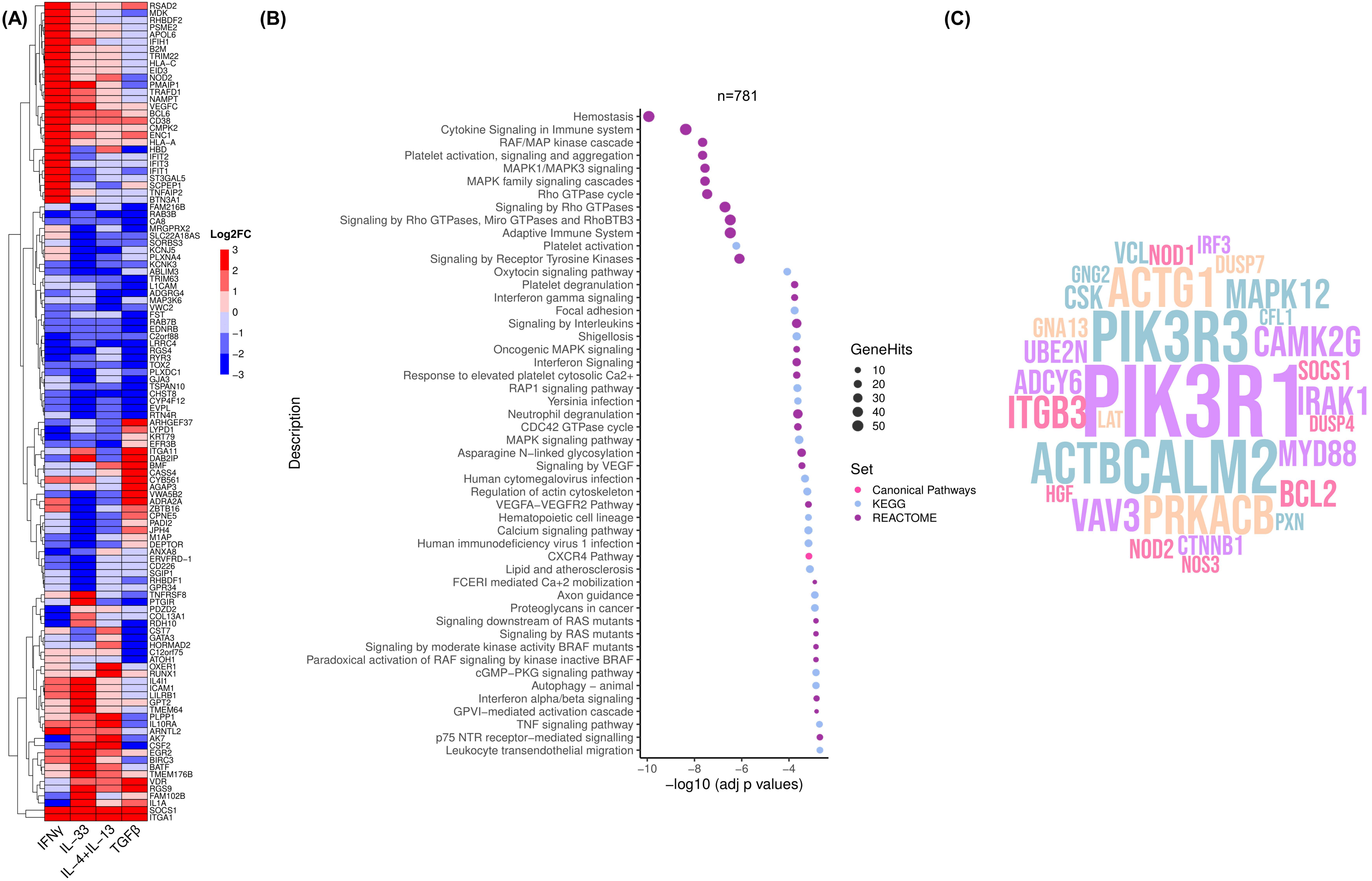

(A)
RSAD2
MDK
RHBDF2
PSME2
APOL6
IFIH1
B2M
TRIM22
HLA−C
EID3
NOD2
PMAIP1
TRAFD1
NAMPT
VEGFC
BCL6
CD38
CMPK2
ENC1
HLA−A
HBD
IFIT2
IFIT3
IFIT1
ST3GAL5
SCPEP1
TNFAIP2
BTN3A1
FAM216B
RAB3B
CA8
MRGPRX2
SLC22A18AS
SORBS3
KCNJ5
PLXNA4
KCNK3
ABLIM3
TRIM63
L1CAM
ADGRG4
MAP3K6
VWC2
FST
RAB7B
EDNRB
C2orf88
LRRC4
RGS4
RYR3
TOX2
PLXDC1
GJA3
TSPAN10
CHST8
CYP4F12
EVPL
RTN4R
ARHGEF37
LYPD1
KRT79
EFR3B
ITGA11
DAB2IP
BMF
CASS4
CYB561
AGAP3
VWA5B2
ADRA2A
ZBTB16
CPNE5
PADI2
JPH4
M1AP
DEPTOR
ANXA8
ERVFRD−1
CD226
SGIP1
RHBDF1
GPR34
TNFRSF8
PTGIR
PDZD2
COL13A1
RDH10
CST7
GATA3
HORMAD2
C12orf75
ATOH1
OXER1
RUNX1
IL4I1
ICAM1
LILRB1
GPT2
TMEM64
PLPP1
IL10RA
ARNTL2
AK7
CSF2
EGR2
BIRC3
BATF
TMEM176B
VDR
RGS9
FAM102B
IL1A
SOCS1
ITGA1
IFNγ
IL−33
IL−4+IL−13
TGFβ
Log2FC
3
2
1
0
−1
−2
−3
(B)
n=781
Hemostasis
Cytokine Signaling in Immune system
RAF/MAP kinase cascade
Platelet activation, signaling and aggregation
MAPK1/MAPK3 signaling
MAPK family signaling cascades
Rho GTPase cycle
Signaling by Rho GTPases
Signaling by Rho GTPases, Miro GTPases and RhoBTB3
Adaptive Immune System
Platelet activation
Signaling by Receptor Tyrosine Kinases
Oxytocin signaling pathway
Platelet degranulation
Interferon gamma signaling
Focal adhesion
Signaling by Interleukins
Shigellosis
Oncogenic MAPK signaling
Interferon Signaling
Response to elevated platelet cytosolic Ca2+
RAP1 signaling pathway
Yersinia infection
Neutrophil degranulation
CDC42 GTPase cycle
MAPK signaling pathway
Asparagine N−linked glycosylation
Signaling by VEGF
Human cytomegalovirus infection
Regulation of actin cytoskeleton
VEGFA−VEGFR2 Pathway
Hematopoietic cell lineage
Calcium signaling pathway
Human immunodeficiency virus 1 infection
CXCR4 Pathway
Lipid and atherosclerosis
FCERI mediated Ca+2 mobilization
Axon guidance
Proteoglycans in cancer
Signaling downstream of RAS mutants
Signaling by RAS mutants
Signaling by moderate kinase activity BRAF mutants
Paradoxical activation of RAF signaling by kinase inactive BRAF
cGMP−PKG signaling pathway
Autophagy − animal
Interferon alpha/beta signaling
GPVI−mediated activation cascade
TNF signaling pathway
p75 NTR receptor−mediated signalling
Leukocyte transendothelial migration
Description
−10
−8
−6
−4
−log10 (adj p values)
GeneHits
10
20
30
40
50
Set
Canonical Pathways
KEGG
REACTOME
(C)
VCL
NOD1
IRF3
DUSP7
GNG2
ACTG1
MAPK12
CSK
CFL1
GNA13
PIK3R3
CAMK2G
UBE2N
SOCS1
ADCY6
PIK3R1
IRAK1
ITGB3
LAT
DUSP4
ACTB
CALM2
MYD88
HGF
BCL2
VAV3
PRKACB
PXN
NOD2
CTNNB1
NOS3

Figure S4

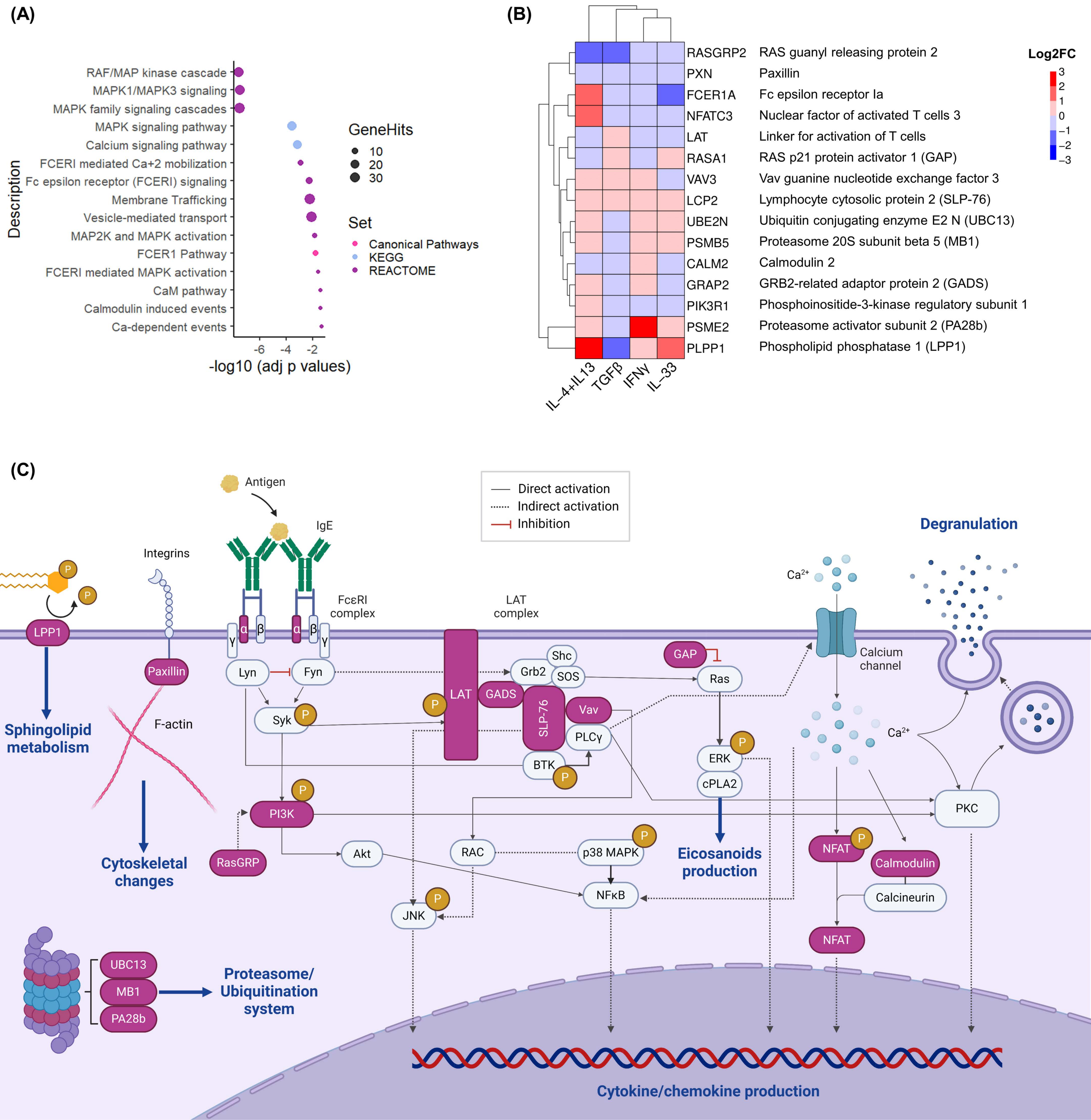

(A)
RAF/MAP kinase cascade
MAPK1/MAPK3 signaling
MAPK family signaling cascades
MAPK signaling pathway
Calcium signaling pathway
FCERI mediated Ca+2 mobilization
Fc epsilon receptor (FCERI) signaling
Membrane Trafficking
Vesicle-mediated transport
MAP2K and MAPK activation
FCER1 Pathway
FCERI mediated MAPK activation
CaM pathway
Calmodulin induced events
Ca-dependent events
Description
-6
-4
-2
-log10 (adj p values)
GeneHits
10
20
30
Set
Canonical Pathways
KEGG
REACTOME
(B)
RASGRP2 RAS guanyl releasing protein 2
PXN Paxillin
FCER1A Fc epsilon receptor Ia
NFATC3 Nuclear factor of activated T cells 3
LAT Linker for activation of T cells
RASA1 RAS p21 protein activator 1 (GAP)
VAV3 Vav guanine nucleotide exchange factor 3
LCP2 Lymphocyte cytosolic protein 2 (SLP-76)
UBE2N Ubiquitin conjugating enzyme E2 N (UBC13)
PSMB5 Proteasome 20S subunit beta 5 (MB1)
CALM2 Calmodulin 2
GRAP2 GRB2-related adaptor protein 2 (GADS)
PIK3R1 Phosphoinositide-3-kinase regulatory subunit 1
PSME2 Proteasome activator subunit 2 (PA28b)
PLPP1 Phospholipid phosphatase 1 (LPP1)
IL−4+IL13
TGFβ
IFNγ
IL−33
Log2FC
3
2
1
0
−1
−2
−3
(C)
Antigen
IgE
Direct activation
Indirect activation
Inhibition
Degranulation
Integrins
FcεRI complex
LAT complex
Ca^{2+}
Calcium channel
LPP1
P
Paxillin
γ
α
β
Lyn
Fyn
Syk
LAT
GADS
Grb2
Shc
SOS
SLP-76
Vav
PLCγ
BTK
GAP
Ras
ERK
cPLA2
F-actin
Sphingolipid metabolism
Cytoskeletal changes
PI3K
RasGRP
Akt
RAC
p38 MAPK
NFκB
JNK
Eicosanoids production
NFAT
Calmodulin
Calcineurin
PKC
UBC13
MB1
PA28b
Proteasome/ Ubiquitination system
Cytokine/chemokine production

Figure S5

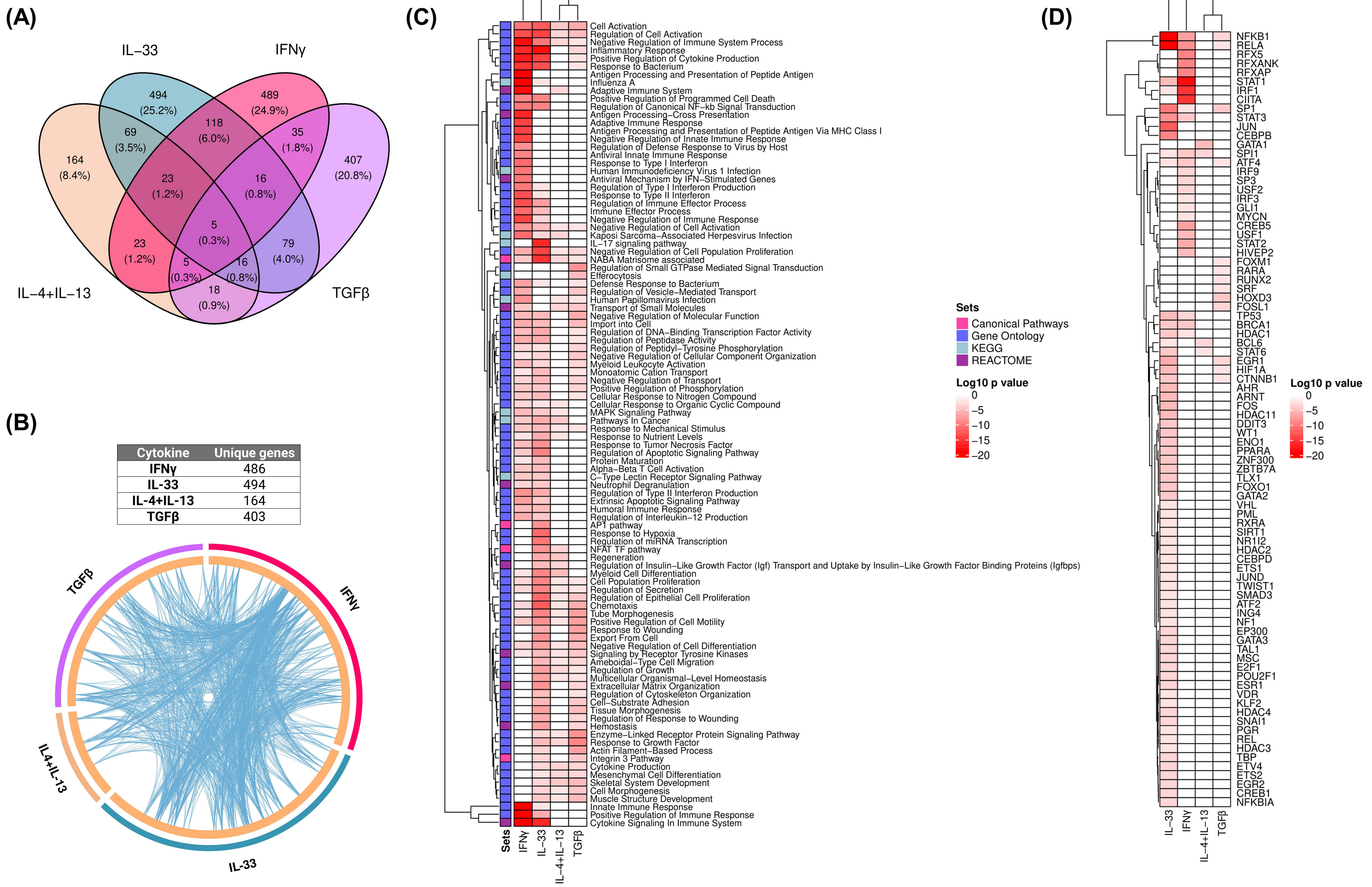

Cytokine	Unique genes
**IFNγ**	486
**IL-33**	494
**IL-4+IL-13**	164
**TGFβ**	403

Figure S6

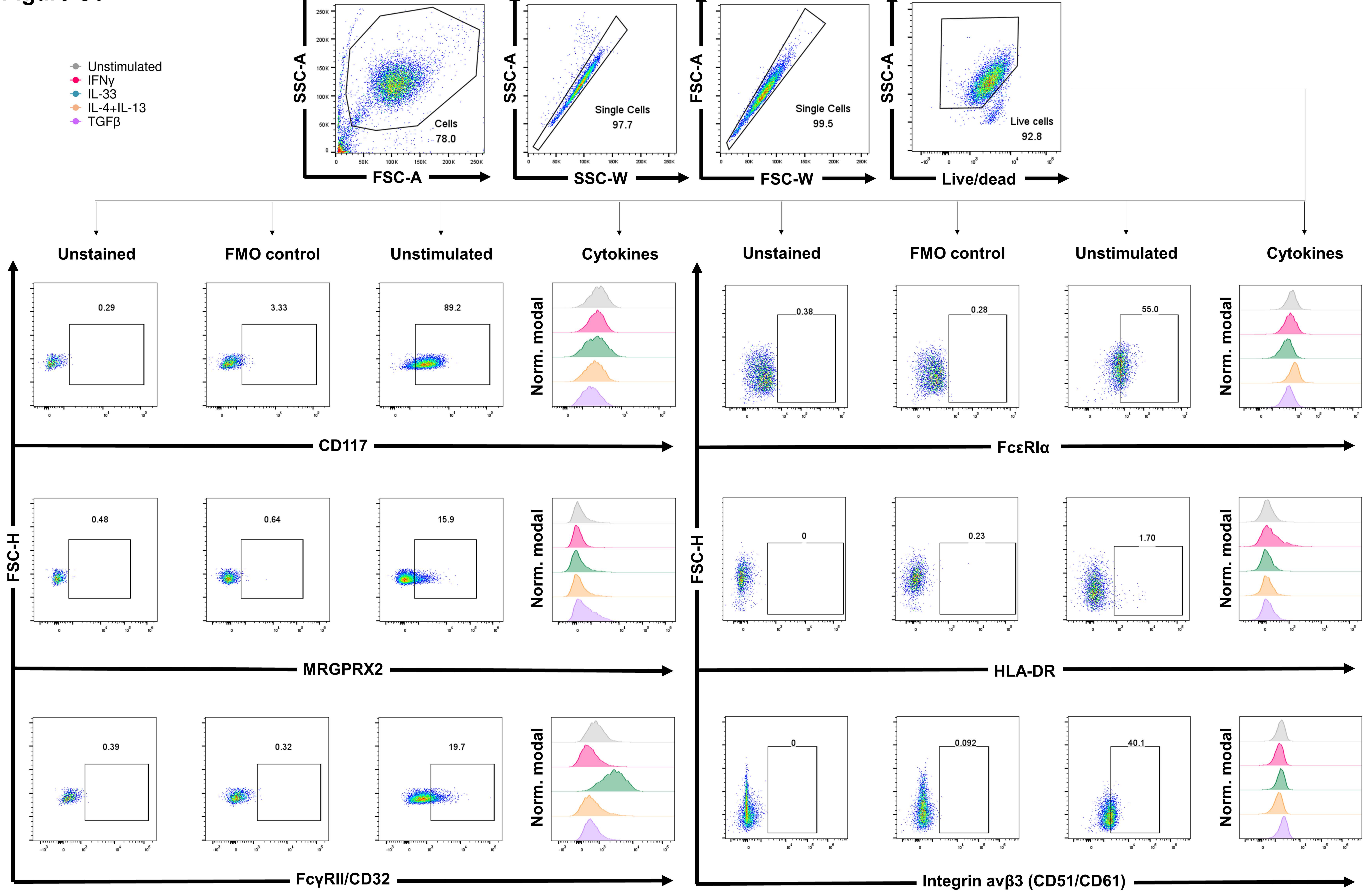

Unstimulated
IFNγ
IL-33
IL-4+IL-13
TGFβ
SSC-A
FSC-A
Cells
78.0
SSC-W
Single Cells
97.7
FSC-W
Single Cells
99.5
Live/dead
Live cells
92.8
Unstained
FMO control
Unstimulated
Cytokines
0.29
3.33
89.2
Norm. modal
CD117
0.38
0.28
55.0
FcεRIα
0.48
0.64
15.9
MRGPRX2
0
0.23
1.70
HLA-DR
0.39
0.32
19.7
FcγRII/CD32
0
0.092
40.1
Integrin avβ3 (CD51/CD61)
FSC-H

Figure S7

(A)

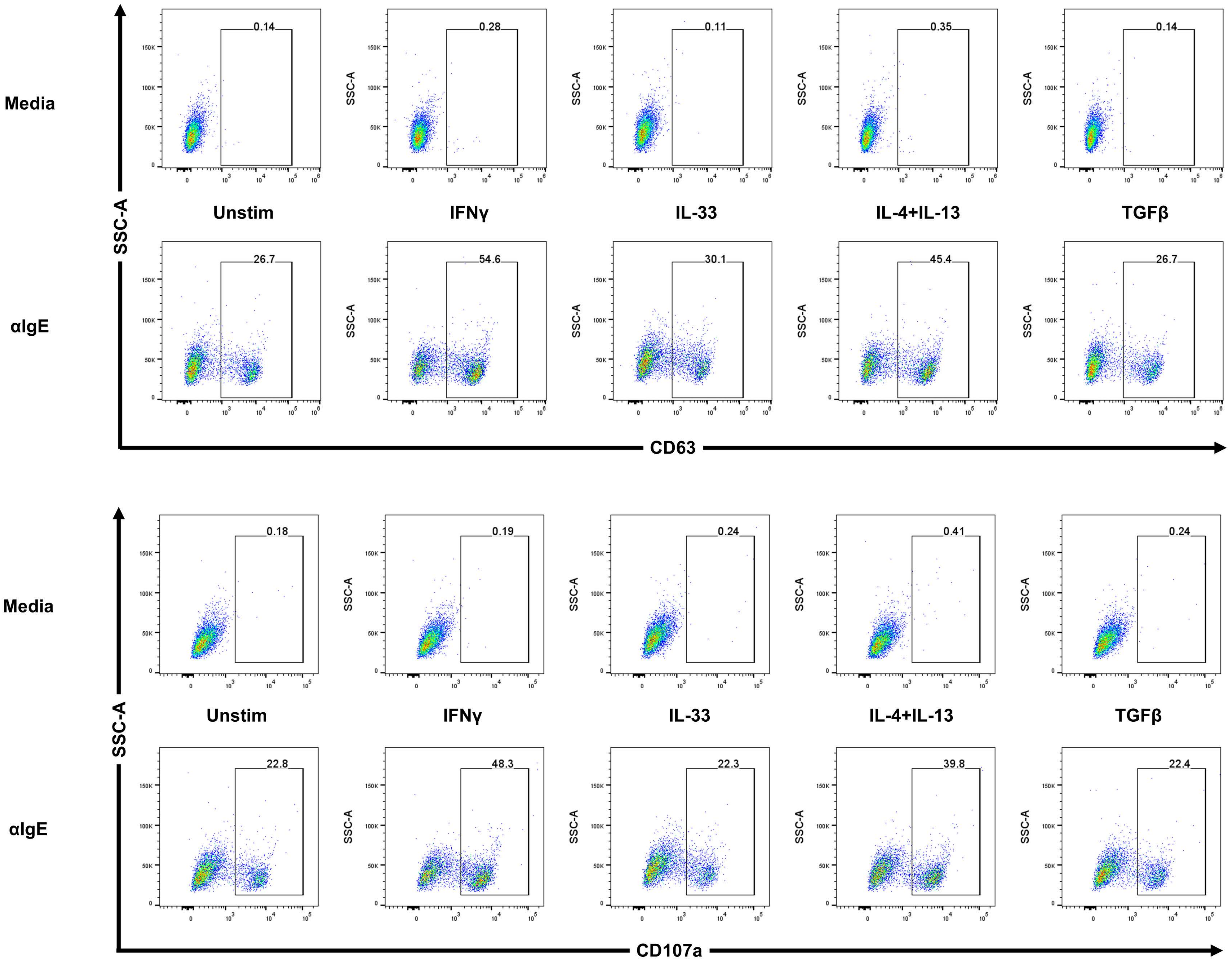

(B)

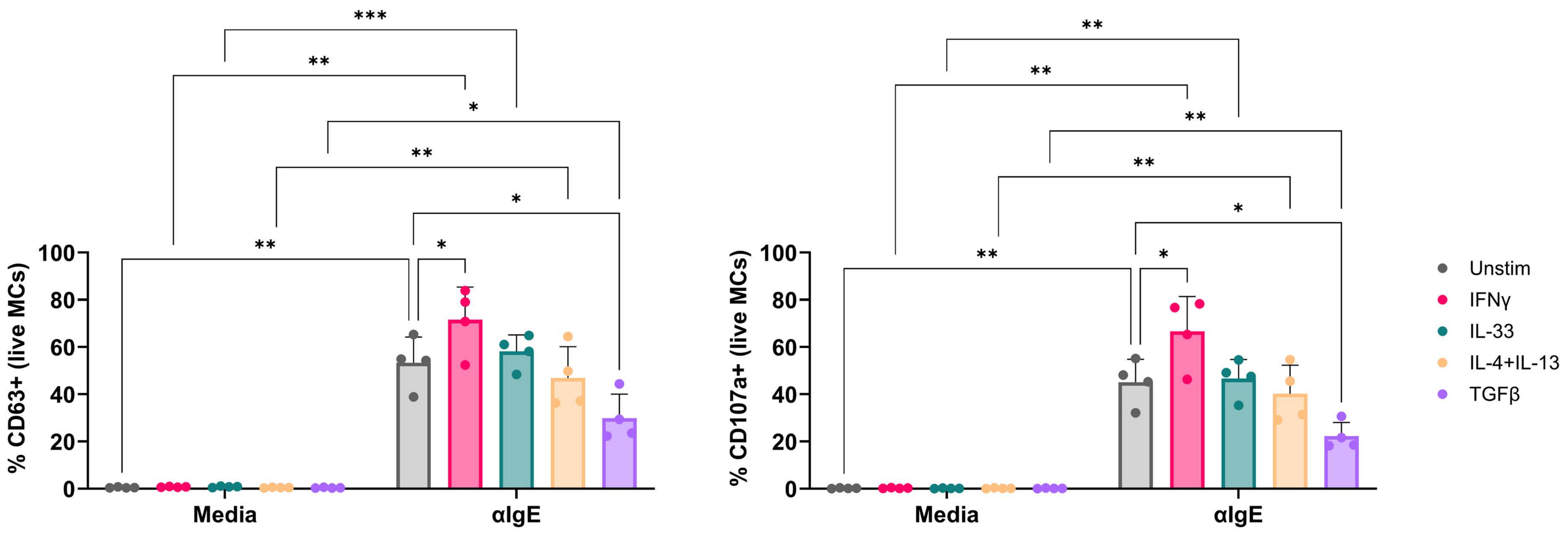

(C)

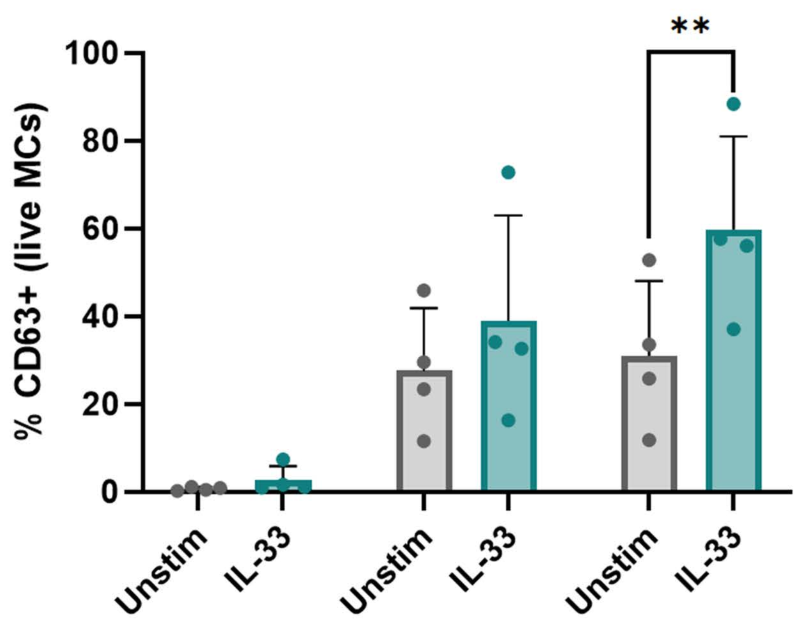

Anti-FcεRIα	-	+	+
Anti-FcγR IgG	+	+	+
IgG2a κ antibodies	-	-	+
Anti-IgG λ chain	-	+	+

Figure S8

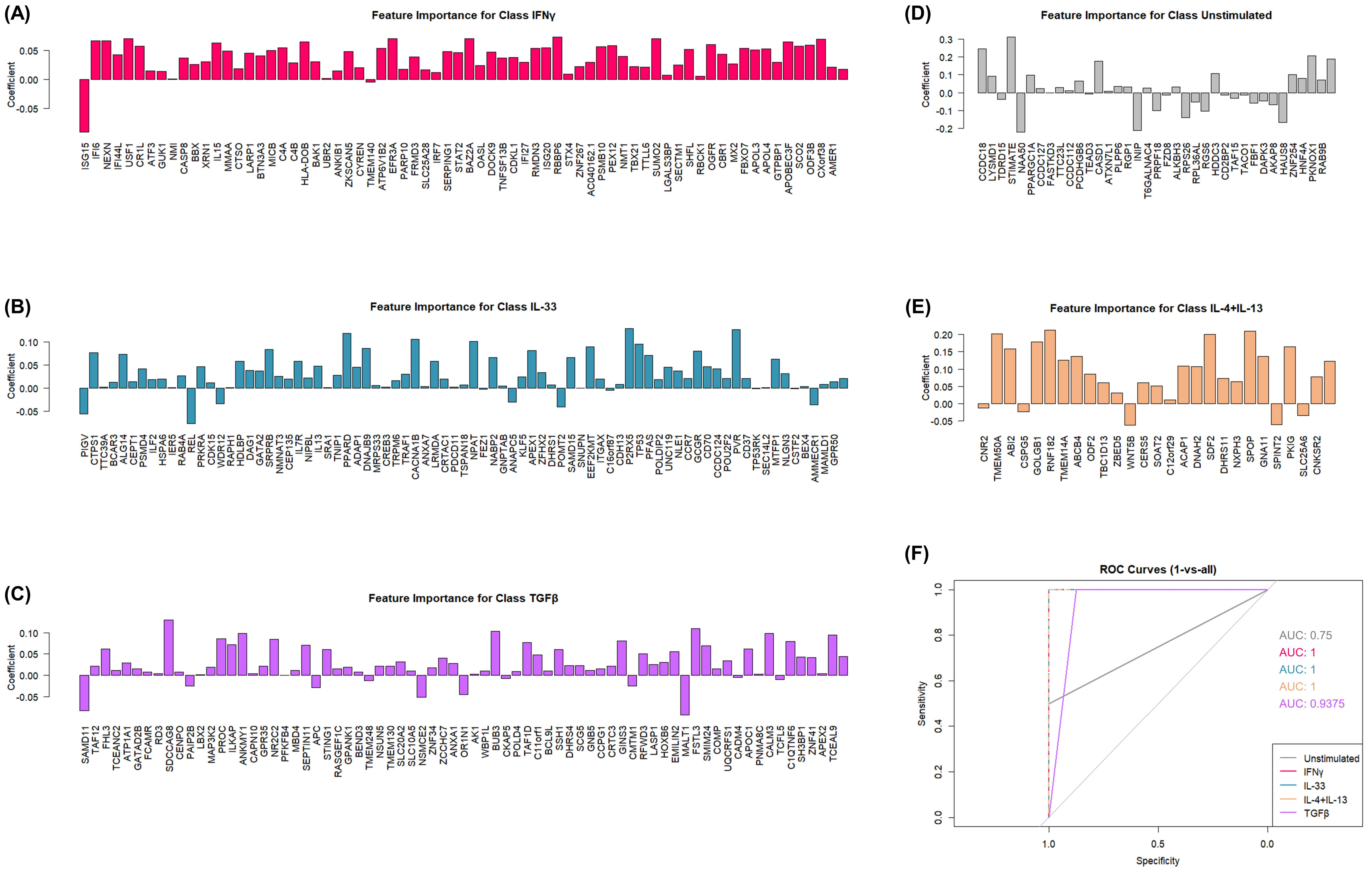

(A)
Feature Importance for Class IFNγ
Coefficient
0.05
0.00
-0.05
ISG15 IFI6 NEXN IFI44L USF1 CR1L ATF3 GUK1 NMI CASP8 BBX XRN1 IL15 MMAA CTSO LARP1 BTN3A3 MICB C4A C4B HLA-DOB BAK1 UBR2 ANKIB1 ZKSCAN5 CYREN TMEM140 ATP6V1B2 EFR3A PARP10 FRMD3 SLC25A28 IRF7 SERPING1 STAT2 BAZ2A OASL DOCK9 TNFSF13B CDKL1 IFI27 RMDN3 ISG20 RBBP6 STX4 ZNF267 AC040162.1 PSMB10 PEX12 NMT1 TBX21 TTLL6 SUMO2 LGALS3BP SECTM1 SHFL RBCK1 OGFR CBR1 MX2 FBXO7 APOL3 APOL4 GTPBP1 APOBEC3F SCO2 ODF3B CXorf38 AMER1
(B)
Feature Importance for Class IL-33
Coefficient
0.10
0.05
0.00
-0.05
PIGV CTPS1 TTC39A BCAR3 ALG14 CEPT1 PSMD4 ILF2 HSPA6 IER5 RAB4A REL PRKRA CDK15 WDR12 RAPH1 HDLBP DAG1 GATA2 SRPRB NMNAT3 CEP135 IL7R NIPBL IL13 SRA1 TNIP1 PPARD ADAP1 DNAJB9 MRPS33 CREB3 TRPM6 TRAF1 CACNA1B ANXA7 LRMDA CRTAC1 PDCD11 TSPAN18 NPAT FEZ1 NABP2 GNPTAB ANAPC5 KLF5 APEX1 ZFHX2 DHRS1 POMT2 SAMD15 SNUPN EEF2KMT ITGAX C16orf87 CDH13 P2RX5 TP53 PFAS POLDIP2 UNC119 NLE1 CCR7 GCGR CD70 CCDC124 POU2F2 PVR CD37 TP53RK SEC14L2 MTFP1 NLGN3 CSTF2 BEX4 AMMECR1 MAMLD1 GPR50
(C)
Feature Importance for Class TGFβ
Coefficient
0.10
0.05
0.00
-0.05
SAMD11 TAF12 FHL3 TCEANC2 ATP1A1 GATAD2B FCAMR RD3 SDCCAG8 CENPO PAIP2B LBX2 MAP3K2 PROC ILKAP ANKMY1 CAPN10 GPR35 NR2C2 PFKFB4 MBD4 SEPTIN11 APC STING1 RASGEF1C GPANK1 BEND3 TMEM248 NSUN5 TMEM130 SLC20A2 SLC10A5 NSMCE2 ZNF34 ZCCHC7 ANXA1 OR1N1 AK1 WBP1L BUB3 CKAP5 POLD4 TAF1D C11orf1 BCL9L SSH1 DHRS4 SGS5 GNB5 CCPG1 CRTC3 GINS3 CMTM1 RFWD3 LASP1 HOXB6 EMILIN2 MALT1 FSTL3 SMIM24 COMP UQCRFS1 CADM4 APOC1 PNMA8C CALM3 TCFL5 C1QTNF6 SH3BP1 ZNF41 APEX2 TCEAL9
(D)
Feature Importance for Class Unstimulated
Coefficient
0.3
0.2
0.1
0.0
-0.1
-0.2
CCDC18 LYSMD1 TDRD15 STIMATE NAA50 PPARGC1A CCDC127 FASTKD3 TTC23L CCDC112 PCDHGB6 TEAD3 CASD1 ATXN7L1 PLPP6 RGP1 INIP T6GALNAC4 PRPF18 FZD8 ALKBH3 RPS26 RPL36AL RGS6 HDDC3 CD2BP2 TAF15 TACO1 FBF1 DAPK3 AKAP8 HAUS8 ZNF254 HNF4A PKNOX1 RAB9B
(E)
Feature Importance for Class IL-4+IL-13
Coefficient
0.20
0.15
0.10
0.05
0.00
-0.05
CNR2 TMEM50A ABI2 CSPG5 GOLGB1 RNF182 TMEM14A ABCB4 ODF2 TBC1D13 ZBED5 WNT5B CERS5 SOAT2 C12orf29 ACAP1 DNAH2 SDF2 DHRS11 NXPH3 SPOP GNA11 SPINT2 PKIG SLC25A6 CNKSR2
(F)
ROC Curves (1-vs-all)
Sensitivity
0.0
0.2
0.4
0.6
0.8
1.0
Specificity
1.0
0.5
0.0
AUC: 0.75
AUC: 1
AUC: 1
AUC: 1
AUC: 0.9375
Unstimulated
IFNγ
IL-33
IL-4+IL-13
TGFβ

Figure S9

(A) CD133-derived primary MCs – IL-4 and IL-13 signatures (GSE140900 - Le Floc'h et al. 2020)

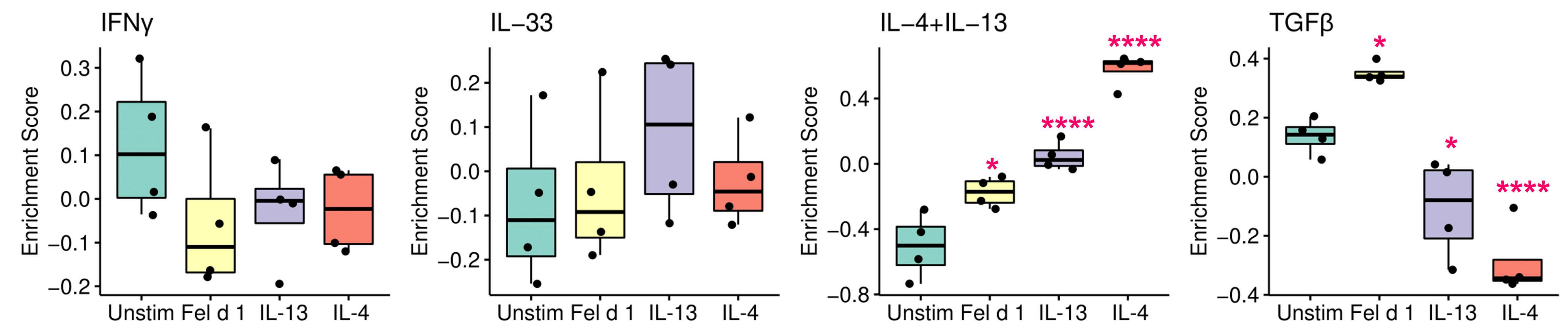

(B) Esophageal MCs – IL-33 and TGFβ signatures (GSE201153 - Rochman et al. 2022, Morgenstern et al. 2022)

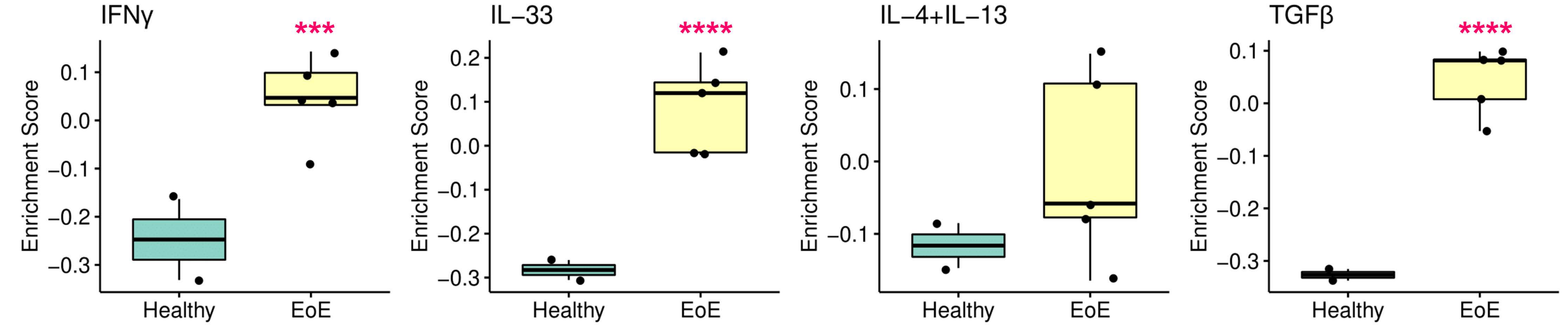

(C) Skin MCs – IFNγ signature (GSE179633 - Zheng et al. 2024)

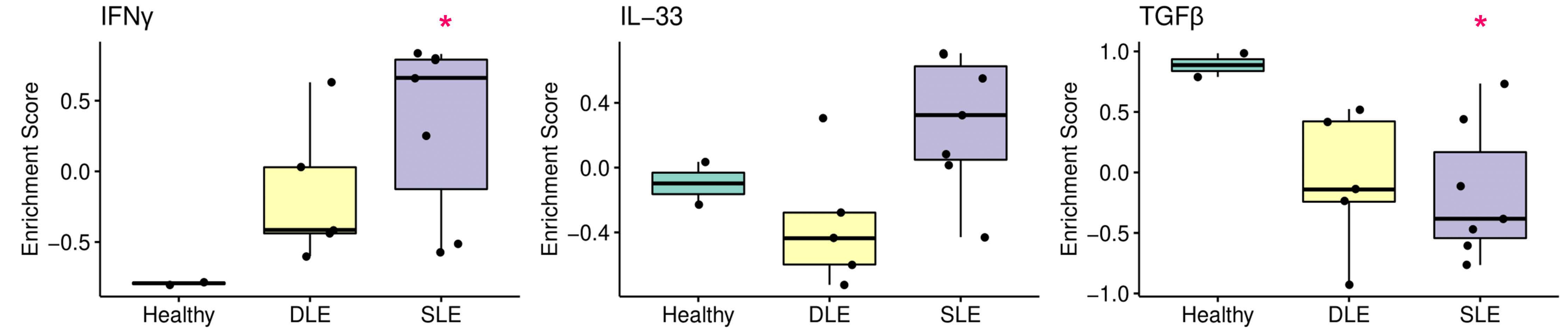

(D)

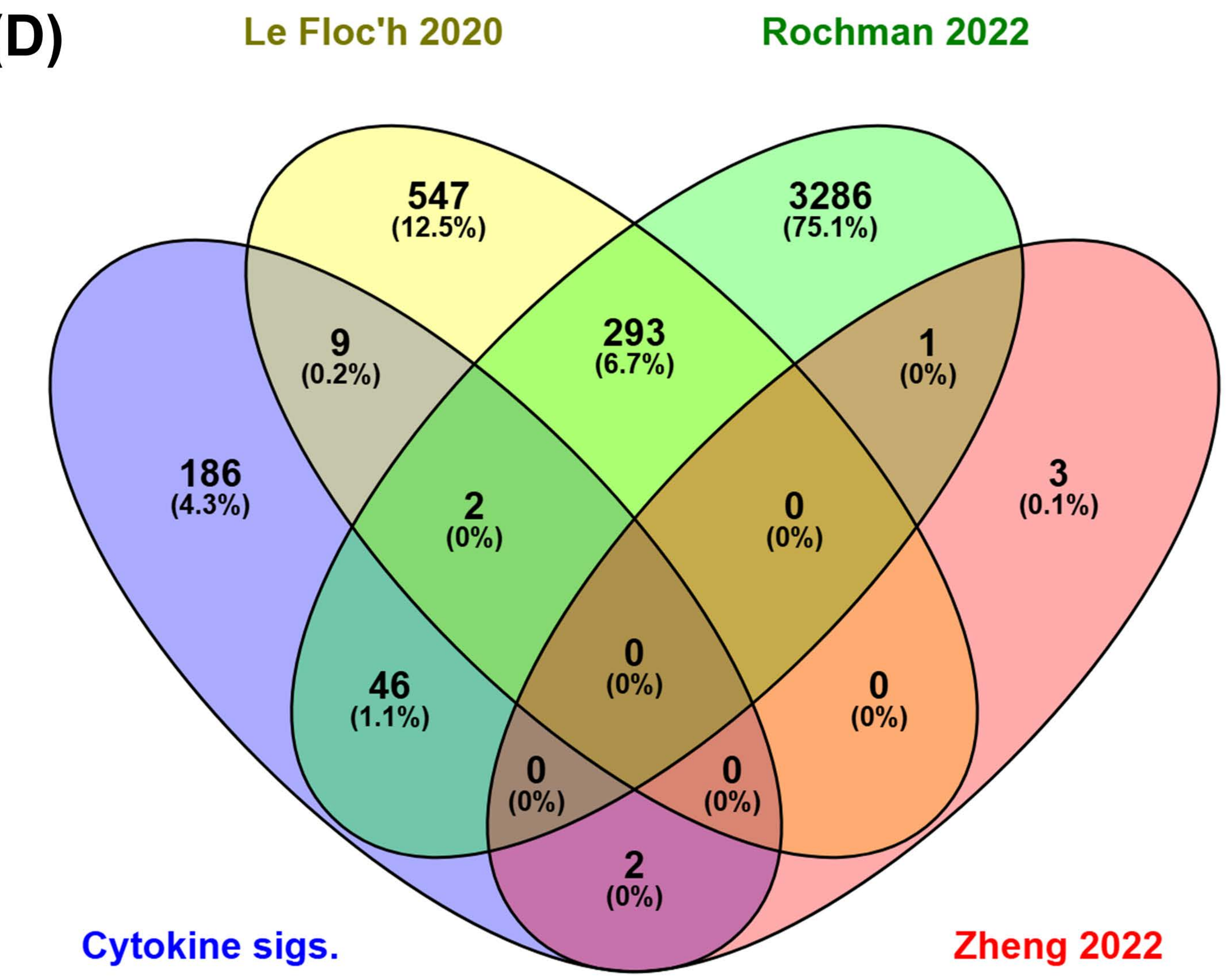

(E)

IFNγ	IL-33	IL-4+IL-13	TGFβ
ANKIB1	ALG14	ABI2	AK1
APOL4	AMMECR1	CERS5	ANXA1
BAK1	CCDC124	GOLGB1	C11orf1
BBX	CDK15	ODF2	CKAP5
GTPBP1	CEPT1	SLC25A6	EMILIN2
IFI27	DAG1	SPINT2	GATAD2B
IRF7	DHRS1	TMEM50A	GPR35
ISG15	DNAJB9	ZBED5	HOXB6
LARP1	HSPA6		LASP1
OGFR	IL7R		MAP3K2
RMDN3	ITGAX		SCG5
STAT2	KLF5		SDCCAG8
STX4	MTFP1		SH3BP1
UBR2	NABP2		ZCCHC7
XRN1	NIPBL		
	RAB4A		
	SEC14L2		
	SNUPN		
	SRPRB		
	TRAF1		
	UNC119		
	WDR12		

Figure S10

Bulk RNASeq – Isolated skin MCs (GSE217060 - West et al. 2024)

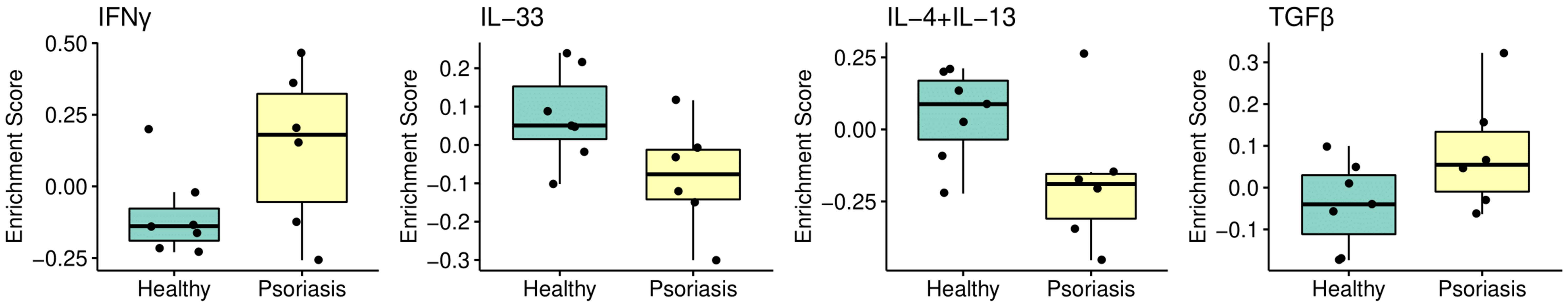

Figure S11

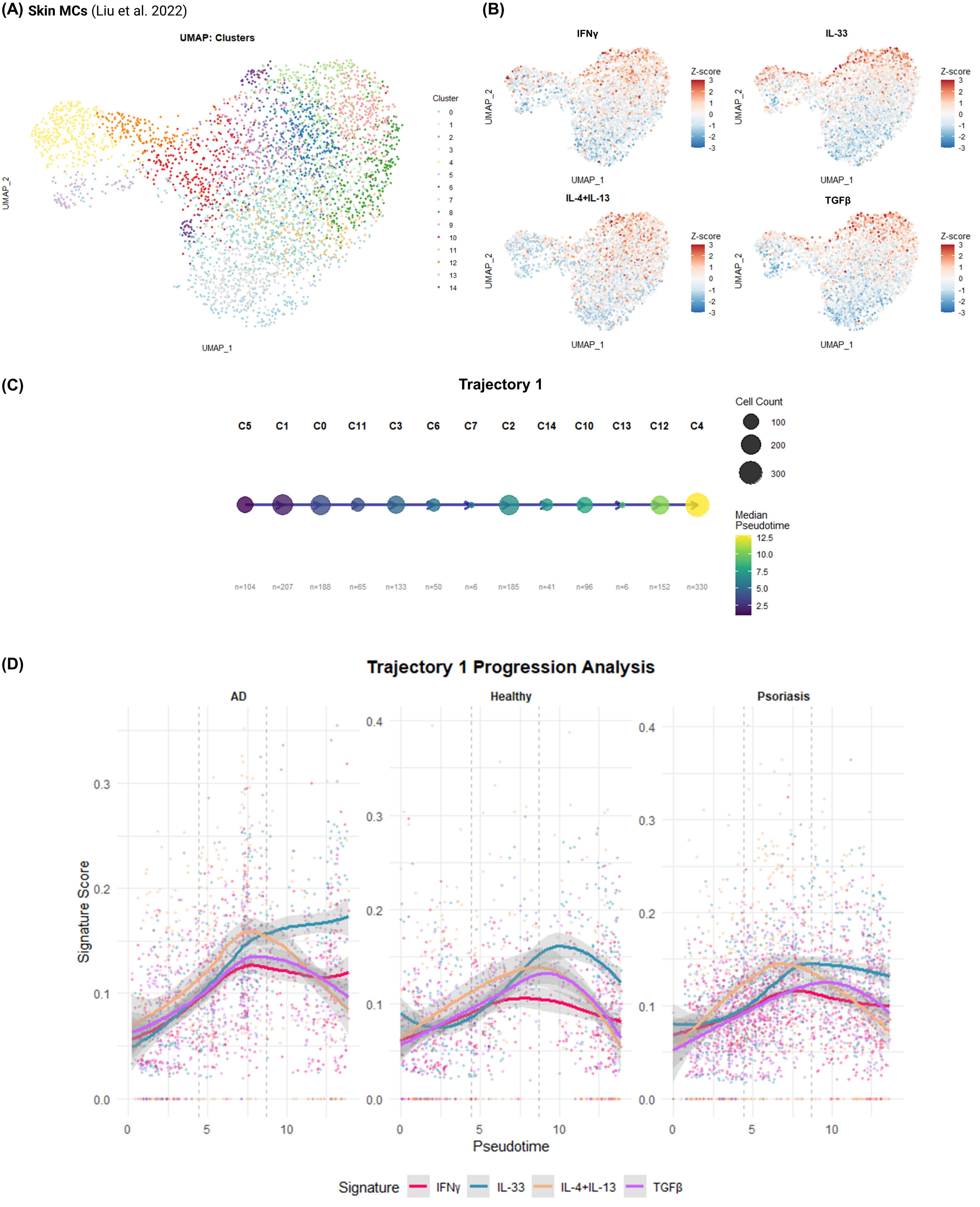

(A) Skin MCs (Liu et al. 2022)
UMAP: Clusters
Cluster
0
1
2
3
4
5
6
7
8
9
10
11
12
13
14
UMAP_1
UMAP_2
(B)
IFNγ
IL-33
IL-4+IL-13
TGFβ
Z-score
UMAP_1
UMAP_2
(C)
Trajectory 1
C5
C1
C0
C11
C3
C6
C7
C2
C14
C10
C13
C12
C4
n=104
n=207
n=188
n=65
n=133
n=50
n=6
n=185
n=41
n=96
n=6
n=152
n=330
Cell Count
100
200
300
Median Pseudotime
12.5
10.0
7.5
5.0
2.5
(D)
Trajectory 1 Progression Analysis
AD
Healthy
Psoriasis
Signature Score
Pseudotime
Signature
IFNγ
IL-33
IL-4+IL-13
TGFβ

Figure S12

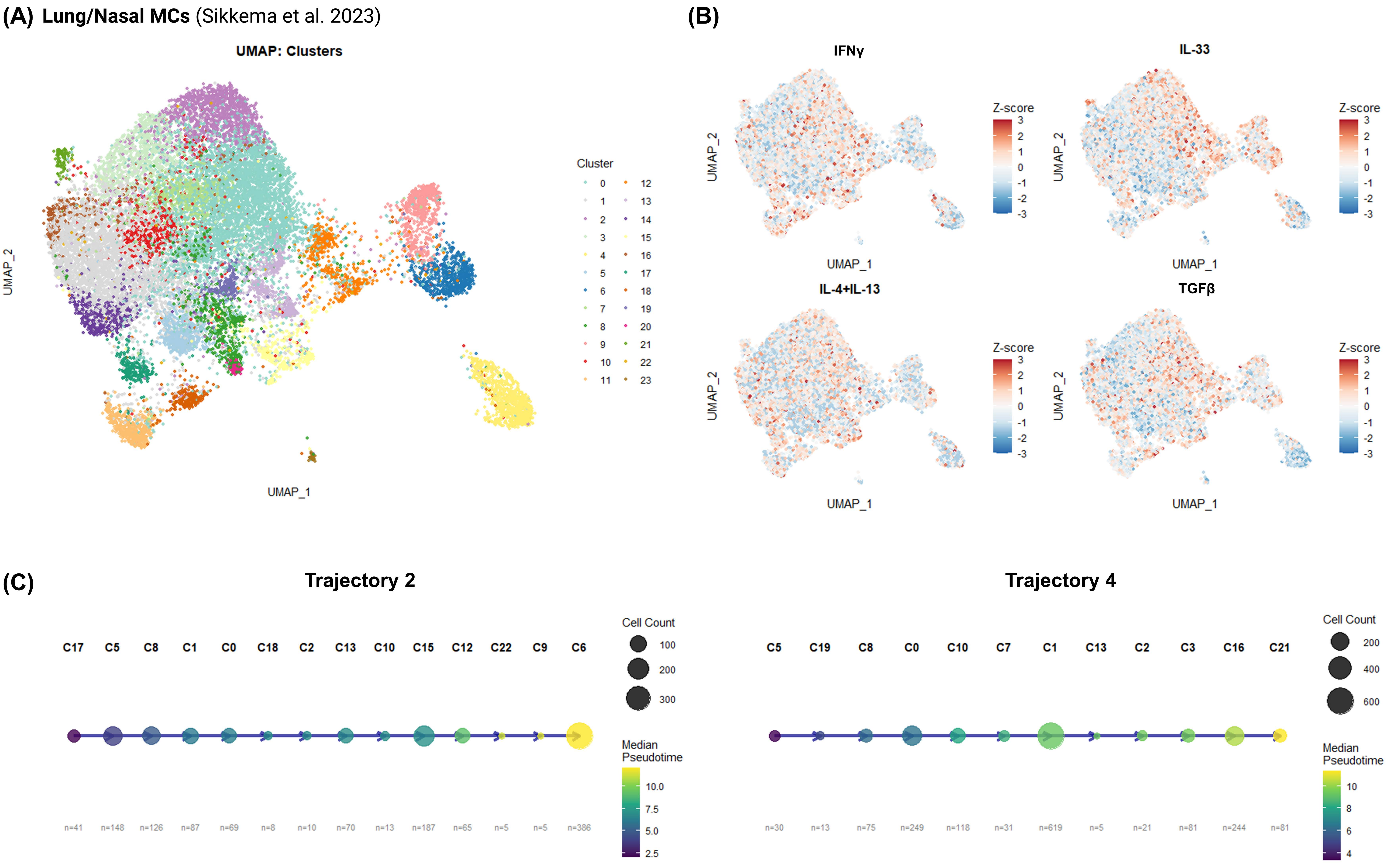
(A) Lung/Nasal MCs (Sikkema et al. 2023)
UMAP: Clusters
UMAP_2
UMAP_1
Cluster
0
1
2
3
4
5
6
7
8
9
10
11
12
13
14
15
16
17
18
19
20
21
22
23
(B)
IFNγ
IL-33
IL-4+IL-13
TGFβ
UMAP_2
UMAP_1
Z-score
3
2
1
0
-1
-2
-3
(C)
Trajectory 2
C17 C5 C8 C1 C0 C18 C2 C13 C10 C15 C12 C22 C9 C6
n=41 n=148 n=126 n=87 n=69 n=8 n=10 n=70 n=13 n=187 n=65 n=5 n=5 n=386
Cell Count
100
200
300
Median Pseudotime
10.0
7.5
5.0
2.5
Trajectory 4
C5 C19 C8 C0 C10 C7 C1 C13 C2 C3 C16 C21
n=30 n=13 n=75 n=249 n=118 n=31 n=619 n=5 n=21 n=81 n=244 n=81
Cell Count
200
400
600
Median Pseudotime
10
8
6
4

Figure S13

(A) Lung/Nasal MCs (Sikkema et al. 2023)

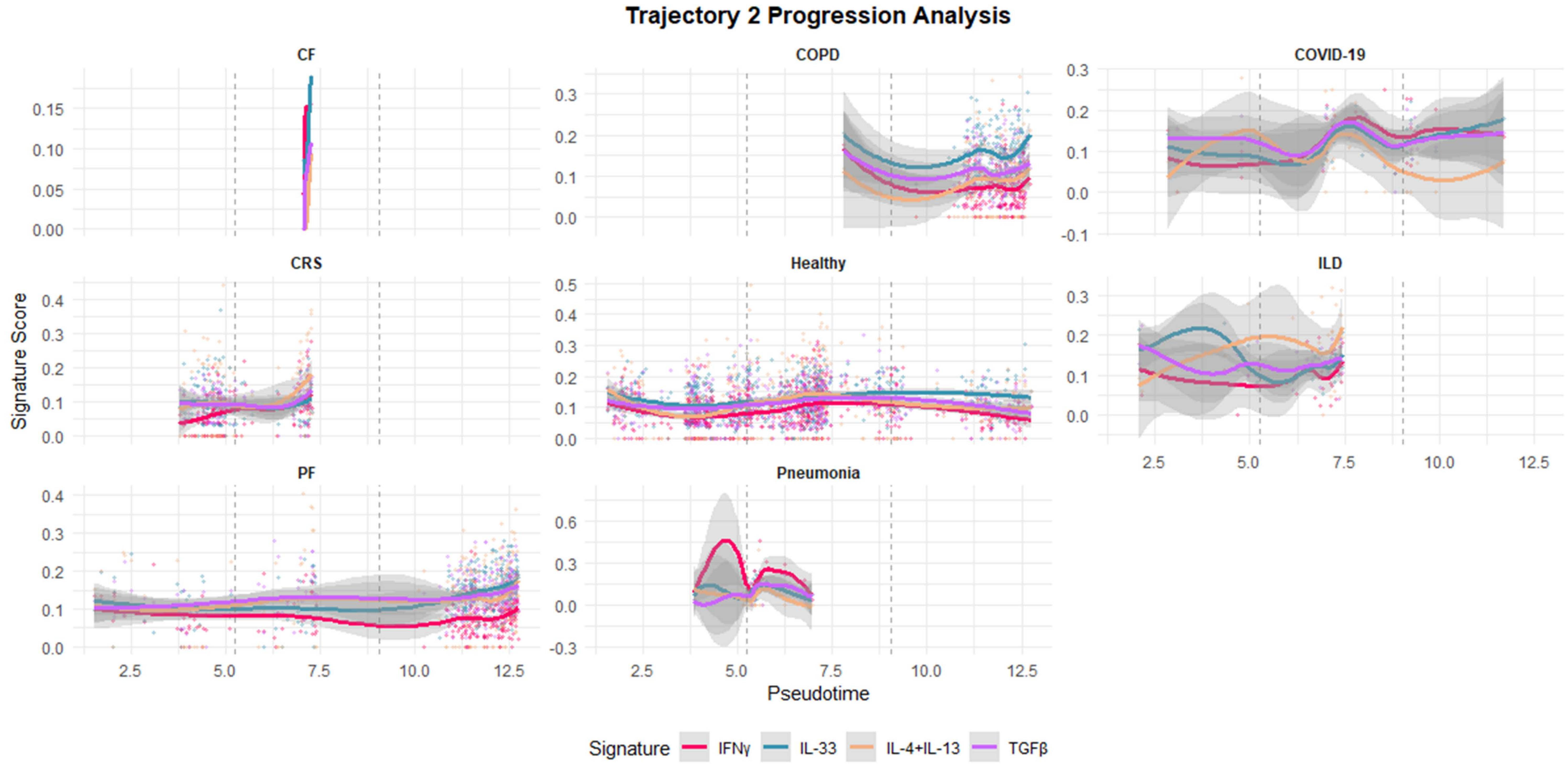

(B)

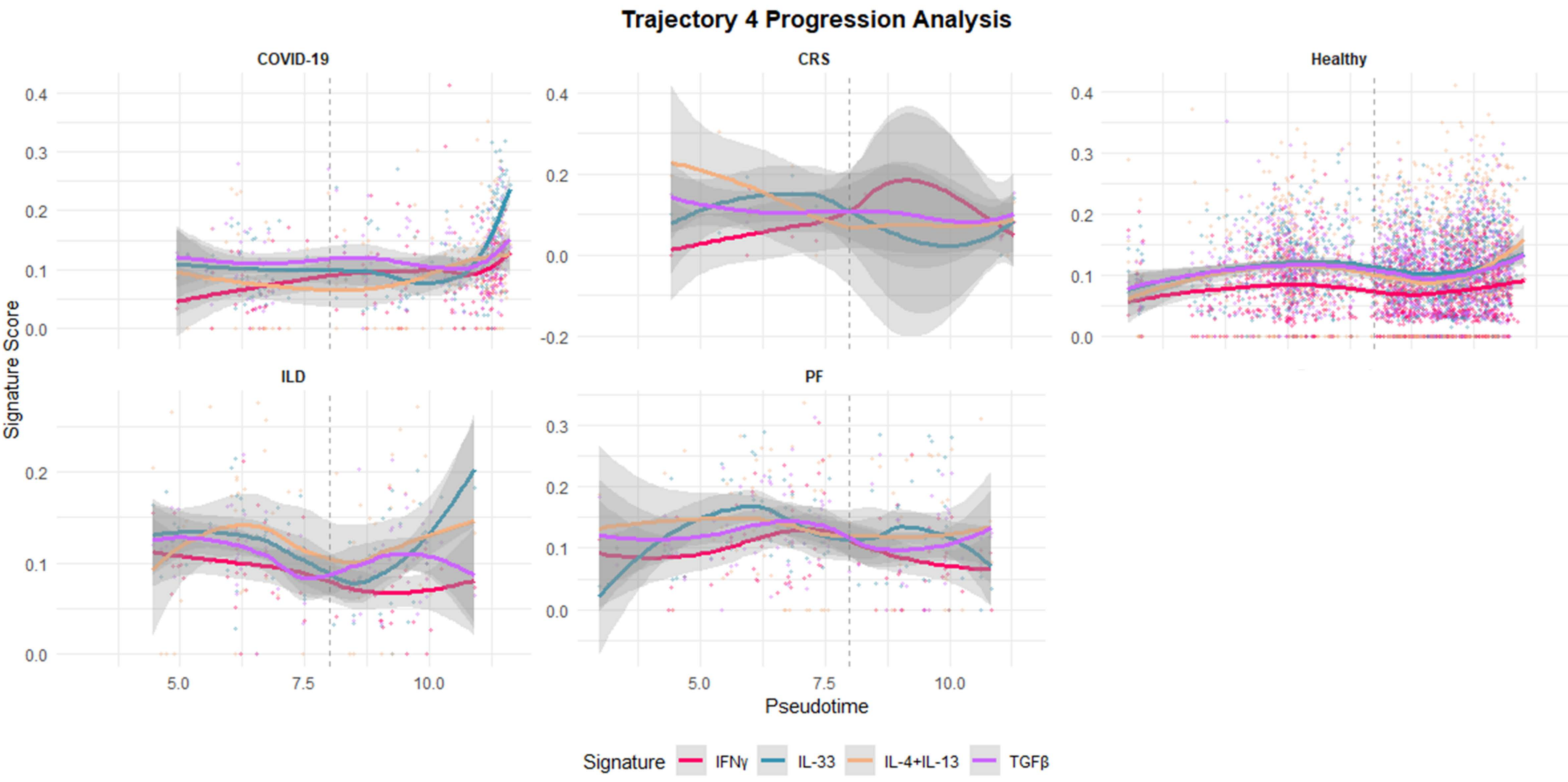

Figure S14

Lung/Nasal MCs (Sikkema et al. 2023)

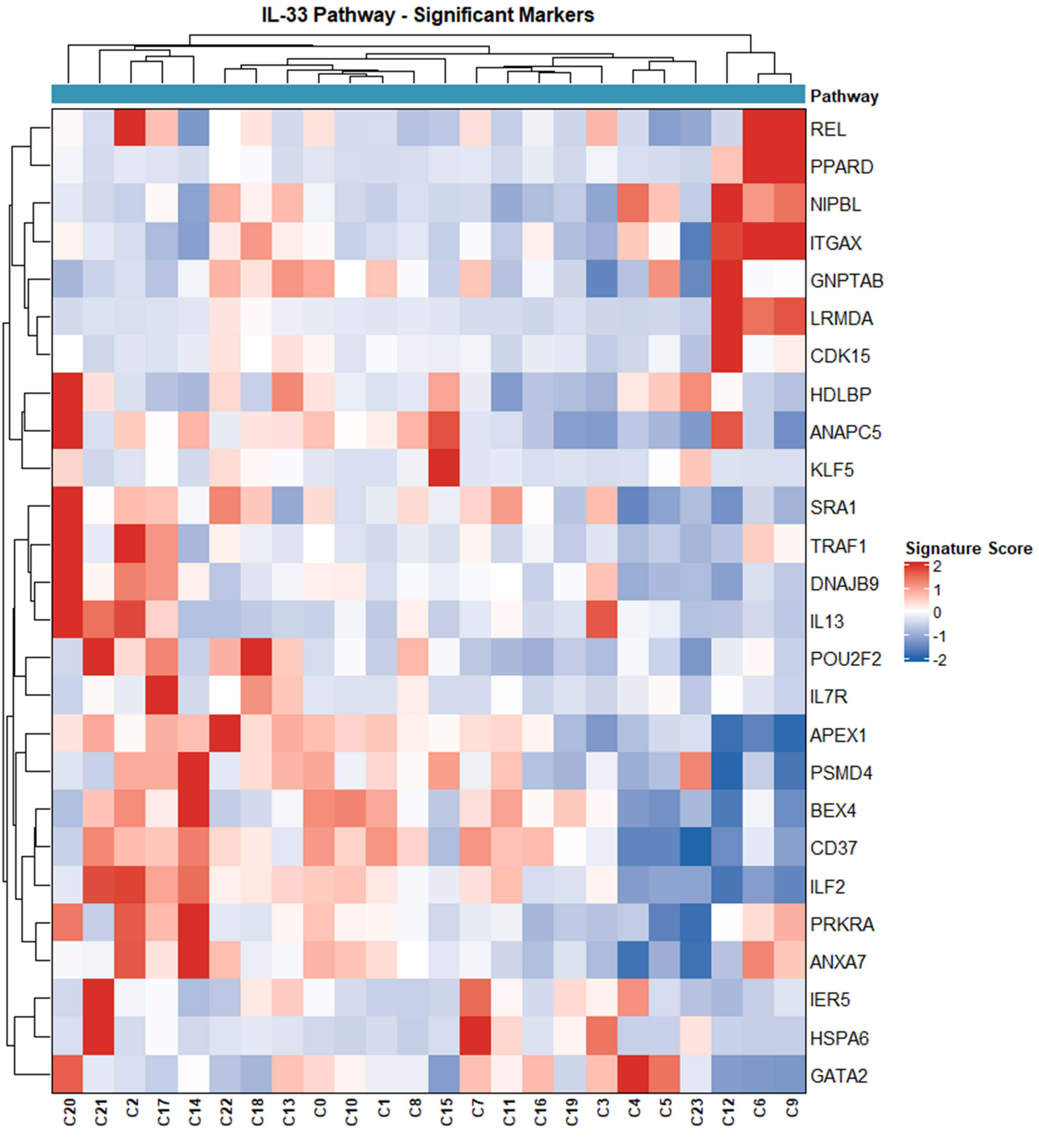

Figure S15

Bulk RNASeq – COPD bronchial brushings (Higham et al. 2022)

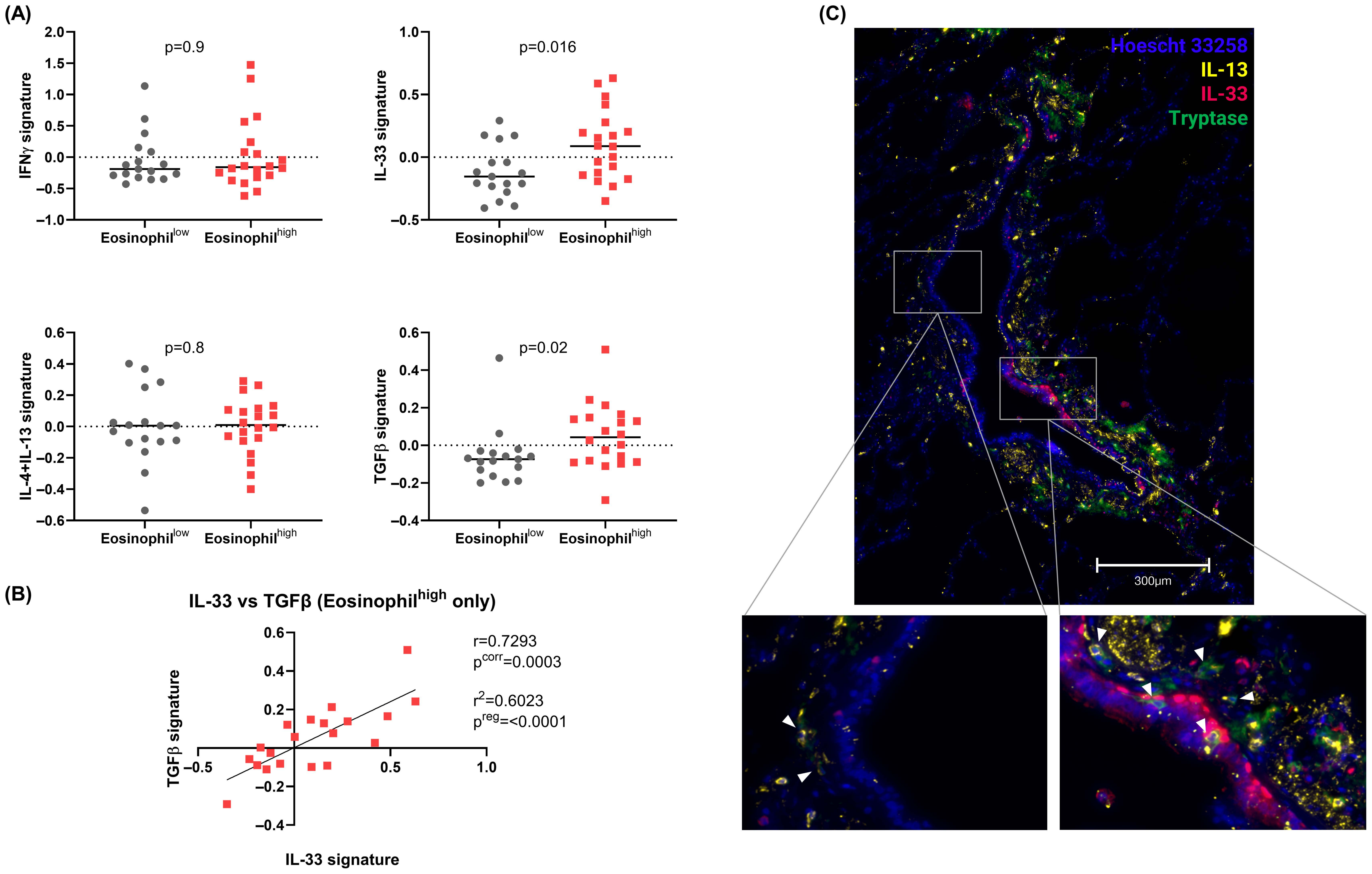

Figure S16

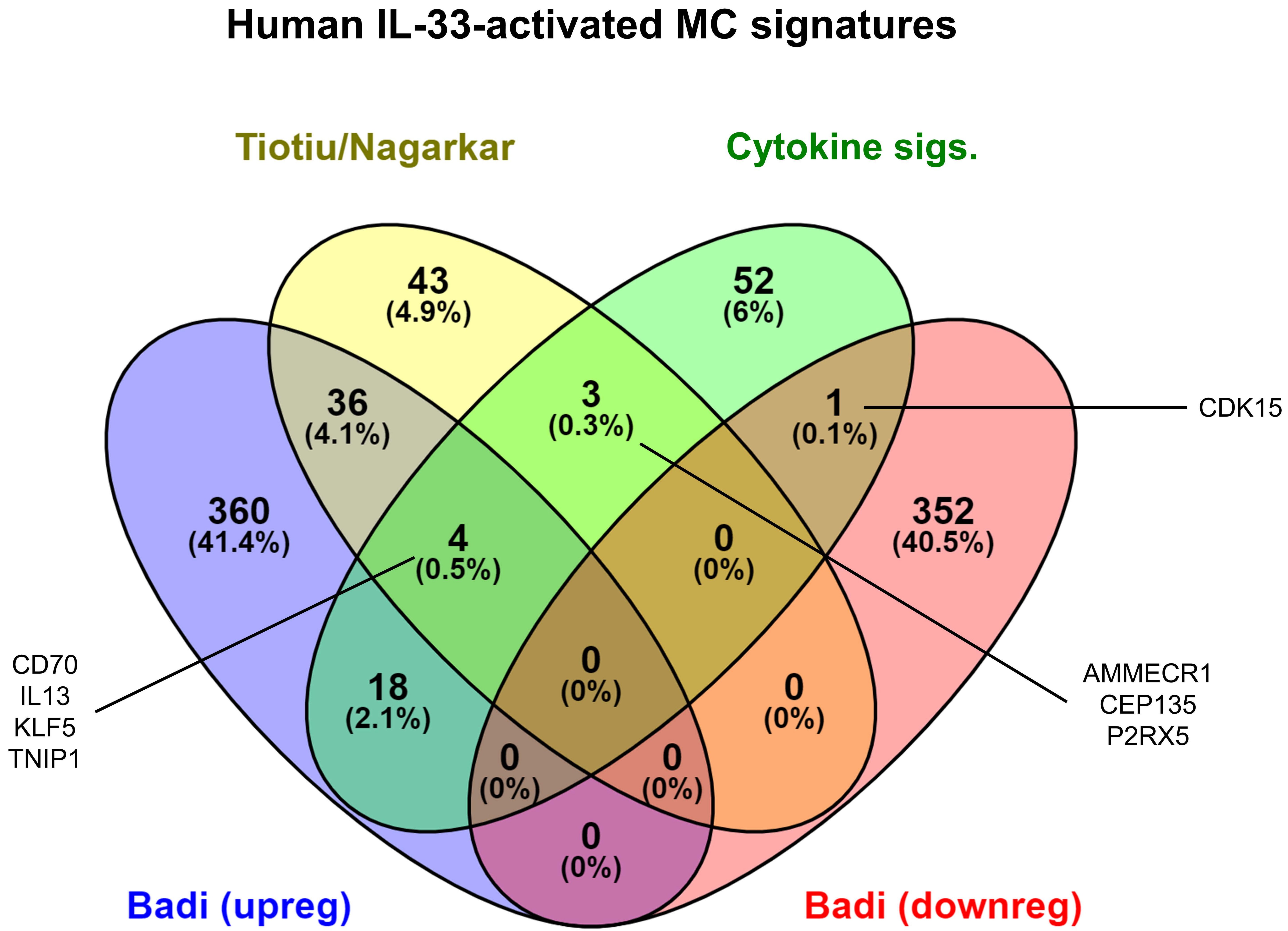
Human IL-33-activated MC signatures
Tiotiu/Nagarkar
Cytokine sigs.
43
(4.9%)
52
(6%)
36
(4.1%)
3
(0.3%)
1
(0.1%)
CDK15
360
(41.4%)
4
(0.5%)
0
(0%)
352
(40.5%)
CD70
IL13
KLF5
TNIP1
18
(2.1%)
0
(0%)
0
(0%)
AMMECR1
CEP135
P2RX5
0
(0%)
0
(0%)
0
(0%)
Badi (upreg)
Badi (downreg)
